# Advanced multiparametric image spectroscopy and super-resolution microscopy reveal a minimal model of CD95 signal initiation

**DOI:** 10.1126/sciadv.adn3238

**Published:** 2024-08-30

**Authors:** Nina Bartels, Nicolaas T. M. van der Voort, Oleg Opanasyuk, Suren Felekyan, Annemarie Greife, Xiaoyue Shang, Arthur Bister, Constanze Wiek, Claus A. M. Seidel, Cornelia Monzel

**Affiliations:** ^1^ Experimental Medical Physics, Heinrich-Heine University, Düsseldorf, Germany.; ^2^ Molecular Physical Chemistry, Heinrich-Heine University, Düsseldorf, Germany.; ^3^ Department of Otorhinolaryngology, Head & Neck Surgery, Heinrich-Heine University, Düsseldorf, Germany.

## Abstract

Unraveling the concentration-dependent spatiotemporal organization of receptors in the plasma membrane is crucial to understand cell signal initiation. A paradigm of this process is the oligomerization of CD95 during apoptosis signaling, with different oligomerization models being discussed. Here, we establish the molecular-sensitive approach cell lifetime Förster resonance energy transfer image spectroscopy to determine CD95 configurations in live cells. These data are corroborated by stimulated emission depletion microscopy, confocal photobleaching step analysis, and fluorescence correlation spectroscopy. We probed CD95 interactions for concentrations of ~10 to 1000 molecules per square micrometer, over nanoseconds to hours, and molecular to cellular scales. Quantitative benchmarking was achieved establishing high-fidelity monomer and dimer controls. While CD95 alone is primarily monomeric (~96%) and dimeric (4%), the addition of ligand induces oligomerization to dimers/trimers (~15%) leading to cell death. This study highlights molecular concentration effects and oligomerization dynamics. It reveals a minimal model, where small CD95 oligomers suffice to efficiently initiate signaling.

## Teaser

Advanced FRET image spectroscopy reveals a minimal model where ~15% CD95 receptors oligomerize for signal initiation.

## INTRODUCTION

Identifying the spatiotemporal organization and dynamical interactions of receptors in the plasma membrane is key to our understanding of cell signal initiation. So far, we know about the molecules participating in distinct signaling cascades; however, insights about their oligomerization states, assembly kinetics, and the role of molecular concentration during this process remain sparse (*1*). This is mostly due to a lack of suitable techniques and analyses routines to accurately quantify the photon-based molecular information in microscopy and to measure oligomerization dynamics.

**Fig. 1.**
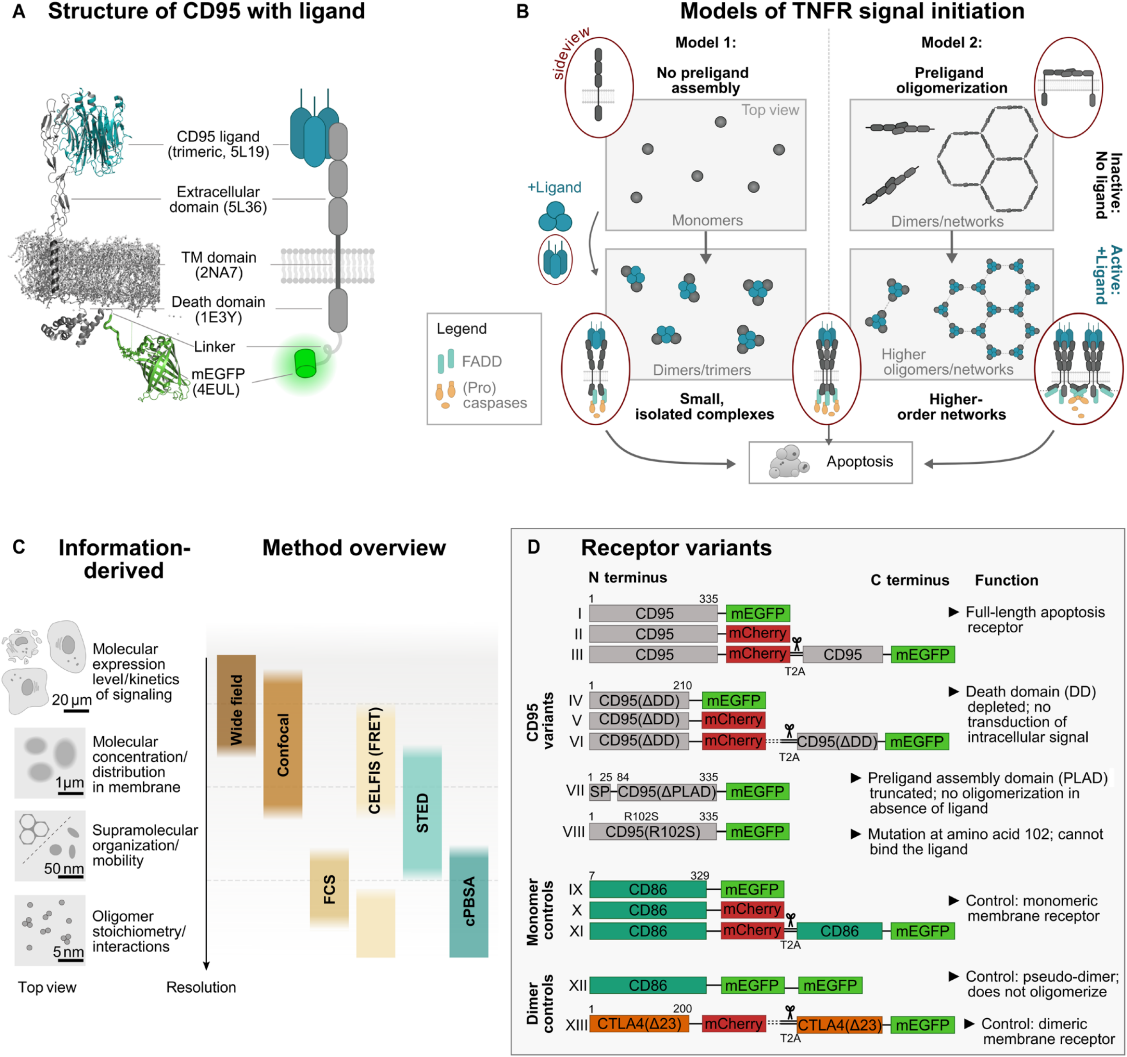
Probing CD95 signal initiation models with receptor variants over a broad range of molecular concentrations and in space and time. (**A**) Structure and cartoon of CD95 with genetically fused mEGFP and trimeric CD95L. Four-letter abbreviations are protein data bank IDs. For simplicity, only one of up to three CD95 receptors is shown together with CD95L. (**B**) Scheme of proposed TNFR signal initiation models. Model 1: Monomeric receptors bind trimeric ligands and form up to trimer-trimer receptor-ligand configurations. In the receptor activated state, the intracellular death domain (DD) recruits an adaptor molecule [Fas-associated death domain protein (FADD) in case of CD95]. A cascade of (pro)caspase activation follows (*74*) along with mitochondrial dysfunction (*75*) and protein cleavage, resulting in cell apoptosis (*76*). Model 2: Before activation, TNFRs form inactive dimers, which assemble into a supramolecular hexagonal lattice (units of ~24 nm in diameter depending on TNFR) (*12*). After ligand binding, the receptor dimers decouple and recruit FADD to the DDs. FADD may cross-link the DDs, from where the (pro)caspase cascade evolves as in model 1. (**C**) Overview of test strategy using (super-resolution) microscopy and multiparametric fluorescence spectroscopy techniques covering single molecule to cellular scales. (**D**) Scheme of engineered CD95 variants exhibiting different signaling competencies (I to VIII), monomer controls (IX to XI), and dimer controls (XII and XIII). Bicistronic plasmids are used for CELFIS, and monocistronic plasmids are used with all techniques. Numbers indicate the amino acid, and dashed lines indicate optional linkers. Gray panel indicates a methodological highlight.

A paradigm of signal initiation is given by the characteristic molecular organization proposed for tumor necrosis factor receptors (TNFRs), with the most prominent molecular configurations described below. The understanding of TNFR-induced signaling is important, as these receptors initiate signaling for cell proliferation, morphogenesis, and, most prominently, cell apoptosis (*2*–*4*). TNFRs are further targets of therapeutic approaches for various diseases, including cancer, autoimmunity, or infectious diseases (*5*, *6*). Of particular interest is the TNFR CD95 (Fas or TNFR6), as it is exclusively activated by the trimeric CD95 ligand (CD95L) (FasL, TNFL6, or CD178), thus providing high control over the stimulation of the receptor (Fig. 1A).

Two models of TNFR oligomerization are primarily discussed to explain the molecular mechanisms underlying signal initiation (Fig. 1B) (*7*–*9*): The first model proposes initially monomeric receptors, which, upon binding of the trimeric TNF ligand, recruit further receptors to form small signaling units of up to trimer-trimer receptor-ligand configurations. Features of this first model comprise (i) a direct signal transduction from the extracellular to the intracellular side, without the need for massive spatial molecular rearrangements, and (ii) its occurrence already at low molecular expression levels. A second model proposes TNFRs to form inactive dimers before their activation, which, in turn, assemble into a supramolecular honeycomb lattice, placing the receptors some ~12 nm apart (with exact values varying between TNFRs) (*10*–*13*). After TNF ligand binding and receptor activation, the intracellular receptor domain is cross-linked to reestablish the honeycomb lattice on the intracellular membrane side. Features of this second model are (i) a unique molecular complex permitting robust signal initiation and (ii) potential signal amplification by a factor of ~1.4 (*11*).

Here, we scrutinize these models, choosing CD95 as a TNFR example. So far, qualitative observations of CD95 oligomerization have been reported (*14*). What is hitherto missing is a quantification of oligomer type and number that are necessary to initiate the signaling along with monitoring the oligomerization dynamics in live cells over time and its concentration dependence. To address this need, we here establish the Förster resonance energy transfer (FRET)-based (*15*, *16*) cell lifetime FRET image spectroscopy (CELFIS). CELFIS is an advanced FRET-FLIM (fluorescence lifetime imaging) analysis, which measures CD95 intermolecular distances with 3- to 8-nm spatial resolution fully automatized and dynamically over the whole cell, along with CD95 surface concentrations. It determines receptor interactions with 1.6% fraction precision and enables to account for receptor proximity effects. To cover large ranges in concentration, time, and space, we follow a multiscale approach and use complementary state-of-the-art microscopy (Fig. 1C): Next to CELFIS, stimulated emission depletion (STED), polarization-resolved confocal photobleaching step analysis (cPBSA), and fluorescence correlation spectroscopy (FCS) are used to probe the dynamics and number of receptors over larger areas up to a diffraction-limited spot (~250 nm). Our strategy further comprises a small library of CD95 variants with different signal initiation competencies and high-fidelity monomer and dimer controls. In all cases, rigorous image analysis and benchmarking against control samples allowed us to identify concentration and photophysical-based effects and to accurately quantify CD95 oligomeric states. Thus, the regulation of CD95 before and during the signaling process is mapped, and a minimal model of CD95 signal initiation is derived. Notably, the presented approach will be suitable to quantitatively study other membrane receptors and their signal initiation in live cells. It will further provide important values to acutely model these processes (*17*, *18*). Examples include but are not limited to immune cell activation mediated via (i) receptors in the immunological synapse (*19*), (ii) chimeric antigen receptors (*20*–*22*), (iii) interferon-α/β receptor signaling (*23*), (iv) cell proliferation and differentiation via epidermal growth factor receptor (*24*), or (v) CXCR4 (*25*).

## RESULTS

### Engineered plasma membrane receptors for molecular quantification in super-resolution and multiparametric fluorescence microscopy

We have collected a small library of monomeric enhanced green fluorescent protein (mEGFP)–and monomeric red fluorescent protein (mCherry)–labeled CD95 variants with different competencies to recognize and transduce the signal initiated by CD95L (Fig. 1D). Next to monocistronic plasmids, we used bicistronic constructs, combining mCherry- and mEGFP-labeled proteins, to ensure homogeneous coexpression of donor and acceptor fluorophores during FRET measurements. To quantify receptor oligomerization states, we established high-fidelity monomer and dimer controls using mEGFP- or mCherry-labeled CD86 and cytotoxic T lymphocyte–associated protein 4 (CTLA4) membrane receptors (*26*), respectively. As described below, generating a pseudo-dimer control from CD86 with two genetically fused mEGFP was necessary to confirm the CTLA4 dimerization state. For further details on the design of the 13 plasmids, see Materials and Methods. Before measurements, correct integration of all receptors into the plasma membrane was verified using confocal microscopy (see fig. S1). Since CD95 activation was shown to depend on the presentation of CD95L [in solution, membrane-anchored or cross-linked (*27*)], we chose a CD95L that can enhance CD95 activation by cross-linking (see Materials and Methods).

### The efficiency of signal initiation relies on receptor expression levels and ligand concentrations

**Fig. 2.**
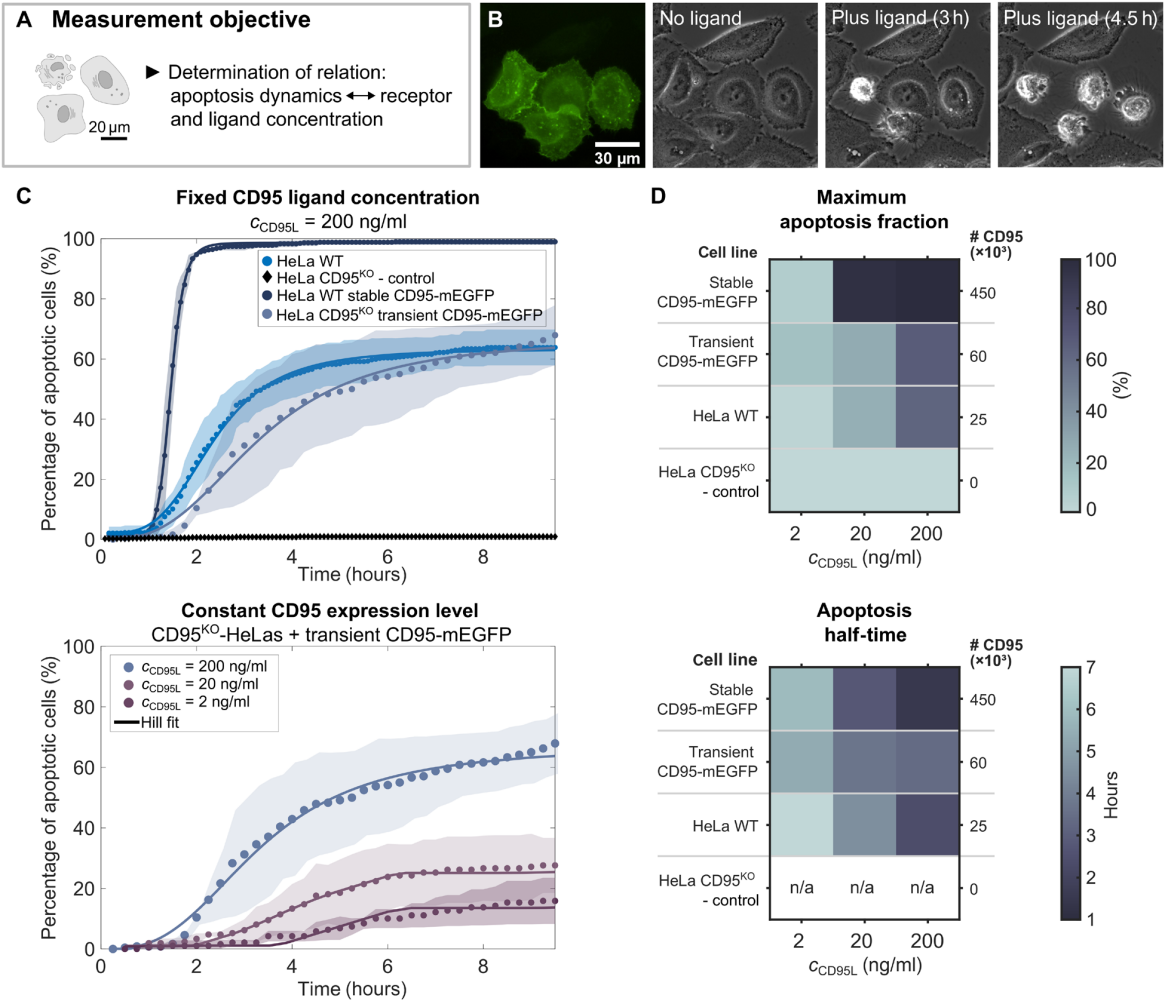
Apoptosis dynamics depend on molecular concentration levels. (**A**) Objective of time-lapse measurements. (**B**) mEGFP fluorescence and phase-contrast microscopy of HeLa CD95^KO^ cells transiently transfected with CD95-mEGFP before and after CD95L addition. Three and 4.5 hours after incubation with CD95L (200 ng/ml), apoptosis of transfected cells is observed. Nontransfected cells are unaffected by CD95L. (**C**) Percentage of apoptotic cells over time after CD95L addition. From a Hill equation fit (solid line, Eq. 1 in Materials and Methods), apoptosis dynamic parameters shown in (D) are derived. Top: Comparison of cell lines with different CD95 expression levels exposed to ligand concentration (200 ng/ml). Bottom: Comparison of HeLa CD95^KO^ transiently expressing CD95-mEGFP cell line exposed to ligand concentrations of *c*_CD95L_ = 2, 20, and 200 ng/ml. Data points show the weighted mean, and shaded area shows the SD of three independent measurements. *N* > 180 cells per sample. (**D**) Hill fit parameters of different cell lines and ligand concentrations, *c*_CD95L_. Top: Maximum apoptosis fraction. Bottom: Apoptosis half-time. n/a indicates data where no Hill fit was possible because of a low percentage of apoptotic cells. The CD95 expression level of HeLa wild-type (WT), HeLa CD95^KO^, and HeLa WT stably expressing CD95-mEGFP was determined with the QIFIKIT. CD95 expression levels after transient transfections were derived from quantitative STED analysis. For further details, see STED imaging and analysis in Materials and Methods.

We first examined CD95 signal initiation and transduction on the cellular level to quantify effects of ligand concentration and receptor density on the signaling dynamics and outcome (Fig. 2A). To this end, we recorded HeLa cell lines exhibiting different CD95 expression levels between 0 and 4.5 × 10^5^ receptors per cell, as quantified by flow cytometry. Cells were exposed to various ligand concentrations, and the dynamics of the cellular fate decision was monitored. Several hours after CD95L incubation, the cells showed typical apoptosis characteristics such as blebbing, followed by cell shrinkage (Fig. 2B). In all cases, the percentage of apoptosis events followed a sigmoidal progression. The initial onset just 1 hour after ligand addition indicated the minimal time the signal takes from its initiation until the eventual cell death. The predominant time interval of apoptosis events was between 1 and 5 hours after ligand addition, whereas the slowest signaling outcome was detected after 5 to 7 hours, depending on the experimental situation and in line with time scales, previously observed (*28*, *29*). The few apoptosis events recorded after this time were attributed to naturally occurring apoptosis and/or stress due to the long-time recording on the microscope. We observed a ligand-dependent efficiency of apoptosis induction from 3 to 99% apoptotic cells, when the ligand concentration was increased from 2 to 200 ng/ml.

Similarly, apoptosis initiation scaled with the number of receptors on the cell surface, where a complete knockout of CD95 (CD95^KO^) (0 receptors) led to no apoptosis, 2.5 × 10^4^ CD95 molecules per cell led to 60 to 75% apoptotic cells, and 4.5 × 10^5^ CD95 molecules per cell led to 99% apoptosis (Fig. 2, C and D). A fit of the Hill function (see Materials and Methods) yielded the time after which half of all apoptotic cells died. These half-times ranged from 1.5 to 8 hours and became shorter with higher CD95L concentration or receptor expression level (Fig. 2D). As a negative control, we used cells expressing CD95(ΔDD), i.e. CD95 with truncated DD, or CD95(R102S), where the latter exhibits a mutation at amino acid 102 (premature protein) and is suitable as control, which cannot bind the ligand (*30*). In both cases, less than 15% of apoptotic cells within 10 hours were observed, where the apoptosis was caused either naturally or additionally by transfection stress. Furthermore, CD95 lacking the preligand assembly domain (PLAD) [CD95(ΔPLAD)], which cannot dimerize in absence of the ligand, exhibited apoptosis dynamics slightly exceeding the negative controls, with up to 25% of apoptotic cells (see fig. S2).

By analyzing the apoptosis dynamics, characteristic time points of the signaling process were derived, which are important for subsequent measurements with CELFIS, cPBSA, FCS, or STED: (i) time points before signal initiation, (ii) directly after ligand addition, (iii) when most cells underwent apoptosis, and (iv) when all signaling events were finished. The apoptosis dynamics exhibited a strong correlation with ligand and receptor concentration, demonstrating that signal initiation is highly dependent on the absolute number of available ligand and receptor molecules. For this reason, particular attention was paid to ligand and receptor numbers during the following measurements.

### Ligand-induced signal initiation does not affect receptor mobility in the plasma membrane as revealed by live-cell FCS

Before single-molecule analyses of CD95 oligomeric states, we tested whether CD95 is sufficiently mobile and hence able to form (higher) oligomers using FCS (see Materials and Methods, figs. S3 and S4, and notes S1 and S2 for optimal FCS settings in live cells) (*31*). CD95 and CD95(ΔDD) samples revealed an average diffusion coefficient *D* = 0.23 ± 0.02 μm^2^/s, which is typical of individually diffusing membrane proteins (*32*, *33*). This value did not change considerably over 220 min in the presence or absence of CD95L and confirmed sustained CD95 mobility during the signaling process (fig. S3).

### CELFIS reveals ≥96% initially monomeric and ≤4% initially dimeric CD95 receptors that form small oligomers after ligand activation and a dynamic increase in the oligomer fraction up to 15% of all CD95 receptors

**Fig. 3.**
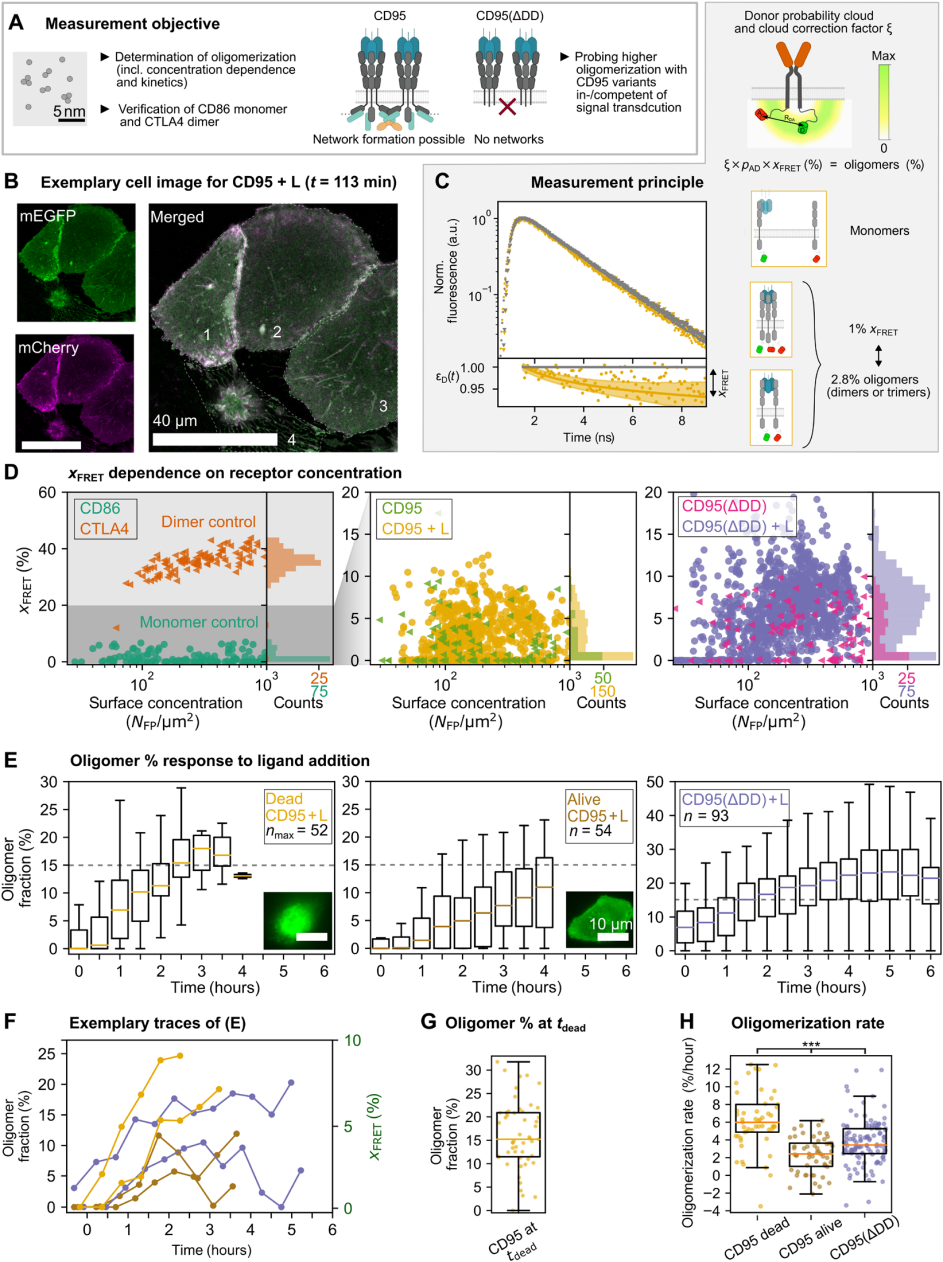
CELFIS quantifies CD95 oligomerization. (**A**) Measurement objectives. (**B**) Confocal fluorescence image of mEGFP- and mCherry-labeled CD95 in the cell membrane. Cells 1 to 3 are alive. Cell 4 underwent apoptosis. (**C**) Methodological approach. Left: Distribution of donor fluorescence lifetimes in absence (D0; gray) and presence (DA; yellow) of FRET. FRET-induced donor decay ε_D_(*t*) with fluorescence fraction (*x*_FRET_) in presence of the acceptor. Right: Conversion of 1% *x*_FRET_ into 2.8% oligomer fraction from theoretical considerations accounting for the probability of mature donor-acceptor pairs, *p*_AD_, and the fluorophore cloud correction, ξ. The conversion was also experimentally confirmed. a.u., arbitrary units. (**D**) *x*_FRET_ as a function of receptor surface density. About 3 % *x*_FRET_ values confirm the monomeric character of CD86, and nearly constant ~37 % *x*_FRET_ values confirm the dimeric nature of CTLA4. *x*_FRET_ of CD95 alone indicates primarily monomeric (≥96%) and some dimeric (≤4%) receptors. For CD95(ΔDD), ≥88% monomers and 12% dimers are found. After CD95L incubation, ≤21% of CD95 or CD95(ΔDD) receptors form oligomers. *N* > 108 cells; ≥4 independent experiments per condition. Note the adjusted *y* axis. (**E**) Dynamics of oligomerization after CD95L addition. Box plots of oligomer fraction calculated from *n*_(max)_ cells. The “max” in case of “dead CD95 + L” indicates the initial cell number, which decreases over time. Dashed line indicates 15% oligomer fraction from (G). (**F**) Exemplary evolution of the oligomer fraction in single cells. Legend as in (E). (**G**) Oligomer fraction right before apoptosis. (**H**) Oligomerization rate over ≤3 hours, depending on the apoptosis time point. Legend as in (E). Two-sided Mann-Whitney *U* test, ***P < 0.001.

To determine CD95 oligomeric states during the signal initiation process and to probe the effect of receptor surface concentrations, we used and advanced FRET image spectroscopy to probe molecular proximity before and after ligand addition (see Fig. 3) (*34*–*37*). To this end, we transfected cells to exhibit different surface concentrations of CD95 or CD95(ΔDD) and performed FRET measurements in absence and presence of the ligand. In addition, these FRET measurements establish the receptors CD86 as monomeric no-FRET and CTLA4 as a dimeric positive-FRET control. In all cases, bicistronic plasmids were used to ensure homogeneous donor and acceptor expression. To systematically tune the range of receptor surface concentrations, we titrated the amount of receptor DNA used for transfection against an empty vector, while keeping the total amount of DNA constant. We further derived the molecular brightness of the fluorophore and converted fluorescence intensities into surface densities [*N*_FP_/μm^2^] (see Materials and Methods). Figure 3B shows the localization of the CD95 receptor in live cells by confocal images of the lower cell membrane. The increased intensity at cell edges and cell-to-cell contacts confirms the primary integration of the receptor into the cell plasma membrane. Figure S1 shows similar images for CD95(ΔDD), CD86, and CTLA4.

For CELFIS, we evaluated changes in the donor fluorophore lifetime due to FRET. This occurs whenever an mEGFP donor–labeled receptor and a second receptor with an mCherry acceptor molecule are in close proximity due to binding (≤10 nm). In previous works, FRET measurements already demonstrated to be highly suitable to resolve protein interaction and oligomerization (*38*, *39*). To determine the average oligomerization state with great accuracy, we collected the data of receptors over the whole lower cell membrane and integrated all photons into one fluorescence decay per cell. Figure 3C illustrates the core principle of CELFIS: The fluorescence decay is measured in the FRET sample (i.e, the sample with a donor and an acceptor present, DA) and in the control sample, expressing the donor in absence of the acceptor (D0). Normalizing the DA fluorescence decay with respect to the average D0 decay yields the FRET-induced donor decay [ε_D_(*t*)] (*34*, *40*, *41*). The amplitude drop, *x*_FRET_, directly corresponds to the fraction of donors (i.e., receptors on the cell membrane) quenched by FRET (*34*). We then applied a pattern fit to the measured lifetime decay to obtain robust results (see Materials and Methods and Eqs. 3 to 11). From this, we determined the *x*_FRET_ value for each cell individually and studied its dependence on the receptor surface concentration, [*N*_FP_/μm^2^] (Fig. 3D).

At first, we measured the CD86 and CTLA4 controls and thereafter rated all CD95 samples against these controls. For CD86-expressing cells, we observed a low average *x*_FRET_ ~3 % with ∆*x*_FRET_ ± 3% over the whole concentration range from 30 up to 1000 receptors/μm^2^. These data show that CD86 is monomeric. Similarly, CELFIS data of CTLA4 were nearly constant (average value of *x*_FRET_ ~37 % with ∆*x*_FRET_ ± 7%) with deviations only at the lowest receptor concentrations, supporting the dimeric nature of this control. At concentrations of >1000 receptors/μm^2^, a systematic increase in FRET in all samples [CD86, CTLA4, CD95, and CD95(ΔDD), in absence and presence of the ligand] indicated the onset of proximity FRET (see fig. S5). Note that determining this concentration threshold is important for any type of FRET measurement to not misinterpret the FRET signal due to proximity effects. For this reason and since first proximity effects appear around 1000 receptors/μm^2^ (*42*), the FRET data were evaluated only up to this threshold. *x*_FRET_ values of CD95 and CD95(ΔDD) in absence of the ligand revealed primarily monomeric receptors (with *x*_FRET_ ≤ 3%). In case of CD95, 96% of all receptors were monomeric, and up to 4% of receptors were dimeric (with *x*_FRET_ > 3%). In case of CD95(ΔDD), 78% of receptors were monomeric, and up to 12% of dimeric receptors were found. Upon ligand addition, the value of *x*_FRET_ immediately increased by several percent [CD95 up to *x*_FRET_ ~12% with ∆*x*_FRET_ ± 6% and CD95(ΔDD) up to *x*_FRET_ ~20% with ∆*x*_FRET_ ± 10%]. The higher oligomerization fraction in case of CD95(ΔDD) may arise, since (i) the cells are not dying and so the receptors can interact with each other over longer time scales; (ii) in case of CD95, the receptor oligomerization stops once the cells signaling is initiated and the receptor gets internalized; and/or (iii) the DD truncated receptor misses a steric hindrance effect by the DD domain and can more easily interact via its extracellular or transmembrane domain. By comparison with the controls, *x*_FRET_ values of CD95 and CD95(ΔDD) suggested the formation of dimers and/or trimers (Fig. 3D), as any higher oligomer would yield a *x*_FRET_ value closer to the dimeric *x*_FRET_ ~37%. Last, we derived a relation between the measured *x*_FRET_ value and the oligomer fraction to convert FRET data into a molecularly relevant number: This was done theoretically and experimentally with near identical findings. For the theoretical value, a sample-specific maximum *x*_FRET, max_ for a purely (=100%) dimeric sample was calculated (see Fig. 3C and note S3). The theoretical value takes into account the following aspects: (i) the distance distribution between the two fluorescent proteins with long linkers (see linker list in table S1) (*40*), i.e., the positional distributions of mEGFP (=donor probability cloud; see Fig. 3C) and mCherry (omitted for clarity) inside and outside of the FRET range, for which the cloud correction factor ξ is used (see note S3); (ii) the abundance of hetero-FRET species, which is corrected for the no-FRET species (e.g., donor-donor dimers); and (iii) an estimated maturation efficiency of 80% for mEGFP (*43*, *44*)) (for mCherry, a 100% maturation efficiency is used since immature mCherry is known to still be capable to absorb a photon from the donor). For (ii) and (iii), the probability of hetero-FRET species, *p*_AD_, was statistically calculated (see note S3).

From ξ and *p*_AD_, the correction factors of 36 and 33% *x*_FRET, max_ were calculated for a 100% CTLA4 and CD95 dimer sample, respectively. The *x*_FRET, max_ for a purely (=100%) dimeric sample was also determined from experimentally fitting the *x*_FRET_ concentration dependency of CTLA4 in Fig. 3D, correcting for proximity effects. This yielded a *x*_FRET, max_ of 39.3 and 36% for CTLA4 and CD95, respectively, for a 100% dimer sample. Both approaches agree very well, wherefore the latter, experimental determination was used to convert 36 % *x*_FRET, max_ of 100% CD95 oligomers into 1% *x*_FRET_ corresponding to ~2.8% CD95 oligomers. The calculation for CTLA4 was analogous.

Equipped with these tools, we then converted *x*_FRET_ into the percentage of oligomers and probed the oligomerization state over time until the point of apoptosis. Here, we recorded FRET data up to 6 hours after ligand addition by repeated measurements of the same cells. Cells expressing the full-length CD95 were classified according to apoptosis or no apoptosis events occurred within 4 hours after CD95L addition (Fig. 3E). For cells that underwent apoptosis, the oligomer fraction (determined from the experimentally derived conversion factor of 1% *x*_FRET_ corresponding to ~2.8% CD95 oligomers, see previous paragraph) started close to zero and increased quickly up to an 18% median value, whereas cells that did not show apoptosis exhibited a slower oligomer formation, reaching a ~12% median after 4 hours. CD95(ΔDD)-expressing cells, where downstream signaling was suppressed, showed a slightly higher initial oligomer fraction and reached a population equilibrium of 22% median after ~4 hours. In individual cell traces, rising and/or falling oligomer fractions were detected (Fig. 3F), representing transient CD95 dimerization or binding/unbinding kinetics of CD95 to CD95L. As a measure of CD95 oligomerization needed to initiate apoptosis, the oligomerization fraction just before apoptotic blebbing and shrinkage was determined, amounting to the interquartile range of ~11 to 21% with a median value of 15% (Fig. 3G). Last, we calculated the oligomerization rate from the oligomer fraction change per time interval, which was faster in case of CD95-transfected cells that died (6% oligomers/hour) compared to CD95- or CD95(ΔDD)-transfected cells that stayed alive (with 2.3 and 3.3% oligomers/hour respectively; Fig. 3H).

Overall, our FRET results demonstrate that signal initiation requires (additional) oligomerization after ligand addition and oligomers form within 2 to 3 hours over the whole membrane. These oligomers can develop even stronger in absence of a death domain, indicating that CD95 oligomerization may be mediated simply via ligand binding or by the transmembrane domain when the receptor is in the activated state, as previously suggested (*45*). Albeit CD95L was incubated in excess to the number of receptors, only about ~15% of receptors in the form of dimer or trimer oligomers are necessary to trigger efficient signaling. The fact that only ~15% of receptors are activated suggests that some of the CD95 receptors are blocked by interactions with other receptors on the membrane.

### cPBSA reveals small receptor stoichiometries and only weak molecular crowding effects

**Fig. 4.**
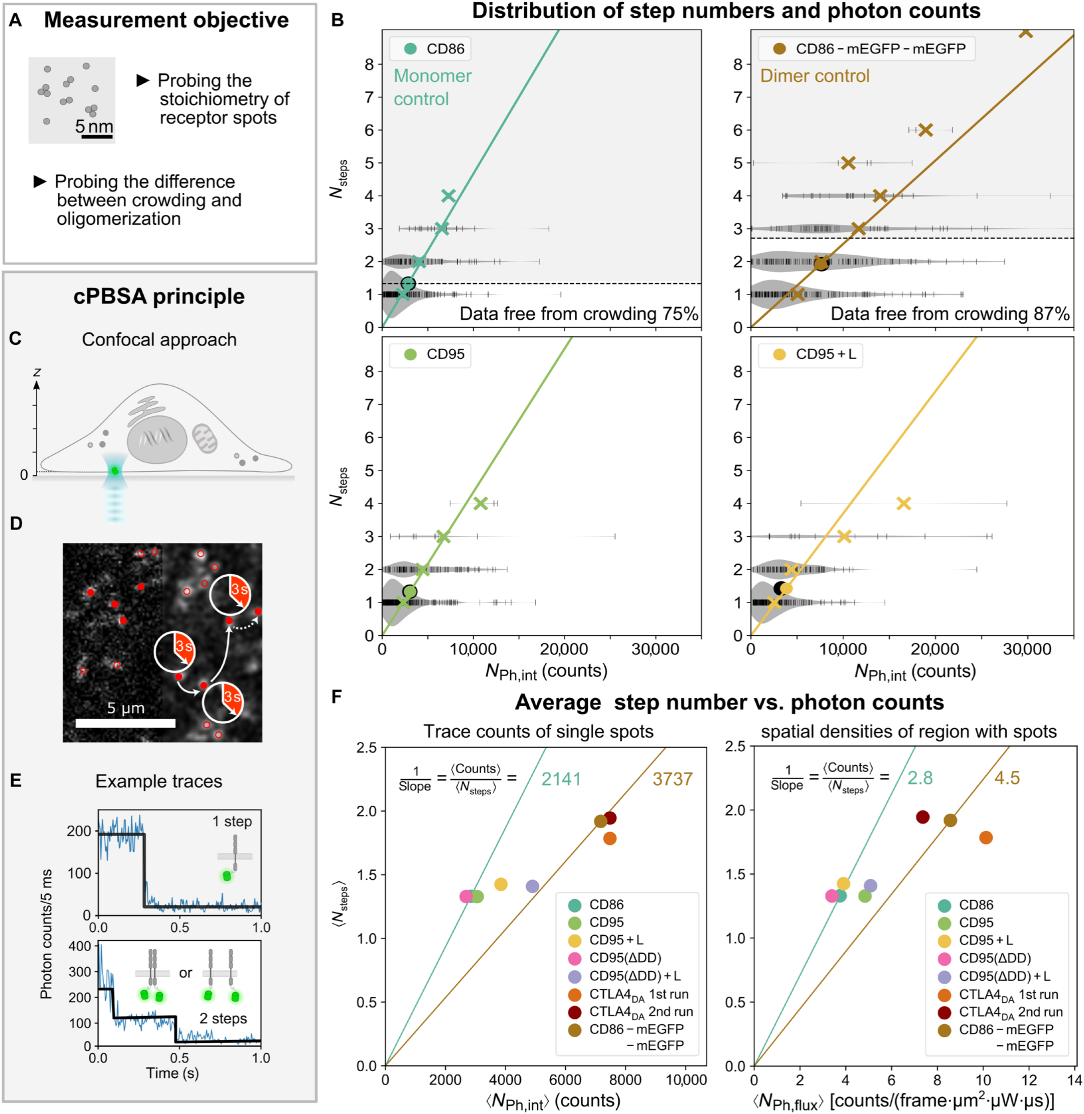
cPBSA reveals the stoichiometry of CD95. (**A**) Measurement objective. (**B**) Distribution of step numbers *N*_steps_ versus integrated photon counts *N*_Ph, int_ from all traces. *N*_steps_ values were derived from the KV fit, and *N*_Ph, int_ values were from integrating all photons of a bleaching trace. Lines are orthogonal regressions to mean total trace counts (crosses) weighted by the number of data points (black bars) of each *N*_*s*teps_. Black dots indicate the mean values of raw data, and colored dots indicate the mean values as a result of orthogonal regression. Dashed lines separate data points exhibiting crowding (higher *N*_steps_) from those without crowding (lower *N*_steps_). See text for details. (**C**) Method advancement of cPBSA. The confocal approach enables local trace analysis with minimal sample bleaching at arbitrary sample positions. (**D**) cPBSA spot detection algorithm: Confocal overview image (left half) is smoothed with Gaussian filter of 1 pixel sigma (right half). Bleaching traces are recorded for diffraction-limited spots with maxima of >4 photons (red circles) and with no neighbors (red dots). (**E**) Top: Exemplary trace of a monomer. Bottom: Exemplary trace of either a dimer or two monomers in one spot (crowding). Black lines indicate the KV fit. (**F**) Left: Average values of step number, 〈*N*_steps_〉, and integrated photon counts from single traces, 〈*N*_Ph, int_〉. Right: Average values of step number, 〈*N*_steps_〉, and photons from spatial flux densities, 〈*N*_Ph, flux_〉. Spatial flux densities correspond to photon counts in spot vicinities before bleaching. Lines indicate the average photon counts per step, exemplary for CD86 and CD86-mEGFP-mEGFP. See notes S4 and S5 for details.

Since CELFIS cannot determine exact CD95 stoichiometries in resolution-limited spots, we used PBSA. Here, the number of receptors is derived from steps occurring in the bleaching trace of fluorescent spots. In addition, cPBSA allows us to determine how crowding due to concentration variations of the receptors on the membrane surface influence our oligomerization quantification. Last, we can compare the oligomerization characteristics obtained by cPBSA (analyzing confocal spots) and CELFIS (averaging over the whole cell) (Fig. 4, A and B). In the past, PBSA was used to measure in vitro samples with photostable organic fluorophore labeling to determine the number of membrane bound proteins (*43*), the degree of quantum dot labeling (*46*), or the number of fluorescent labels on DNA origami (*47*), among others. To apply PBSA to CD95, we advanced the technique to be compatible with widely available confocal microscopes, to record data without bleaching large areas of the cell, and to use it with genetically encoded fluorescent labels. We further introduce how cPBSA can be used to determine a molecular crowding factor.

cPBSA was realized by a fast overview scan of the cell’s lower membrane to identify receptor locations, followed by recording the bleaching trace from diffraction-limited spots (Fig. 4, C to E, Materials and Methods, and fig. S6). The number of bleaching steps per trace, *N*_steps_, was determined using the well-known Kalafut-Visscher (KV) algorithm (*47*, *48*). Considering CD86, CD95, and CD95(ΔDD), a majority of *N*_steps_ = 1 or 2 were detected, with few higher values and a maximum number of bleaching steps, *N*_steps, max_ = 5. After ligand addition, *N*_steps, max_ did not increase, but the overall distribution slightly shifted to higher values. Thus, after CD95L addition, some oligomerization takes place, yet with receptor stoichiometries *N*_steps, max_≤ 5 remaining well below the stoichiometry of a hexagon. Moreover, only ≤1% of measurements return a value of 4, 5 to 6% return a value of 3, and about 22% return a value of 2 (see Fig. 4B). Considering the large detection volume and that no correction for crowding are applied to these values, it is clear that oligomers larger than trimers can be neglected, and the dimer fraction is significantly larger than the trimer fraction.

The dimer controls, CD86-mEGFP-mEGFP and CTLA4_DA_, exhibited a large fraction of *N*_steps_ = 1, 2, or 3 and continuously decreasing fraction of *N*_steps_ = 4, 5, or 6 up to *N*_steps, max_ = 9 (Fig. 4B and note S4 [figs. S14 to S16]). Dimer controls, hence, exhibit a substantial increase in *N*_steps_ as expected. *N*_steps, max_ = 9 further confirms that higher receptor numbers within diffraction-limited spots are generally detectable. Since PBSA is sensitive to changes in laser powers, molecular brightness, or minimal step sizes (see fig. S7, A and B, and table S2), we also evaluated the sum of photon counts per trace before photobleaching (*49*), *N*_Ph, int_ (=total trace count; Fig. 4, B and F, and note S4). This additional readout complements the conventional PBSA analysis and enables to plot *N*_Ph, int_ against *N*_steps_, where the mean of the total trace count for each *N*_steps_ (crosses in Fig. 4B) should follow a near linear relation. Applying an orthogonal regression, weighted by the number of data points (see lines in Fig. 4B and note S4 [figs. S14 to S16]), results in similar slopes for CD86, CD95, and CD95(ΔDD) samples before ligand addition, indicating similar overall behavior. Accordingly, dimer samples exhibited similar slopes as well. The slope continuously decreases for samples with higher oligomers and allows to determine a sample specific “counts per step” value (corresponding to the mean number of survived excitation cycles).

While the above analysis already excludes the existence of hexagonal or other oligomers with large receptor stoichiometries (since *N*_steps, max_≤ 5), we also used these data to introduce an approach to determining molecular crowding effects: First, we calculated the average step number, 〈*N*_steps_〉, and average photon counts, 〈*N*_Ph, int_〉, for each sample (dots in Fig. 4, B and F). To understand the obtained values, it has to be noted that molecular crowding gives rise to an overestimation of 〈*N*_steps_〉, whereas insufficient fluorophore maturation effects result in an underestimation of 〈*N*_steps_〉. For example, in case of CD86 and CD86-mEGFP-mEGFP, the 〈*N*_steps_〉 = 1 and 2 are expected for a perfect monomer and pseudo-dimer sample, respectively. Instead, 〈*N*_steps_〉= 1.33 is measured for the monomer sample, where the increase solely arises from molecular crowding (since an immature, invisible mEGFP cannot be detected). In case of the pseudo-dimer, 〈*N*_steps_〉= 1.92 is measured, where molecular maturation and crowding effects contribute. The variation in the step size (error of the population distribution) amounted to the expected ∆*N*_steps_ ∼ 0.5 to 1.0 (see note S4). Since the mEGFP maturation efficiency of ≲80% (*43*, *44*) is well known, 80% was used as a correction factor in the analysis. From CD86 and CD86-mEGFP-mEGFP, an average crowding factor of 〈*k*_crowd_〉 = 1.24 was determined, which quantifies the deviation from ideal behavior. This crowding factor is a value, which is generally inaccessible with other techniques (see Fig. 4B and note S5). We further calculated the fraction of receptors, which would not be affected by crowding *f*_no crowd_, yielding 75% for CD86 and 87% for CD86-mEGFP-mEGFP. Although we verified that all samples exhibited similar receptor expression levels (see fig. S7), the crowding parameters 〈*k*_crowd_〉, *f*_no crowd_(CD86),and *f*_no crowd_(CD86-mEGFP-mEGFP) varied within the sample population by up to 34, 60, and 77%, respectively (see note S5).

Figure 4F summarizes the average sample behavior, with 〈*N*_steps_〉 increasing from 1.33, in the case of CD86, CD95, and CD95(ΔDD), to 1.42, in the case of CD95 (+7%) and CD95(ΔDD) (+6%) after ligand addition. CD86-mEGFP-mEGFP and CTLA4_DA_ were significantly higher than all other measurements (*P* < 0.001) with 〈*N*_steps_〉 of 1.92 and 1.78, respectively. In case of CD86, CD95, and CD95(ΔDD), the near identical slope corresponds to an average of 2141 photon counts per step. After ligand addition, the slope increases for CD95 and CD95(ΔDD) up to the dimer control with a value of 3737 photon counts per step. When spatial flux densities of photon counts were used instead of summing the trace counts during bleaching, a very similar result was found (Fig. 4F, right, and note S4 for the calculation of spatial flux densities).

In conclusion, cPBSA revealed receptor stoichiometries well below the proposed hexagon, albeit higher receptor numbers (e.g., *N*_steps_ = 9) were generally detectable. Moreover, comparing spatial density and photon count cPBSA analysis of monomeric CD86 with CD95 and CD95(ΔDD) in their inactive state, a great similarity is observed. Hence, we can independently verify the primary monomeric characteristics of the CD95 variants. After ligand addition, the slope decreases toward the value measured for dimer samples (CD86-mEGFP-mEGFP). This indicates that several percentages of CD95 receptors form small oligomers (≤5 receptors; primarily dimers and trimers). The oligomerization characteristics observed by CELFIS (Fig. 3, D and E) and cPBSA (Fig. 4, B and F) agree very well so that the ~20% crowding or other effects give rise to only a minor distortion.

### STED confirms randomly distributed spots of small CD95 oligomers and no large CD95 networks over the cell plasma membrane

**Fig. 5.**
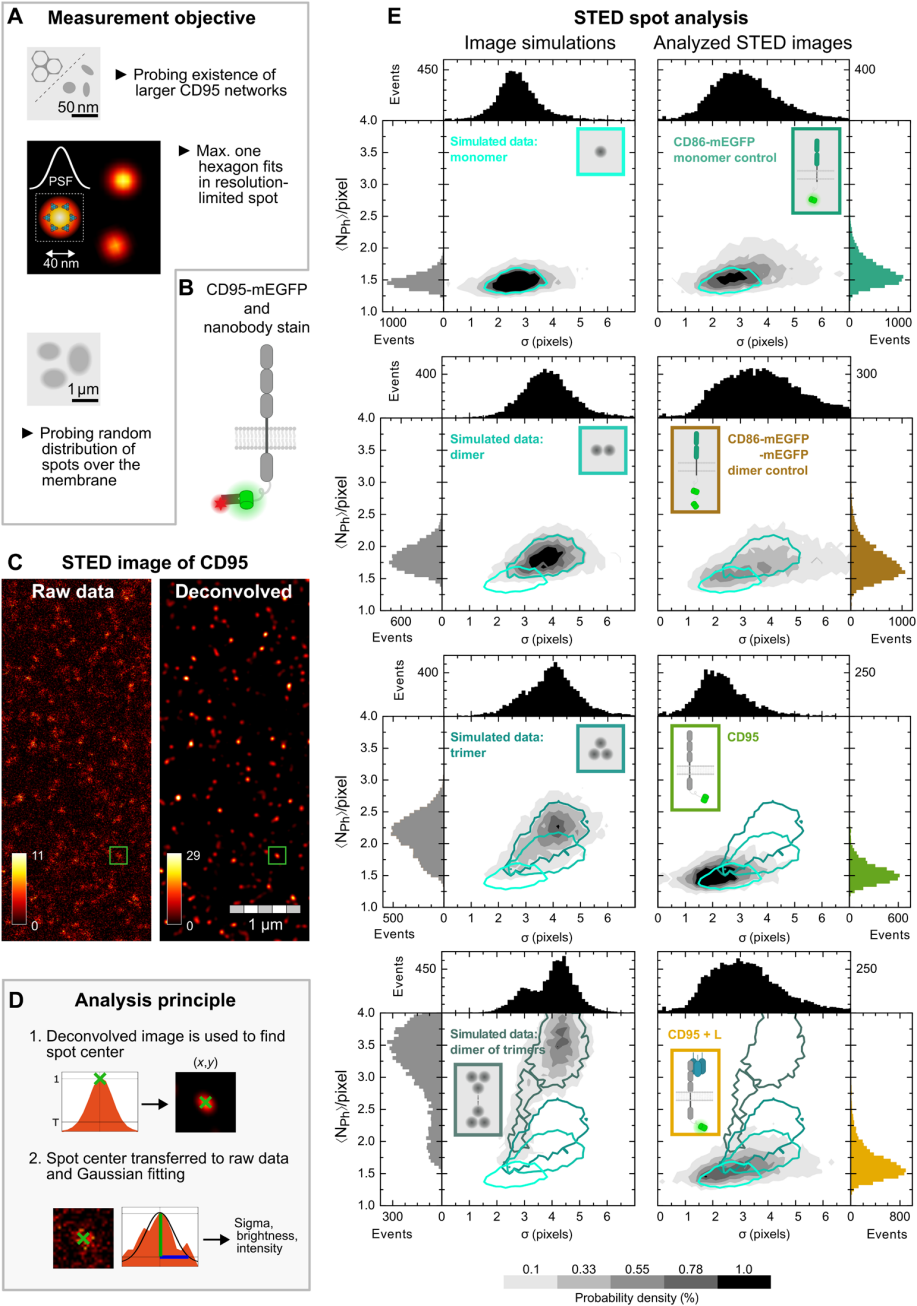
Quantitative STED imaging reveals randomly distributed CD95 spots and small oligomer formation. (**A**) Objective of STED measurements. (**B**) Schematic representation of CD95-mEGFP with GFP-nanobody Atto647N labeling. (**C**) Exemplary STED image (left) of HeLa CD95^KO^ membrane transiently transfected with CD95-mEGFP and deconvolved image (right) using Huygens Professional software (see STED imaging and analysis in Materials and Methods). On average, 20 spots/μm^2^ were detected. Green box indicates threshold-based detected spot analyzed in (D). (**D**) Gray panel illustrates methodological approach: Spot centers from deconvolved images are registered and superposed on raw data for Gaussian fitting (see Materials and Methods). From the fit, the SD σ, the brightness, and the average number of photons 〈*N*_Ph_〉 per pixel are derived. (**E**) 2D probability density representation of 〈*N*_Ph_〉 per pixel and σ values derived from individual spot analysis. Frequency histograms of 〈*N*_Ph_〉 per pixel and σ are depicted on the side and top of each graph. Left column: Simulation of monomer receptors up to dimer of trimer receptors per spot including their random distribution on the membrane surface. Simulation parameters (brightness, σ, and crowding factor; for details, see Materials and Methods and table S3) were adjusted to match the measured CD86 and CD86-mEGFP-mEGFP distributions precisely. Parameters of all oligomer simulations were kept constant. Right column: Measured 〈*N*_Ph_〉 per pixel and σ for monomer, pseudo-dimer control, and CD95 before and after CD95L incubation (for other receptors, see fig. S9). From each simulation, isolines enclosing 95% of data points are calculated and depicted in different panels for data comparison. *N* > 5000 objects analyzed per sample.

Last, we used STED nanoscopy, with its 40-nm full width at half-maximum resolution, as ultimate tool to probe the actual size of potential oligomers. So far, maximally five [CD86, CD95, and CD95(ΔDD)] or nine (CD86-mEGFP-mEGFP and CTLA4_DA_) molecules were detected by cPBSA in diffraction-limited confocal spots. With STED nanoscopy, we characterized the type of small oligomers by measuring the CD95 distribution over the membrane surface. For a quantitative analysis of the assemblies, we compared the size and brightness of the spots in our experimental STED images with simulations for monomers and for particular oligomers (Fig. 5A).

To this end, transfected HeLa cells were fixed 2 hours after ligand addition, when the signaling was initiated in most cells. CD95-mEGFP was stained with an excess of GFP-nanobody Atto647N, and the membrane surface was scanned with STED (Fig. 5, B and C). STED images revealed a distribution of CD95 in characteristic spots, which were randomly distributed over the membrane surface. This was shown for all samples in absence and presence of the ligand by calculating the pair correlation function *g*(*r*) of measured and simulated spot centers (see fig. S8). Spot analysis was performed in two steps: (i) Individual spots were analyzed using time gating (to minimize scatter) with maximum likelihood estimator–based deconvolution for spot center determination; and (ii) the selected spots were fitted by a Gaussian to determine the SD of spot sizes, σ, and the average photon number 〈*N*_Ph_〉 per pixel as a measure for the brightness (Fig. 5D and see Materials and Methods). For quantitative image analysis, we simulated randomly distributed spots of pure monomer, dimer, trimer, or dimer of trimers. To ensure that simulations represent our data correctly, the parameters 〈*N*_Ph_〉, σ, fluctuations in spot brightness, and crowding effects used in the simulation were matched with the experimental data of monomer and dimer controls (see Materials and Methods, figs. S9 and S10, and table S3). In these simulations, we observed pronounced changes in the brightness along with slight changes in the SD of spot sizes, σ (Fig. 5E, left column, and fig. S9). All probability density distributions of experimental data [CD95 and CD95(ΔDD), with and without ligand] reveal high overlap with simulated distributions of monomeric, dimeric, up to trimeric receptors. Distributions of higher oligomers, corresponding to higher 〈*N*_Ph_〉 per pixel values, are not present in any of the measured samples (except for a tiny overlap around 〈*N*_Ph_〉~2). Hence, these data corroborate and specify our CELFIS and cPBSA data, showing that primarily monomers and few dimers are present before ligand binding, whereas after ligand binding, receptor dimers or trimers exist in higher number. More precisely, the two-dimensional (2D) probability density representations in case of CD86 and CD95 before ligand addition revealed distributions with a median σ around 2 to 2.5 pixels and ~1.55 〈*N*_Ph_〉 per pixel, which confirms the primarily monomeric character. For CD86-mEGFP-mEGFP, CTLA4, and CD95 after ligand addition, distributions with a larger median σ of 3 to 3.5 pixels and higher ~1.75 〈*N*_Ph_〉 per pixel were obtained, corresponding to values that are generated by dimers (Fig. 5E and fig. S9). Analogous to the monomer and dimer controls, in nearly all other cases, a broad distribution of σ values and a narrow distribution of brightness values exist. The broadening of these distribution is enhanced because of the following contributions: (i) local concentration fluctuations of receptors on the membrane surface, (ii) limited maturation of fluorescent proteins [e.g., ≲80% for mEGFP (*43*, *44*, *50*)], (iii) a preferred fluorophore orientation near the membrane, or (iv) limitations in staining efficiency. We performed extensive controls to judge the impact of these effects on our images. Case i can be readily estimated from the monomer control, where σ exhibits a substantial distribution. To ensure similar surface concentration fluctuations between samples, we used a comparable receptor expression level (fig. S11). Cases ii and iv lead to a reduction of the brightness shift naturally occurring between the oligomer states; however, all oligomer states would be similarly affected. Last, to test whether fluorophore orientation plays a role (case iii), we analyzed the polarization-resolved fluorescence and found that anisotropy values were homogeneously distributed between 0 and 1 (fig. S12). Hence, fluorescent protein orientations are randomly distributed and contribute to the observed brightness variation.

In conclusion, the quantitative spot analysis of STED data together with simulations confirms that higher oligomers/networks of receptors beyond dimers/trimers do not exist in the studied cell systems. These analyses further highlight the use of high-fidelity monomer or dimer controls to generate accurate simulations of receptor distributions and to account for effects i to iv, which occur in every biological membrane sample.

## DISCUSSION

In previous studies, TNFRs (including CD95) were reported to appear as a mixture of monomers, dimers, or trimers in the absence of a stimulus (*9*, *51*). For CD95 without ligand, our measurements in live cells confirm the primarily monomer and very weak dimer oligomerization character of the receptor from low (physiological) to high concentrations. The exclusive monomeric/dimeric character of a TNFR before ligand addition was also reported by molecular-sensitive imaging of the cell plasma membrane (*29*, *51*). However, when a highly different physical and molecular environment compared to our situation was used, higher oligomeric states, such as preligand hexagonal networks of CD95 or oligomeric structures of pentagonal or hexagonal shape, have been reported. In these cases, molecular concentration levels were typically very high, in the range of ~0.5 mg of protein/ml (*52*) or ~100 μM (*10*) or where receptors were purified or reconstituted in synthetic bicelle membranes (*9*, *52*–*54*).

After ligand addition, we find the receptors oligomerizing to dimers/trimers. We correct for molecular proximity effects, which reduce the otherwise overestimated receptor stoichiometry. This is in line with other studies reporting CD95 and other TNFRs to be trimeric, without correcting for any proximity effects. For example, molecular-sensitive techniques, such as crystallography, single-molecule localization microscopy, and biochemical receptor cross-linking studies favor the trimeric state (*7*, *8*, *51*, *55*, *56*). In vitro studies of purified TNF-related apoptosis-inducing ligand coupling to death receptors 4 and 5, report stoichiometric changes of the protein:ligand complex from monomer:trimer to trimer:trimer configuration, when molecular concentrations were markedly changed from 1 nM to 10 μM (*57*). A general observation of molecular clustering was also reported using wide-field fluorescence microscopy, albeit without quantifying molecular numbers or interactions (*28*, *51*, *58*). All studies report about oligomerization after ligand addition, which we here confirm to be the decisive factor for apoptosis signal initiation (Fig. 3E).

The oligomerization of TNFRs is currently discussed to originate from one of the following molecular interactions: (i) the coupling of up to three receptors to the trimeric ligand, without the need for their direct intermolecular contact; (ii) interactions between CD95 transmembrane domains after ligand activation (*45*); or (iii) intracellular cross-linking of two CD95 DDs via Fas-associated death domain protein (FADD) (*10*, *59*). Cases i and ii would result in close packing of CD95 receptors with few-nanometer intermolecular spacing around the ligand up to a trimer-trimer configuration (*60*). Case iii suggests that cross-linking of two DDs via FADD occurs by which higher oligomeric structures of hexagons could develop (*10*–*13*), albeit the DD-FADD interaction was reported to be weak (*10*). Our data support cases i and/or ii since DD truncated receptors exhibited near identical oligomerization behavior compared to full-length CD95. This type and degree of oligomerization are hence sufficient to efficiently initiate the signaling (Fig. 3, D and E, and fig. S9). Case iii either may not exist or may provide additional stabilization to the oligomers via DD-DD cross-linking. CD95 dimer/trimer formation is hence primarily mediated via direct ligand (i) or ligand-induced transmembrane (ii) interactions.

No substantial changes in molecular oligomerization states were detected over a large interval of receptor surface concentrations ranging from physiological to enforced higher expression levels (10 to 1000 receptors/μm^2^ in live cells). Notably, even at the overexpression level, higher-order oligomers are not observed in live cells. However, there is a significant change in signaling dynamics and the percentage of apoptosis events depending on the absolute ligand and receptor number. Here, as well as in previous studies (*28*, *61*), increasing CD95 or CD95L concentrations led to a significant acceleration of downstream signaling and systematic increase in apoptosis events. As a result, the absolute number of activated receptors appears to play an important role in apoptosis signal initiation.

**Fig. 6.**
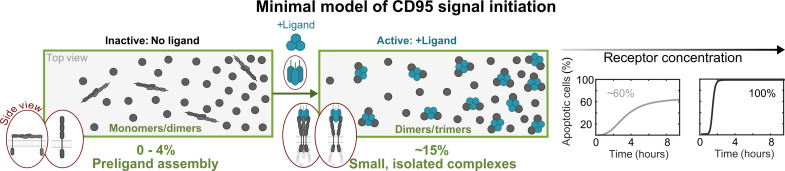
Schematic illustration of the minimal CD95 signal initiation model derived in this study. Primarily monomeric CD95 with up to 4% receptors exhibiting preligand assembly reside on the cell membrane. After ligand binding, ~15% of the receptors form small, isolated complexes with CD95 dimers/trimers. Increasing levels of receptor expression (membranes) do not lead to higher oligomerization states. Instead, the absolute number of activated CD95 dimers/trimers increases the percentage and dynamics of apoptotic cells.

To our best knowledge, this study presents the minimal model for CD95 signal initiation, where receptors are initially monomeric (≥96%) and dimeric (≤4%) and randomly distributed over the cell plasma membrane. After ligand addition, CD95 oligomerizes to dimers and trimers within the first 2 to 3 hours with a final fraction of 15% receptors inducing apoptosis signaling in live cells efficiently. A larger fraction of oligomers is not observed, potentially since some CD95 molecules are blocked by interactions with other membrane proteins. Only for CD95(ΔDD), all techniques observed a higher dimer/trimer fraction, since CD95(ΔDD) can oligomerize further on long time scales, whereas CD95 may already be internalized since the DD might introduce some steric hindrance, which is absent for CD95(ΔDD). Notably, our results do not exclude the existence of proposed higher-order oligomeric states but confirm that they are not necessary for initiating the signal (Fig. 6).

For these measurements, CELFIS is introduced to determine oligomerization states over the whole cell and during the signaling process. The high precision and sensitivity were obtained by workflow automation, measuring and analyzing a large number of cells. For CD95 stoichiometries in fluorescent spots, an extended approach to cPBSA is introduced, where mEGFP fluorescence labeling and confocal instead of total internal reflection fluorescence imaging are established to make cPBSA applicable to common biological samples and more flexible in space. Together with the spot analysis of STED data and FCS, this study highlights the importance of molecular oligomerization determination over a high dynamic range of microseconds to hours, nanometers to 100-μm scales, and 1 to 10^4^ molecules/μm^2^. Moreover, it establishes high-fidelity monomer and dimer controls, which are necessary to distinguish true oligomerization from molecular crowding effects.

In summary, this work elucidates the mechanisms underlying CD95 signal initiation and reports the minimal type and number of CD95 oligomers developing during signal initiation. To this end, a generic strategy for molecular oligomerization quantification in live cells is introduced, which is generally applicable to the study of cell signal initiation processes.

## MATERIALS AND METHODS

### Experimental design

#### Plasmids, molecular cloning, and stable cell lines

For all measurements with transient transfections, a stable HeLa cell line with CD95^KO^ was used (HeLa CD95^KO^). It was generated using CRISPR-Cas9 (*62*), the guide RNA was CATCTGGACCCTCCTACCTC (*29*). For apoptosis dynamics, we additionally used HeLa wild-type (WT) cells (from American Type Culture Collection, Manassas, VA, USA) and a stable, overexpressing cell line HeLa CD95-mEGFP, expressing CD95-mEGFP on top of endogenous CD95 (*29*). HeLa CD95^KO^ and HeLa stable CD95-mEGFP cell lines were provided from J. Beaudouin (formerly Institut de Biologie Structurale, Grenoble).

For CD95 constructs, four different sequences were used: the full-length protein CD95 (amino acids 1 to 335), a death domain truncated version CD95(ΔDD), CD95(R102S), and CD95(ΔPLAD). For CD95(ΔDD), amino acids 211 to 335 were truncated. CD95(ΔDD) is not capable to transduce the intracellular signal and is hence ideally suited for long-time observations after ligand incubation and to probe oligomerization mediated by the extracellular and transmembrane domain of CD95. CD95(ΔPLAD) is the PLAD-depleted variant, missing amino acids 26 to 83. It may be used to detect preoligomerization based on transmembrane and intracellular interactions. All amino acid numbers refer to the premature protein sequence (including signaling peptide). CD95(R102S) exhibits a mutation at amino acid 102 (premature protein) and is suitable as control that cannot bind the ligand.

As monomer control plasmid, the full-length sequence of CD86 (*26*) was used. For the dimer control, CTLA4, the last 23 amino acids of the sequence were removed to reduce receptor internalization and to concentrate it at the plasma membrane (*63*). As a second (pseudo-) dimer control, CD86 was fused to two consecutive mEGFPs. The UniProtKBs of CD95, CTLA4, and CD86 are P25445, P16410 and P42081-3, respectively.

All plasmids except CD86-mEGFP-mEGFP were also provided from J. Beaudouin (formerly IBS, Grenoble). These plasmids were designed by fusing the coding sequences of the protein’s C terminus (intracellularly) via a linker to mEGFP (called D0/donor only) or mCherry in the pIRESpuro2 vector (Clontech) (for more linker details, see table S1) (*29*). Besides these monocistronic constructs for CD86, CD95, CD95(ΔDD), and CTLA, we additionally used bicistronic plasmids combining the mCherry and mEGFP versions of a protein into one plasmid. This is to ensure homogeneous coexpression of donor and acceptor (called DA/donor-acceptor) in FRET measurements, albeit the first transcribed mCherry-labeled protein turns out to be a factor 2× more abundant. The bicistronic constructs with a 2A peptide use the sequence EGRGSLLTCGDVEENPGP as linker between the two proteins (*29*). Note that only CTLA4_DA_ was used and not CTLA4_D0_, as the latter did not localize well to the plasma membrane. The CD86-mEGFP-mEGFP pseudo-dimer control was synthesized using a cloning service (BioCat GmbH Heidelberg, Germany) by fusing two linked mEGFP proteins C-terminally to the CD86 full-length sequence of CD86 in a pcDNA3.1(+) vector (BioCat GmbH).

#### Cell culture, transfections, and ligand incubation

All cells were maintained in culture medium, consisting of Dulbecco’s modified Eagle’s medium) + GlutaMAX (31966021, Gibco, Life Technologies Inc., Carlsbad, CA, USA) containing 10% fetal bovine serum (10500064, Gibco) and 1% penicillin/streptomycin solution (P0781, Sigma-Aldrich, Merck KGaA, Darmstadt, Germany), in an environment with 5% CO_2_ (v/v) at 37°C.

For all live-cell measurements and cPBSA, cells were trypsinized (T3924, Sigma-Aldrich) and seeded in an eight-well glass-bottom slide (#80827, ibidi GmbH, Gräfelfing, Germany) with a density of 3 × 10^4^ to 5 × 10^4^ cells per well. For STED immunostaining, 100 × 10^4^ to 150 × 10^4^ cells were seeded on a sterile glass coverslip (13 mm in diameter, no. 1.5H, 0117530, Paul Marienfeld GmbH & Co. KG, Lauda-Königshofen, Germany).

Transfections were performed using ViaFect transfection reagent (#E4981, Promega Corp., Madison, WI, USA) at a cell density of 60 to 70% following the manufacturer’s protocol. For apoptosis dynamics, FCS, STED, and cPBSA measurements, the cells were transfected with 25 ng of target DNA and 975 ng of empty vector (pIRES-puro2 or pcDNA) for all used plasmids per two wells of an eight-well slide or one coverslip. For FRET measurements, the bicistronic plasmids were transfected using varying amounts of target DNA to cover a broad range of expression levels: For transfection in two wells, the combinations 25 ng of target DNA + 975 ng of empty vector, 100 ng of target DNA + 900 ng of empty vector, 250 ng of target DNA + 750 ng of empty vector, and 1000 ng of target DNA (no empty vector) were used. Donor-only (DO) controls (the monocistronic mEGFP fusion version of the proteins) were expressed at these varying concentrations as well. Live experiments or fixations were done 48 to 72 hours after transfection. For all live-cell experiments (time-lapse imaging, FCS, and FRET), the cells were incubated in Leibovitz’s L-15 medium (21083027, Gibco) without phenol red, supplemented with 10% fetal bovine serum (10500064, Gibco) and 1% penicillin/streptomycin (P0781, Sigma-Aldrich).

For all apoptosis experiments based on CD95L, the FasL, soluble (human) (recombinant) set (ALX-850-014-KI02, Enzo Life Sciences Inc., Loerrach, Germany) was used. The ligand was prepared according to the manufacturer’s protocol and further diluted in the respective cell culture or imaging medium. The provided enhancer was used for all experiments except FCS. For experiments using the enhancer, the enhancer concentration was always 100-fold higher than the ligand concentration. For all apoptosis experiments, the ligand concentration was 200 ng/ml, unless stated otherwise.

#### CD95 quantification by flow cytometry

The quantitative CD95 expression level of HeLa WT, HeLa CD95^KO^, and HeLa WT stable CD95-mEGFP was assessed using the QIFIKIT for quantification of cell surface antigens by flow cytometry (K007811-8, Agilent Technologies Inc., Santa Clara, CA, USA) on a MACSQuant Analyzer 10 (Miltenyi Biotec Miltenyi Biotec B.V. & Co. KG, Bergisch Gladbach, Germany) following the manufacturer’s protocol accurately. For CD95 detection, a monoclonal CD95 antibody (130-108-066, Miltenyi Biotec) was used. As negative control, an antibody against CD28 was used (70-0281, Tonbo A Cytek Brand, San Diego, CA, USA). As the secondary fluorescein isothiocyanate antibody provided with the QIFIKIT interfered with the mEGFP of the stably expressing CD95-mEGFP HeLa cell line, a secondary anti-mouse antibody conjugated to antigen-presenting cell (#17-4010-82, eBioscience, Thermo Fisher Scientific) was used for all samples. The measurement was repeated two times independently with *N* > 50,000 cells per measurement and sample. For HeLa CD95^KO^ with transient CD95-mEGFP, the number was not obtained from flow cytometry but from STED imaging spot density.

#### Cell fixation and immunostaining

For cPBSA and STED immunostaining, cells were fixed after transfection within the respective seeding vessel (see the “Cell culture, transfections, and ligand incubation” section). For experiments including the CD95L (Enzo Life Sciences Inc.), the ligand was incubated for 2 hours at 37°C before fixation.

Before fixation, cells were washed three times with cold washing buffer [Hanks’ balanced salt solution (14025050, Gibco) containing 0.1 M sucrose (57-50-1, Carl Roth GmbH + Co. KG, Karlsruhe, Germany) and 1% bovine serum albumin (A1391, ITW Reagents, AppliChem GmbH, Darmstadt, Germany)]. The fixation was performed using 4% methanol-free formaldehyde (28906, Thermo Fisher Scientific Inc.) in washing buffer for 10 min, shaking at room temperature. For STED, the fixation buffer additionally contained 0.1% glutaraldehyde (25% in H_2_O, G5882, Sigma-Aldrich), which was not used for cPBSA to reduce the fixation related green autofluorescence (AF) of the sample. Afterward, cells were washed three times again.

For cPBSA, as a last step, the cells were incubated with 750 mM tris [tris(hydroxymethyl)aminomethane, 103156X, VWR Chemicals, VWR International GmbH, Darmstadt, Germany] in Dulbecco’s phosphate-buffered saline (14190144, Gibco) to quench the AF of the formaldehyde. Afterward, cells were washed and immersed in Dulbecco’s phosphate-buffered saline.

For STED immunostaining, the next step was permeabilization with the washing buffer including 0.2% saponin (47036, Sigma-Aldrich) as permeabilizing reagent for 10 min. After 2× washing, the sample was blocked using a blocking buffer (Hanks’ balanced salt solution with 0.1 M sucrose and 4% bovine serum albumin) for 1 hour. For the staining step, the GFP-Booster Atto647N (gba647n-100, ChromoTek GmbH, Planegg-Martinsried, Germany) was diluted 1:200 in the blocking buffer and again incubated for 1 hour. Next, extensive washing was done using the washing buffer at least three times. As a last step, the coverslips were mounted upside down on a microscope slide using ProLong Diamond Antifade Mountant (P36965, Invitrogen, Life Technologies Inc., Carlsbad, CA, USA) and stored overnight before imaging.

### Microscope setups

#### Olympus IX83 wide-field system

The IX83 P2ZF inverted epifluorescence microscope system (Olympus Europa SE & Co. KG, Hamburg, Germany) was used for all wide-field and time-lapse measurements. The microscope is equipped with the motorized TANGO Desktop stage (Märzhäuser Wetzlar GmbH & Co. KG, Wetzlar, Germany) and the Photometrics Prime BSI camera (Teledyne Photometrics, Tucson, AZ, USA). An internal halogen lamp and the SOLA light engine (Lumencor Inc., Beaverton, OR, USA) served as light source for transmitted (bright-field, phase-contrast) and reflected (fluorescence) illumination, respectively.

#### Abberior expert line microscope

STED, cPBSA, and CELFIS measurements were performed on an Abberior expert line system as described previously (*64*) (Abberior Instruments GmbH, Göttingen, Germany). In addition, polarization control for cPBSA measurements was achieved using λ/2 and λ/4 wave plates (Abberior Instruments) and a SK010PA-vis 450- to 800-nm polarization analyzer (Schäfter Kirchhoff GmbH, Hamburg, Germany). Cells were kept at 37°C using a heating insert HP-LabTek (PeCon GmbH, Erbach, Germany). The instrument is operated using the customized Abberior microscope software Imspector (version 14.0.3060, Abberior Instruments GmbH).

#### Confocal microscope for multiparameter image spectroscopy (MFIS)

FCS data were recorded using a confocal microscope for MFIS (*15*) equipped with pulsed excitation and polarization-sensitized time-correlated single-photon counting (TCSPC) readout. Excitation light was created using a Sepia II (PicoQuant GmbH, Berlin, Germany) driving an LDH-D-C-485 laser head (PicoQuant) and coupled to a FluoView1000 IX81 inverted microscope (Olympus, Shinjuku, Japan). Light was focused to a diffraction-limited spot using a 60× water immersion UPLSAPO 1.2 numerical aperture (NA) objective (Olympus), and emitted light was separated using a DM405/488/559/635 quadband mirror (Olympus). Emitted fluorescence was split into perpendicular and parallel components using a polarizing beam splitter and measured using a BrightLine Fluorescence Filter 520/35 (Semrock Inc., Rochester, NY, USA) and PDM series avalanche photo diodes (Micro Photon Devices, Bolzano, Italy) for each channel. Electronic pulses were converted to photon events using a HydraHarp (PicoQuant). Cells were kept at 37°C using a heating insert HP-LabTek (PeCon GmbH, Erbach, Germany).

### Analysis of spectroscopy and images

#### Time-lapse imaging for apoptosis dynamics

The time-lapse measurements were performed on an IX83 inverted epifluorescence microscope system (Olympus Europa SE & Co. KG, Hamburg, Germany) (details in the “Microscope setups” section) using either a 20× oil objective (NA, 0.85; UPLSAPO20xO) or a 60× oil-objective (NA, 0.65 to 1.25; UPLFLN60XOIPH) on a temperature-controlled on-stage heating system (PeCon GmbH, Ulm, Germany) at 37°C. The CD95L (Enzo Life Sciences Inc.) (see the “Cell culture, transfections, and ligand incubation” section) was added to the cells to the desired final concentration on the microscope. Time-lapse videos were acquired with the cellSens Dimensions software (Olympus) by sequential imaging of the phase-contrast channel and, if available, the mEGFP channel (excitation, 470/40 nm; emission, 525/50 nm) at multiple positions every 5 to 15 min over 10 hours. Image analysis was performed with Fiji (*65*), using an intensity-based threshold to the fluorescence channel (via the tree command image > adjust > threshold) to detect successfully transfected cells. The death time of each single cell undergoing apoptosis, which shows membrane blebbing and cell fragmentation, was identified manually from time-lapse video in Fiji. The frame number of each apoptosis event was marked individually, and all frame numbers were exported to a table. This table was loaded into MATLAB and fitted with the Hill equation.

For a mathematical description of the sigmoidal apoptosis dynamics curves *P*(*t*), they were fitted (MATLAB R2019a, The MathWorks Inc.) using the Hill equation to characterize the dynamics and efficiency of the cell response

Pt=Pmax−Pmax−Pmin1+tthalfn (1)

where *P*_min_ and *P*_max_ are the minimal and maximal fractions of apoptotic cells and *t*_half_ is the characteristic time after which half of all apoptotic cells died. The Hill coefficient (also cooperativity coefficient) *n* indicates how fast the signal is transduced.

#### FCS measurements

For sample preparation, see the “Cell culture, transfections, and ligand incubation” section.

##### Calibration

Calibration of the MFIS setup was performed according to established procedures in our research group (*66*). Briefly, the optimal correction collar setting was found by minimizing the number of Rhodamine 110 (#83695, Sigma-Aldrich) molecules in the focus. For all our experiments, the correction collar matched our coverslip thickness (170 μm). The instrument response function (IRF) was measured using a mirror to enable TCSPC analyses. Next, a Rhodamine 110 solution with one to five molecules in the focus was measured to obtain (i) a calibration for the confocal volume shape factor, *z*_0_/ω_0_ or κ; (ii) the ratio of the parallel and perpendicular detection efficiency, γ; (iii) the number and brightness of Rhodamine 110 molecules in the focus; and (iv) the confocal detection volume by assuming a Rhodamine 110 diffusion constant of *D* = 430 μm2/s for recordings at room temperature (22.5°C) (*67*) or 600 μm^2^/s when it was recorded at 37°C.

The laser power was measured at the sample using an immersion S170C power meter head (Thorlabs GmbH, Lübeck, Germany) attached to a PM400 power meter body (Thorlabs GmbH, Lübeck, Germany). As the power varied by ~10% when translating in *x*, *y*, and *z*, we avoided a systematic error by varying the position until the maximum power was reached.

##### Recording procedure

A confocal microscope was used to bring the bottom membrane in focus. The diffraction-limited focus was placed in a stationary position away from the edge of the cell and away from the endoplasmic reticulum and Golgi apparatus. FCS curves were recorded during 5 min using a 5-μW 488-nm pulsed excitation beam, a 200-μm or 2.1–arbitrary unit pinhole, a 60× water objective and polarization-sensitized readout [see the “Confocal microscope for multiparameter image spectroscopy (MFIS)” section]. Solution measurements were performed using identical settings except for placing the focus 50 μm above the glass surface and recording Rhodamine 110 and mEGFP for 1 and 5 min, respectively.

##### FCS curve fitting

All cell measurements were fitted with two diffusion terms, corresponding to a cytoplasmic (cp) and a membrane (mem) component

$$\begin{array}{c} G\left(t_{c}\right)=1+\frac{\rho_{\text{cp}}}{\left(1+\frac{t_{c}}{t_{\text{diff},\text{cp}}}\right){\left(1+\frac{t_{c}}{\kappa^{2}\;t_{\text{diff},\text{cp}}}\right)}^{0.5}}\\ +\frac{\rho_{\text{mem}}}{\left(1+\frac{t_{c}}{t_{\text{diff},\text{mem}}}\right){\left(1+\frac{t_{c}}{\kappa^{2}\;t_{\text{diff},\text{mem}}}\right)}^{0.5}}+G\left(\infty \right) \end{array}$$ (2)

where ρ denotes the species correlation amplitude, *t*_diff_ is the species diffusion time, *G*(∞) is the residual correlation at infinity, κ^2^ is the aspect ratio of the focus, and *t* is the correlation time. As the signal-to-noise ratio was limited, the stability of the fit was improved by not fitting an additional bunching term to account for triplet as it did not affect the values of the diffusion times. To improve the stability of the fit further, a covariance between *t*_diff, cp_ and *t*_diff, mem_ was fitted globally over a set of 11 points from seven CD95-transfected cells, yielding a diffusion time of 0.60 ms to be kept fixed for all subsequent analyses. For more information about obtaining robust results from noisy live-cell FCS data, see notes S1 and S2.

Curve weighting according to σ_AV_ (*68*) was preferred because of its ability to provide accurate weights at long correlation times. Our measurements fulfilled the requirement for that the recording can be divided in >10 chunks of 20 s each. FCS curves were created and fitted using the SymPhoTime software (PicoQuant GmbH, Berlin, Germany).

#### Cell lifetime FRET image spectroscopy

##### TCSPC fitting

The following four models were used to fit the fluorescence TCSPC decays. Which model was used for which condition and the subsequent steps undertaken are described in the “Strategy of fitting” section. The theoretical models are indicated by an index “mod” in the *f*(*t*) expression, whereas the experimental data have an index “exp” in the *f*(*t*) expression. All applied models use a homogeneous approximation, i.e., for each FRET species, the set of DO and depolarization rate constants are the same. In our microscope for multiparameter image spectroscopy (*15*), we have polarization-resolved detection. In our experiments, the polarization of the linear-polarized laser excitation was set to the *x* coordinate of the microscope body, and polarization-resolved detection is oriented relative to this. For convenience when describing the spectroscopy effects with respect to polarization, we refer to the excitation polarizer (*p*) and respective analyzer (*a*). The polarization can have a vertical (V) or a horizontal (H) orientation with respect to the laboratory frame. In our case, the combined polarizer-analyzer positions (pa) can be VV or VH. In the following, this is written as pa ∈ {VV,VH}.

Model 1. DO-VM: DO total fluorescence intensity decay without polarization effects (equivalent to magic angle)*.* This model is used to fit the measured DO fluorescence decay after donor excitation, when (i) the signal is recorded at the so-called “magic angle” setting of an emission polarizer or when (ii) a composite signal (VM) is recorded, consisting of two signals measured at parallel (VV) and perpendicular (VH) setting of the emission polarizers. The fluorescence signal, $f_{\text{VM}}^{\left(\text{DO}\right)\text{mod}}$ , reads

$$\begin{array}{c} f_{\text{VM}}^{\left(\text{DO}\right)\text{mod}}\left(t\right)=a_{0}\\ \left(\left[f^{\left(\text{DO}\right)}\left(t\right)+a_{\text{AF}}\;{\text{AF}}_{\text{VM}}\left(t\right)\right]\overset{T}{⊛}{\hat{\text{IRF}}}_{\text{VM}}\left(t;t_{\text{sh}}\right)+a_{\text{sc},\text{VM}}\;{\hat{\text{IRF}}}_{\text{VM}}\left(t;t_{\text{sh}}\right)\right)\\ +{\text{BG}}_{\text{VM}}^{\left(\text{DO}\right)};f^{\left(\text{DO}\right)}\left(t\right)=\sum_{i=1}^{n_{d}}p_{i}^{\left(\text{DO}\right)}e^{-k_{i}^{\left(\text{DO}\right)}t};\\ \sum_{i=1}^{n_{d}}p_{i}^{\left(\text{DO}\right)}=1;k_{i}^{\left(\text{DO}\right)}=\frac{1}{\tau_{i}^{\left(\text{DO}\right)}} \end{array}$$ (3)

Here, *a*_0_ is the DO decay amplitude prefactor for the photon counts; $p_{i}^{\left(\text{DO}\right)}$ and $k_{i}^{\left(\text{DO}\right)}$ are the fraction and rate constant of the S1 singlet excitation state depopulation [inverse of the fluorescence lifetime $\tau_{i}^{\left(\text{DO}\right)}$ (*69*)] for the DO components (DO decay parameters), respectively; *n*_d_ is the number of DO components; AF_VM_(*t*) and *a*_AF_ are the independently measured AF profile and its amplitude; ${\hat{\text{IRF}}}_{\text{VM}}\left(t;t_{\mathrm{sh}}\right)$ is the independently measured IRF normalized to the unit integral; *t*_sh_ is the time shift relative to the DO decay; *a*_sc, VM_ is the amplitude of the scattered light; and ${\text{BG}}_{\text{VM}}^{\left(\text{DO}\right)}$ is the absolute background (BG) value. The operator “$\overset{T}{⊛}$” designates the circular convolution with the repetition period *T* accounting for the overlap of the exponential decays. In practice, the convolution of IRF with time shifts of IRF is implemented using the Fourier transform and multiplication in frequency space. *f*^(DO)^(*t*) indicates the ideal signal without any modification due to AF, IRF, scattered light, or BG signal.

Model 2. DA-VM: total fluorescence intensity decay in presence of FRET without polarization effects (equivalent to magic angle)*.* The expression of the VM signal in the presence of FRET is almost the same as for the DO case (Eq. 3), except for the multiexponential decay *f*^(DO)^(*t*) being replaced by the *f*^(DA)^(*t*) decay. In most cases, several species contribute to the fluorescence decay of the FRET sample *f*^(DA)^(*t*): DO species (*j* = 0), DA no-FRET species (*j* = 0) (see cloud correction in Fig. 3C), and FRET-active DA species (*j* > 0). Thus, Eq. 4 describes a mixture of donors where only a fraction is quenched by FRET. This feature is exploited in this work to monitor CD95 oligomerization by FRET

$$\begin{array}{c} f^{\left(\text{DA}\right)}\left(t\right)=\sum_{i=1}^{n_{d}}\sum_{j=0}^{n_{f}}p_{i,j}^{\left(\text{DA}\right)}e^{-k_{i,j}^{\left(\text{DA}\right)}t};\\ p_{\mathrm{ij}}^{\left(\text{DA}\right)}=p_{i}^{\left(\text{DO}\right)}p_{j}^{\left(\text{FRET}\right)};\sum_{j=0}^{n_{f}}p_{j}^{\left(\text{FRET}\right)}=1;k_{i,j}^{\left(\text{DA}\right)}=\left\{\begin{array}{cc} k_{i}^{\left(\text{DO}\right)}+k_{j}^{\left(\text{FRET}\right)}, & j>0\\ k_{i}^{\left(\text{DO}\right)}, & j=0 \end{array}\right. \end{array}$$ (4)

Here, in addition to the DO model above (Eq. 3), the following parameters are added: $p_{j}^{\left(\text{FRET}\right)}$ and $k_{j}^{\left(\text{FRET}\right)}$ are the FRET fractions and rate constants of the individual FRET states (DA decay parameters), and *n*_f_ is their number. The fraction of donor fluorophores *x*_FRET_, which is quenched by FRET, is determined from the sum of fitted FRET rate fractions with *j* > 0

$$x_{\text{FRET}}=\sum_{j=1}^{n_{f}}p_{j}^{\left(\text{FRET}\right)}$$ (5a)

With this, it is convenient to define another set of FRET rate amplitudes $a_{j}^{\left(\text{FRET}\right)}$ (normalized to unity) as follows

$$\begin{array}{c} a_{j}^{\left(\text{FRET}\right)}=\frac{p_{j}^{\left(\text{FRET}\right)}}{\sum_{j=1}^{n_{f}}p_{j}^{\left(\text{FRET}\right)}};j\in \left\{1\ldots n_{f}\right\}\\ x_{\text{FRET}}=1-p_{0}^{\left(\text{FRET}\right)} \end{array}$$ (5b)

From fitting the fluorescence lifetime decay of DA samples and theoretical accessible volume simulations of the DA pairs (see step 7 from the “Strategy of fitting” section), we can determine the rate constants $k_{j}^{\left(\text{FRET}\right)}$ and corresponding FRET fractions $p_{j}^{\left(\text{FRET}\right)}$ for *j* > 0 and calculate $a_{j}^{\left(\text{FRET}\right)}$ . This prior knowledge is used in the final fits of noisy single-cell data by fixing $a_{j}^{\left(\text{FRET}\right)}$ for *j* > 0 and $k_{j}^{\left(\text{FRET}\right)}$.

Model 3. {DO-VV, DO-VH}: DO fluorescence decay with polarization-resolved detection. For this work, the polarization-resolved detection has the advantage that joint fitting of the VV and VH fluorescence decays allows for a better accounting for the individual shapes of the IRFs in the VV and VH channels that are a priori different. Moreover, accounting for the variance of the VV and VH signals is simpler and more accurate than using the composite VM signal. As a result, the accuracy of the global fit is usually better than the accuracy of the composite VM signal fit

$$\begin{array}{c} \left\{\begin{array}{c} f_{\text{VV}}^{\left(\text{DO}\right)\text{mod}}\left(t\right)=a_{0}\left(\left[f_{V}\left(t;l_{1},l_{2}\right)+a_{\text{AF}}\;{\text{AF}}_{\text{VV}}\left(t\right)\right]\overset{T}{⊛}{\hat{\text{IRF}}}_{\text{VV}}\left(t;t_{\text{sh},\text{VV}}\right)+a_{\text{sc},\text{VV}}\;{\hat{\text{IRF}}}_{\text{VV}}\left(t;t_{\text{sh},\text{VV}}\right)\right)+{\text{BG}}_{\text{VV}};\\ f_{\text{VH}}^{\left(\text{DO}\right)\text{mod}}\left(t\right)=a_{0}\left(\left[f_{H}\left(t;G,l_{1},l_{2}\right)+a_{\text{AF}}\;{\text{AF}}_{\text{VH}}\left(t\right)\right]\overset{T}{⊛}{\hat{\text{IRF}}}_{\text{VH}}\left(t;t_{\text{sh},\text{VH}}\right)\right)+{\text{BG}}_{\text{VH}}; \end{array}\right.\\ \left\{\begin{array}{c} f_{V}\left(t;l_{1},l_{2}\right)=\left(f_{\text{VH}}^{\left(\text{DO}\right)}\left(t\right)-f_{\text{VV}}^{\left(\text{DO}\right)}\left(t\right)\right)+l_{1}\;f_{\text{VV}}^{\left(\text{DO}\right)}\left(t\right)\\ f_{H}\left(t;G,l_{1},l_{2}\right)=G\;\left[\left(f_{\text{VH}}^{\left(\text{DO}\right)}\left(t\right)-f_{\text{VV}}^{\left(\text{DO}\right)}\left(t\right)\right)-l_{2}\;f_{\text{VH}}^{\left(\text{DO}\right)}\left(t\right)\right] \end{array}\right.\\ f_{\text{pa}}^{\left(\text{DO}\right)}\left(t\right)=f^{\left(\text{DO}\right)}\left(t\right)+P_{\text{pa}}\;r_{0}\sum_{i=1}^{n_{d}}\sum_{l=1}^{n_{a}}p_{i}^{\left(\text{DO}\right)}p_{l}^{\text{AN}}e^{-\left(k_{i}^{\left(\text{DO}\right)}+k_{l}^{\text{AN}}\right)t};\text{pa}\in \left\{\text{VV},\text{VH}\right\};\\ P_{\text{VV}}=2;P_{\text{VH}}=-1;\\ \sum_{l=1}^{n_{a}}p_{l}^{\text{AN}}=1;k_{l}^{\text{AN}}=\frac{1}{\rho_{l}} \end{array}$$ (6)

Here, in addition to the DO-VM model above (Eq. 3), the following parameters are added: $p_{l}^{\text{AN}}$ and $k_{l}^{\text{AN}}$ are the fractions and depolarization rate constants of the individual anisotropy states (anisotropy decay parameters); *n*_a_ is the number of anisotropy states; *r*_0_ is the initial (fundamental) anisotropy; *G* is the factor accounting for the different polarization-dependent detection efficiencies *g*_VV_ and *g*_VH_ of the V and H channels, respectively (i.e., *G* = *g*_VH_/*g*_VV_ = 1.087); and *l*_1_ and *l*_2_ are factors accounting for the imperfection of the detection lens (*l*_1_ and *l*_2_ were small and set to zero in this work for convenience). The VV and VH components of AF, IRF, time shifts, and BG are accounted for in the components of the corresponding decay only. The scattered light is accounted for in the VV signal only.

Model 4. {DA-VV, DA-VH}: total fluorescence intensity decay in presence of FRET with polarization-resolved detection. The expression for this model is mostly the same as in the case of the {DO-VV, DO-VH} model above (Eq. 6), except that the DO multiexponential decays $f_{\text{pa}}^{\left(\text{DO}\right)}\left(t\right)$ are replaced by DA decays of the form

$$\begin{array}{c} f_{\text{pa}}^{\left(\text{DA}\right)}\left(t\right)=f^{\left(\text{DA}\right)}\left(t\right)+P_{\text{pa}}\;r_{0}\sum_{i=1}^{n_{d}}\sum_{j=0}^{n_{f}}\sum_{l=1}^{n_{a}}p_{i,j}^{\left(\text{DA}\right)}p_{l}^{\text{AN}}e^{-\left(k_{i,j}^{\left(\text{DA}\right)}+k_{l}^{\text{AN}}\right)t};\\ \text{pa}\in \left\{\text{VV},\text{VH}\right\} \end{array}$$ (7)

The DO fluorescence decays and models DO-VM and {DO-VV, DO-VH} are recovered from the models DA-VM (Eq. 4) and {DA-VV, DA-VH} (Eq. 7), by reducing the DA decay parameters to the single component with $k_{0}^{\text{FRET}}$ =0, i.e., leaving only *j* = 0 in the expressions above. Hence, in practice, the models DA-VM and {DA-VV, DA-VH} with $k_{0}^{\text{FRET}}$ =0 were used for fitting the DO fluorescence decays as well.

##### Minimized quantities in the fit

Two quantities were tested as minimized goodness-of-fit functions: a reduced least squares, $\chi_{r,\text{LS}}^{2}$ , assuming the normal distribution of counts and maximum-likelihood estimate of χ^2^ assuming a Poisson distribution of counts (*70*)

$$\chi_{r,\text{LS}}^{2}=\frac{1}{n_{\text{exp}}-n_{\text{par}}}\;\sum_{i=1}^{n_{\text{exp}}}\frac{{\left(f^{\text{exp}}\left(t_{i}\right)-f^{\text{mod}}\left(t_{i}\right)\right)}^{2}}{w_{i}}$$ (8)

$$\chi_{\text{ML}}^{2}=2\left[N_{\text{mod}}-N_{\text{exp}}+\sum_{i=1}^{n_{\text{exp}}\;}f^{\text{exp}}\left(t_{i}\right)\text{ln}\left(\frac{f^{\text{exp}}\left(t_{i}\right)}{f^{\text{mod}}\left(t_{i}\right)}\right)\right]$$ (9)

where *f*^exp^(*t_i_*) is experimental counts uniformly time-binned with mean bin times *t_i_*; *f*^mod^(*t_i_*) is the model counts calculated at the same *t_i_*; *n*_exp_ is the number of fitted time bins; *n*_par_ is the number of fitted parameters; *w_i_* is the weights for each bin; *N*_exp_ is the total counts of the data; and *N*_mod_ is the total counts of the model. For the measured data (VV and VH), weights *w_i_* were chosen equal to the measured counts, which follow a Poisson distribution. For the composite VM signal, weights were calculated using the uncertainty propagation rule.

In agreement with Maus *et al.* (*71*), we have found that the maximum likelihood estimate of χ^2^ (Eq. 9) gives a more accurate fitting result for data with a low number of counts. This occurs especially at the tail of fluorescence decays. For this reason, $\chi_{\text{ML}}^{2}$ was used to get accurate values of *x*_FRET_. In case of data with a high number of counts, $\chi_{\text{ML}}^{2}$ and $\chi_{r,\text{LS}}^{2}$ performed similarly.

##### Strategy of fitting

To achieve the most robust fit results, we performed a so-called “pattern fit,” where a fixed pattern of parameters describing the donor quenching by FRET (e.g., $p_{i}^{\left(\text{DO}\right)}$ and $k_{i}^{\left(\text{DO}\right)}$ ) together with the independently measured IRF and AF is used. In the following, steps 1 to 6 describe how we determined the parameters of the pattern and which effects were taken into account. The final pattern fit is described in step 7.

1)For the parallel (VV) and the perpendicular (VH) channels, the IRF and the AF were measured. The IRF and AF were determined for the applied excitation/depletion lasers using the following conditions: (i) excitation at 561 nm with aqueous solution of erythrosine in 5 M potassium iodide as quencher and (ii) excitation at 640 nm with aqueous solution of malachite green. The measured AF signal was fitted with the third, {DO-VV, DO-VH} model (Eq. 6) to obtain the AF profile. Here, *f*_pa_(*t*) is the AF or IRF. From this, the total intensity (VM) data for IRF and AF was calculated according to

$$f_{\text{VM}}\left(t\right)=f_{\text{VV}}\left(t\right)+G\;2\;f_{\text{VH}}\left(t\right)$$ (10)

2)About 100 to 200 DO fluorescence decays with VV and VH signals were measured for each sample from different regions of interest (ROIs). Similarly, about 100 to 200 DA fluorescence decays with parallel (VV) and perpendicular (VH) fluorescence decays were measured for each sample from different ROIs.

3)The DO-VM and DA-VM signals were calculated by Eq. 10. For subsequent fits, the weights of bins for VM decays were calculated according to the uncertainty propagation rule

$$w_{i}=f_{\text{VV}}\left(t_{i}\right)+G^{2}\;4\;f_{\text{VH}}\left(t_{i}\right)$$ (11)

4)The whole set of *f*_VM_(*t*) decays for the given sample was first fitted jointly by DO-VM model (Eq. 3) using $\chi_{r,\text{LS}}^{2}$ as goodness-of-fit functions (Eq. 8) to get the common DO decay parameters [fluorescence rate constants and their fractions, $p_{i}^{\left(\text{DO}\right)}$ and $k_{i}^{\left(\text{DO}\right)}$ ]. It was found that two components of DO (*n*_d_ = 2) were sufficient to accurately fit the DO data. Adding an additional component to the fit did not decrease $\chi_{r,\text{LS}}^{2}$ considerably.

5)The DO decay parameters obtained at step 4 were used as fixed parameter for the subsequent fit of the FRET-VM data (Eqs. 4, 5a, and 5b). As result of the joint fitting of the FRET data, the DA decay parameters [ $p_{\mathrm{ij}}^{\left(\text{DA}\right)}$ and $k_{i,j}^{\left(\text{DA}\right)}$ ] and a preliminary estimate of the *x*_FRET_ fraction were obtained. We found that two components of the DA decay parameters were sufficient for accurately fit the DA data. Adding an additional component to the fit did not decrease $\chi_{r,\text{LS}}^{2}$ significantly.

6)The final DO decay parameters [ $p_{i}^{\left(\text{DO}\right)}$ and $k_{i}^{\left(\text{DO}\right)}$ ], depolarization decay parameters ( $p_{l}^{\text{AN}}$ and $k_{l}^{\text{AN}}$ ), and values of *r*_0_ were determined by a fit to the {DO-VV, DO-VH} model (Eq. 6) with $\chi_{\text{ML}}^{2}$ (Eq. 9) as goodness-of-fit function. We found that a single depolarization time ρ was sufficient to accurately fit the data. To compare the accuracy of the joint {DO-VV, DO-VH} fit with the accuracy of the DO-VM fit, the $\chi_{r,\text{LS}}^{2}$ (Eq. 8) was also calculated. The global {DO-VV, DO-VH} fit with $\chi_{\text{ML}}^{2}$ increased the accuracy.

7)Description of pattern fit. To describe DA data of single cells by the {DA-VV, DA-VH} model (Eq. 7), we fitted the key parameter of interest, *x*_FRET_, and a single depolarization time using the fundamental anisotropy *r*_0_ as start value. A typical fit value for the depolarization time ρ was ~45 ns with *r*_0_ = 0.37. This depolarization time is expected for a flexibly tethered fluorescent protein. Here, the following parameters were fixed to values predetermined at previous steps: (i) DO decay parameters with two lifetimes and species fractions { $\tau_{i}^{\left(\text{DO}\right)}$;[$p_{i}^{\left(\text{DO}\right)}$]:1.68 ns; (0.5) and 2.75 ns; (0.5)}, derived in step 6; and (ii) DA decay parameters with two FRET rate constants and corresponding amplitudes { $k_{j}^{\left(\text{FRET}\right)}$ ; [ $a_{j}^{\left(\text{FRET}\right)}$ ]: 0.154 ns^−1^; (0.75) and 1.346 ns^−1^; (0.25)} obtained in step 5. The found values very well cover the typical range (and according donor-acceptor distances) that can be resolved by time-resolved FRET measurements (*34*). The lower limit for the FRET rate constants is defined by the time resolution of the setup and the upper limit by the Förster radius of the FRET pair, so that the DA decay sufficiently differs from a DO decay. In this way, the pattern is most stable, and *x*_FRET_ has the least possible cross-talk to other parameters chosen for the pattern fit.

Note that while noise effects are minimized by a pattern fit, the data exhibit a *x*_FRET_ scatter of ~10%. The scatter arises from three contributions: (i) the concentration dependence of CD95 given by slightly different expression levels (i.e., population heterogeneity); (ii) changes in the dimerization/trimerization fraction depending on the overall protein concentration or due to structural changes (e.g., removal of the DD can alter the intermolecular interaction); and (iii) the shot noise [see Maus *et al.* (*71*)].

##### Determination of oligomer fraction

The oligomer fraction was obtained from *x*_FRET_ by calculating the FRET signal corresponding to a pure dimer, *x*_FRET, max_ (see note S3). To calculate the latter, the following steps were taken: (i) Accessible volume simulations were performed in the program Olga (*41*), assuming a 51–amino acid linker and effective FRET range up to 82 Å using a solution nuclear magnetic resonance model of trimeric CD95 TM domains [Protein Data Bank id: 2NA7 (*45*)] to set the anchor points for all structures. From this, the cloud correction factor ξ = 0.465 was determined. (ii) From the abundance of mEGFP and mCherry fluorophores in the sample, a *p*_AD_ = 71% abundance of heterodimers compared to 23% active/active and 6% active/inactive homodimers for CD95 variants and CD86 was calculated, and for CTLA4, a *p*_AD_ = 78% abundance of heterodimers compared to 18% active/active and 4% active/inactive homodimers was derived. (iii) A 80% (*43*, *44*) maturation factor was used for mEGFP. Since receptors with the acceptor were expressed in large excess compared to the donor, the maturation correction for mCherry was negligible. In addition, the mEGFP- and mCherry-coupled receptor concentrations were determined using a molecular brightness of 814 and 264 Hz per molecule per microwatt, respectively, also accounting for the 80% mEGFP maturation and molecular fractions localizing to the membrane (~60%) and the cytoplasmn (~40%) as determined with FCS (see table S4).

#### STED imaging and analysis

STED images were recorded on the Abberior expert line setup (Abberior Instruments GmbH; details in the “Microscope setups” section). All immunostained samples (see the “Cell fixation and immunostaining” section) were imaged with a 640-nm excitation laser (5.3 μW) and a 775-nm STED depletion laser (41 mW) using an oil-immersion objective (NA, 1.4; UPLSAPO 100XO, Olympus Europa SE & Co. KG). Before the measurements, channel alignment was performed manually using TetraSpeck microspheres (T7279, Invitrogen). 20 ROIs of 5 μm by 5 μm (10-nm pixel size, 4.00-μs dwell time, and 5 frames) of the bottom membrane of 10 cells were recorded.

##### Deconvolution and spot center detection on STED data

As a first step of data processing, the first 2.2 ns of the recording was time-gated to increase the achievable resolution using the home-built programm AnI. The sum of the parallel and perpendicular polarized images was used for further analysis. For deconvolution and image data analysis, Huygens Professional software (HuPro Version 21.10.1p2 64b, Scientific Volume Imaging B.V., Hilversum, the Netherlands) was used. The deconvolution was performed using the CMLE (classic maximum likelihood estimation) algorithm with a signal-to noise ratio of 3. The convergence stop criterium was set to 0.01 or a maximum of 40 iterations. The applied BG mode was manual, with a fixed of 0.7. After deconvolution, the Object Analyzer of Huygens was used to identify the objects and their locations on the membrane. The global object threshold was 1.2 with a seeding level of 1.3, and the garbage volume was two voxels. Objects touching the image border were excluded from the analysis. Detected spot centers were used to fit a 2D Gaussian distribution with a size of 11 pixels by 11 pixels to the respective spot in the raw STED data.

##### Simulation of STED images

To model the mean object surface density observed in STED images, we have simulated composite large images of 2500 pixels by 2500 pixels with a total of 10,000 objects (monomer, dimer, trimer, and dimer of trimers, i.e., containing *n* = 1, 2, 3, or 6 fluorophores at defined positions within a single point spread function (see fig. S10). Objects were randomly distributed over the image surface to mimic crowding effects in experimental images. Each fluorophore in the object is considered to behave as a dipole for which the following applies: Since circularly polarized excitation was used in the experiment, all objects with fluorophore dipole orientation in the *XY* plane would exhibit very similar brightness, while any angle between the fluorophore dipole and *Z* axis different from 90° would give rise to a distribution of brightness. Overall, three noise sources considered in the following: (i) normally distributed noise arising from the variation of spot brightness, (ii) the Poisson noise arising from the photons emitted by the fluorophore, and (iii) a Poisson noise arising from the photons in the BG of the image.

In our experiment, the spot brightness varies since circularly polarized excitation is used. Here, all fluorophore dipoles in the *XY* plane exhibit similar brightness, whereas dipoles nonperpendicular to the *Z* axis give rise to a distribution of brightness. In our case, the fluorophore dipoles were randomly distributed (see fig. S12), giving rise to brightness changes, which, in the monomer control, resulted in a brightness distribution around the average amplitude 〈*Q*〉 by ±30%. Hence, we modeled the emitted photon distribution by the fluorophore as 2D Gaussian based on the experimental data of the monomer control, with a diffraction-limited width of σ*_X_* = σ*_Y_*, average brightness 〈*Q*〉, and normally distributed noise of 〈*Q*〉 (i.e., brightness variation) with σ_〈*Q*〉_ = 0.3 x 〈*Q*〉 (see table S3).

The simulation of composite images was done as follows:

1)First 10,000 objects were generated placing 2D Gaussians with *Q_i_* (*i* = 1.0.6) amplitudes normally distributed around their mean value 〈*Q*〉 according to σ_〈*Q*〉_ and fixed SD σ*_X_* on a 25-pixel by 25-pixel region.

2)From the photon counts in each pixel of the 25 by 25 image, the Poisson noise was calculated, and a value was added to each pixel without BG.

3)A large image of 2500 pixels by 2500 pixels with 0 photon counts in each pixel was generated.

4)A total of 10,000 random (*X*, *Y*) coordinates in the range (0.2475, 0.2475) were generated as reference coordinate for the (0, 0) pixel location of the 25-pixel by 25-pixel images, which were added to the large image.

5)Last, as noise BG, a Poisson-distributed value with mean 〈BG〉 was added to each pixel of the composite image.

6)The composite image was saved as U16 TIF image and analyzed by the home-built written LabView2020-based AnI-3SF software.

##### Analysis of simulated STED images

Simulated images were analyzed in a similar way as experimental STED images with the only difference that the algorithm of the AnI-3SF software for confocal multiparameter fluorescence imaging and spectroscopy (www.mpc.hhu.de/software/mfis-2021) was used for the spot search (a threshold of one photon and a minimum object size of 9 pixel). A 2D Gaussian distribution of size 11 pixels by 11 pixels was fitted to each detected spot center.

##### Spot anisotropy analysis

The spot intensities of the parallel (P) and perpendicular (S) channel, *I*_P_ and *I*_S_, were determined with an individual object analysis of both images as described in the section Simulation of STED images. The steady-state anisotropy *r* was calculated by

$$r=\frac{I_{P}-{\mathrm{GI}}_{S}}{I_{P}+2{\mathrm{GI}}_{S}}$$ (12)

where the polarization correction factor *G* = η_S_/η_P_ corrects for the instrument’s polarization–dependent transmission. η_P_ and η_S_ are the detection efficiencies of the parallel and perpendicular detection channels. The polarization correction factor *G* was determined to be 1.087.

##### Pair correlation

The distribution of object points (spot centers) was analyzed using the pair correlation function *g*(*r*) (*72*)

$$g\left(r\right)=\frac{1}{\pi \rho^{2}r\gamma \left(r\right)}\sum_{i=1}^{N}\sum_{j=i+1}^{N}k\left(r-\left|p_{i}-p_{j}\right|\right)$$ (13)

where ρ is the object density in the image and |*p_i_* − *p_j_*| is the distance between two object points *p* with 2D position (*x*, *y*). The object positions were assumed to be planar. The covariance function γ and kernel *k* are defined in (*73*).

The pair correlation of the objects found by the Huygens Object Analyzer was calculated using a self-written MATLAB script (R2019a, The MathWorks Inc.) following the example of (*73*). The correlation histogram *g*(*r*) was calculated for binned distances with a bin width of 10 nm and a bandwidth of 5 nm. The data of all STED images per sample were averaged.

To compare the pair correlation of real STED images with a simulation of randomly distributed objects, we simulated images comparable to the real data. Using MATLAB (R2019a), 500-pixel by 500-pixel images with randomly distributed object centers were created. The number of object points per image was selected randomly between 300 and 600 per image, and the pixel value was adjusted to 4 (photons per pixel) to match the real data average. Next, the spots were filtered using a 2D Gaussian smoothing kernel with SD of σ = 2.5 pixels. Subsequently, 20 simulated images were analyzed using the Huygens Object Analyzer similar to the real data as described in Deconvolution and spot center detection on STED data, and, last, the pair correlation *g*(*r*) of simulated data was calculated.

#### Confocal photobleaching step analysis

cPBSA measurements were performed on the Abberior setup (compare the “Microscope setups” section) using circularly polarized light and a 100XO objective (NA, 1.4; UPLSAPO, Olympus). We ensured that a single membrane layer was in focus by measuring the area underneath the nucleus and record bleaching traces away from the cell edge (see fig. S13).

##### Automated data acquisition script

Data acquisition using a confocal microscope is generally slower than total internal reflection fluorescence–based PBSA because only one molecular assembly can be measured simultaneously. To gather sufficient statistics, a data acquisition script was written that automates data acquisition after a manual membrane area selection. The program uses the Python application programming interface from the Imspector acquisition software and contains a graphical user interface. The data acquisition works as follows:

1)A suitable area (20 μm by 20 μm) is selected on the lower membrane by the user.

2)An overview image is recorded using 50-nm pixel size, 10-μs dwell time, and 5% 488-nm excitation and summed over three frames. The output corresponding to 5% laser power fluctuated around 1.3 μW (see table S2).

3)The overview image is smoothened using a Gaussian filter with a SD (σ) of 1 pixel.

4)Molecular assemblies are identified from local maxima that exceed three to five counts on the smoothed image. The threshold level was adjusted per area as needed to select all spots while avoiding crowding by visual inspection.

5)Local maxima that are closer than 450 nm to any other local maxima are not considered for further analysis.

6)A photon trace is recorded for each remaining local maximum by placing the confocal beam there for a duration of 3 s.

7)A quick display is rendered for user feedback.

##### Data quality optimization

We established an experimental procedure to optimize the quality of our data. First, our sample fixation procedure minimizes AF. Second, only molecular assemblies that are below the nucleus were recorded to ensure that the lower membrane was not in close proximity to the top membrane, as cells partly deflate upon fixation (see fig. S13). To avoid this effect, we forgo upside-down mounting on a cover slip and image cells in well slides instead. Third, low excitation power and integration time were used for creating an overview image to avoid premature bleaching.

##### Data analysis

Data analysis was done using the KV algorithm (*48*) implemented by Hummert *et al.* (*47*) in Python. The KV algorithm takes a minimal step size as a sole user input, limiting user bias. As our TCSPC modality records the arrival time of each photon, we can set the time binning of our data [*t*_bin_ (s)] after acquisition. Because of the inherent noise level and varying fluorophore brightness, a low threshold will count noise as events, overestimating the real number of fluorophores, whereas a high threshold will discard bleaching events, underestimating the real number of fluorophores. The threshold was chosen carefully to balance these two effects at 50 counts per *t*_bin_ of 5 ms, corresponding to 10 kHz at 1.36 μW. To compensate for variations in the laser power, the minimum step size was corrected for, according to

$$\text{Minimum step size}=50\frac{p_{485}}{1.36\;\mu W}$$ (14)

where *p*_485_ is the laser power of the 485-nm excitation laser for that measurement in microwatts (see also table S2).

## Supplementary Materials

### This PDF file includes:

Figs. S1 to S16 Tables S1 to S5 Supplementary Code Notes S1 to S5 References
