# Supplementary material for: Advanced multiparametric image spectroscopy and super-resolution microscopy reveal a minimal model of CD95 signal initiation
Source: Sci Adv. 2024 Aug 30;10(35):eadn3238. doi: 10.1126/sciadv.adn3238 (PMC11809610; doi:10.1126/sciadv.adn3238)
Supplement: Supplementary file 1 — Figs. S1 to S16 Tables S1 to S5 Supplementary Code Notes S1 to S5 References [file sciadv.adn3238_sm.pdf]

Science Advances
AAAS

Supplementary Materials for

Advanced multiparametric image spectroscopy and super-resolution microscopy reveal a minimal model of CD95 signal initiation

Nina Bartels *et al.*

Corresponding author: Cornelia Monzel, cornelia.monzel@hhu.de; Claus A. M. Seidel, cseidel@hhu.de

Sci. Adv. **10**, eadn3238 (2024)
DOI: 10.1126/sciadv.adn3238

This PDF file includes:

Figs. S1 to S16
Tables S1 to S5
Supplementary Code
Notes S1 to S5
References

Supplementary Figures

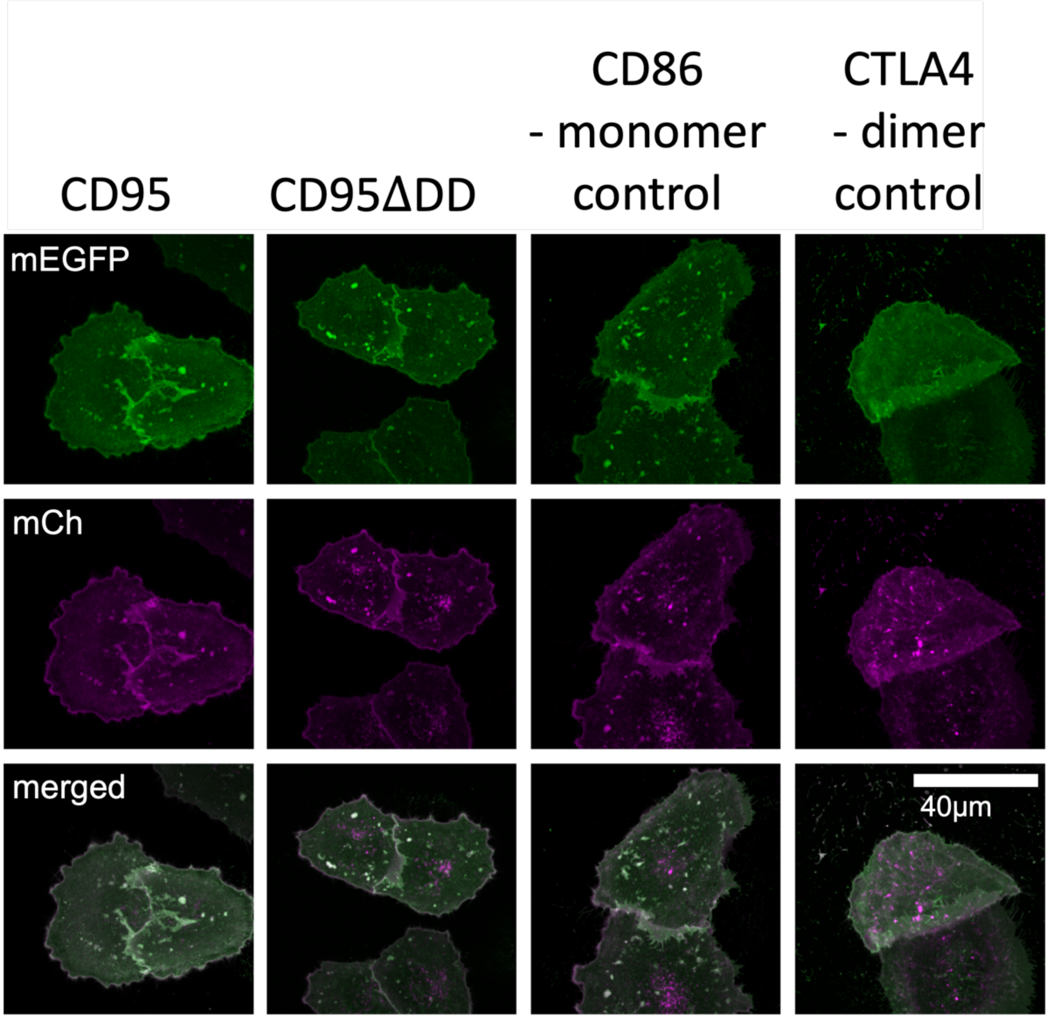

Fig. S1. Confocal images of transfected cells confirm protein localization to the plasma membrane.

Images show live Hela CD95KO cells transfected with bicistronic plasmids coding for donor (mEGFP) and acceptor (mCherry) fused to CD95, CD95(ΔDD), CD86 or CTLA4 during FRET measurements. Focus is set to the lower cell membrane. Higher intensities at cell edges and cell-to-cell contacts indicate the correct localization of the membrane proteins to the plasma cell membrane. Scale bar applies to all images. Exemplary cells shown from CELFIS dataset.

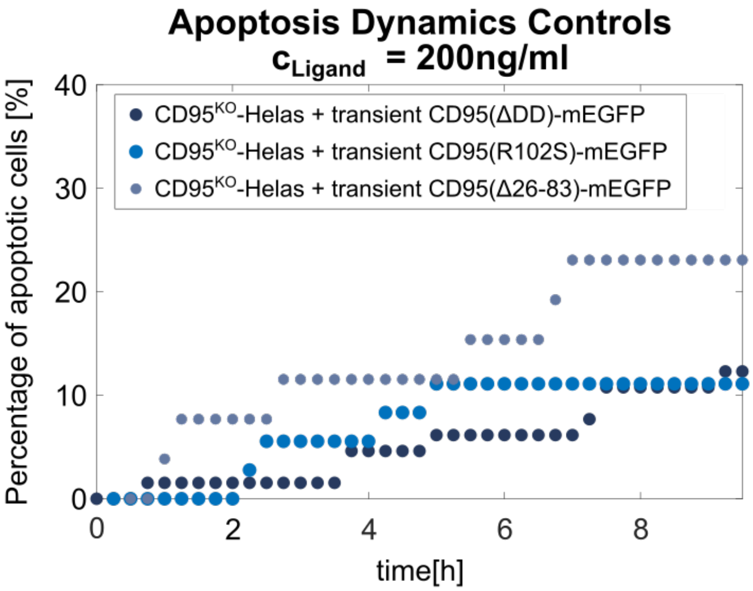

Fig. S2. Apoptosis dynamics of CD95 variants.

Apoptosis dynamics of transiently transfected Hela CD95KO cells with CD95 variants CD95(ΔDD), CD95(R102S) and CD95(Δ26-86). While the first two variants show apoptosis caused by natural apoptosis or transfection stress, the pre-ligand assembly domain PLAD depleted variant CD95(Δ26-86) (also called CD95(ΔPLAD)) shows an increased apoptosis efficiency up to 25% of dead cells. Statistics: > 25 cells for CD95(R102S) and CD95(Δ26-86), > 65 cells for CD95(ΔDD) from one measurement.

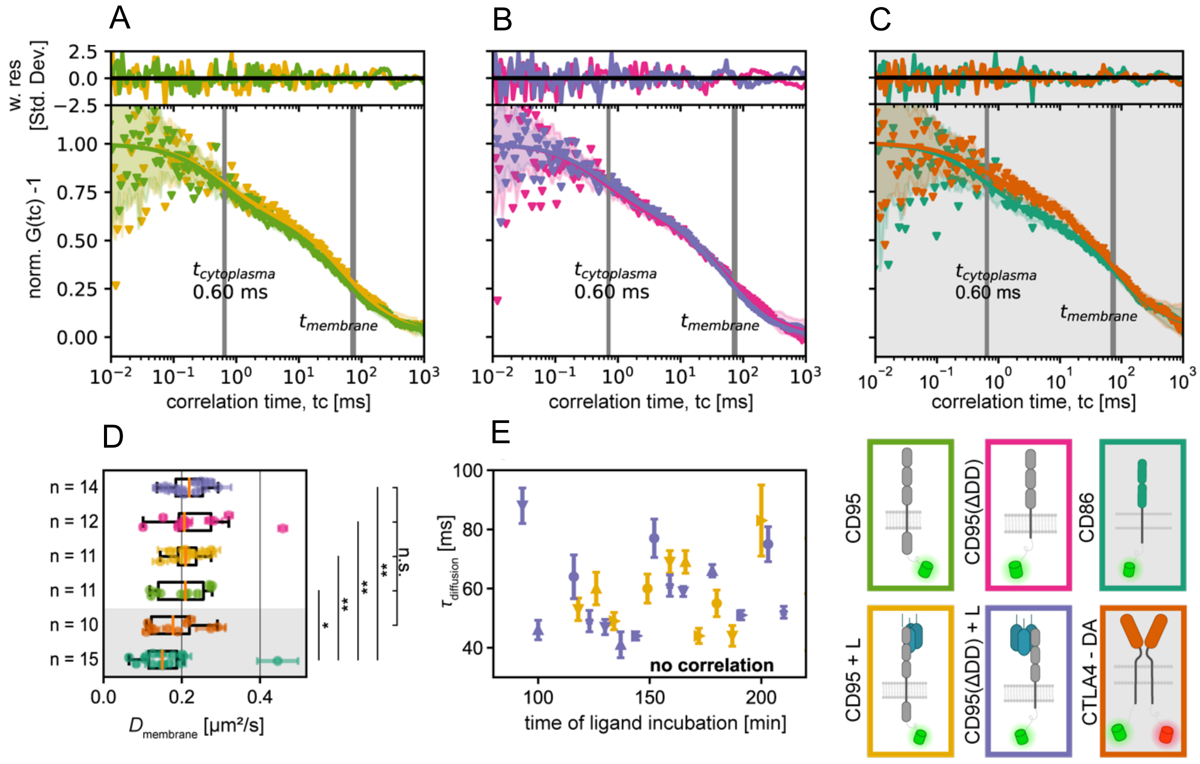

Fig. S3. Live cell FCS to obtain diffusion times.

Exemplary curves are shown for **(A)** CD95 before (green) and 159 minutes after ligand addition (yellow) **(B)** CD95(ΔDD) before (magenta) and 178 minutes after ligand addition (purple) **(C)** CD86 monomeric control (teal) and CTLA4 dimer control (orange). All curves were fitted with two diffusion terms (compare Methods and Equation (2)). The cytoplasmic diffusion term was fitted globally over 11 curves for the CD95 sample and fixed to this value for all other samples (see Methods). **(D)** Membrane diffusion constants were obtained from the membrane diffusion times. Two-sided Mann-Whitney U-test was used to test for significance (*$p < 0.05$, **$p < 0.01$). **(E)** Membrane diffusion time plotted against time after ligand addition. No significant change was observed. At least ten different positions from at least 7 different cells were measured per sample and fitted subsequently. The CD86 sample was recorded on two experiments on two measurement days, all other samples were recorded in a single experiment. Error bars of each data point indicate the fitting error. Legend: schematic representation of receptors. Cytoplasmic and membrane fractions reported in Table S4**.**

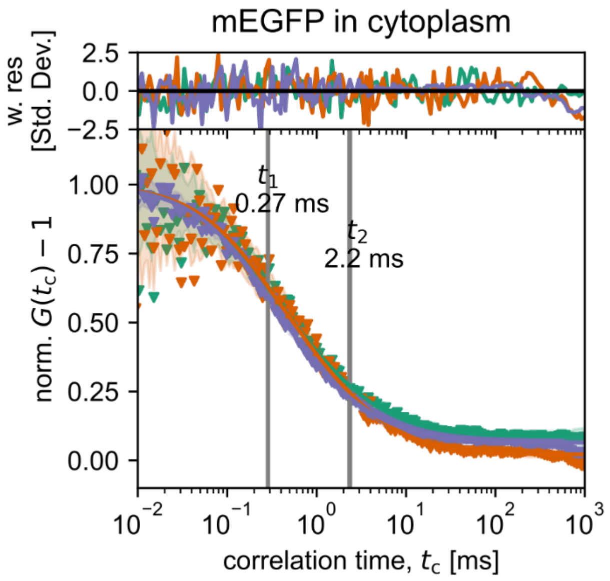

Fig. S4. FCS curves of free mEGFP in cytoplasm.

Free mEGFP in cytoplasm was fitted globally with two diffusion terms (Equation (2)), with a weighted average of 0.5 ms. Three datapoints recorded during a single experiment.

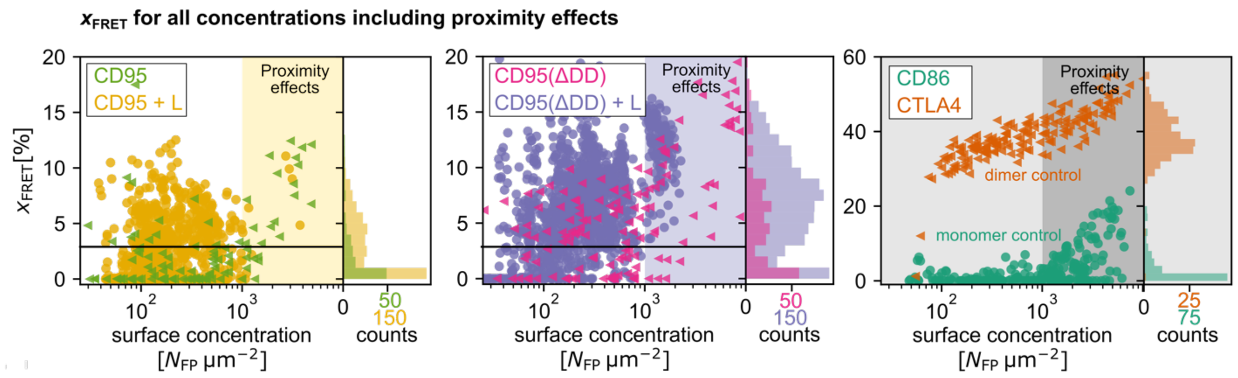

Fig. S5. FRET fraction including proximity effects at high concentrations.

x_{FRET} scatter plots and histograms as a function of receptor surface density (see Figure 3D) extended up to receptor surface concentrations of 10^4 receptors/µm². For CD86, the majority of $x_{FRET} \sim 0\%$ values (average value of $x_{FRET} \sim 3\%$) confirm the monomeric character of the control. CTLA4, quickly saturates around $x_{FRET} \sim 37\%$ indicating the dimer value this control. x_{FRET} of CD95 before CD95L incubation indicate primarily monomeric ($\geq 96\%$) and some dimeric ($\leq 4\%$) receptors. In case of CD95(ΔDD) percentages were slighlty different with ($\geq$ 88%) monomeric and some dimeric ($\leq 12\%$) receptors. After CD95L incubation ~15% of CD95 receptors oligomerize to dimers or trimers. In case of CD95(ΔDD), a slightly higher fraction of ~21% of receptors was found oligomerizing to dimers or trimers. We attribute the higher fraction in case of CD95(ΔDD) to the fact, that receptors can oligomerize over long time scales or to a missing steric hindrance due to the absence of the DD. Black horizontal line indicates the average $x_{FRET} \sim 3\%$ value of CD86 data. N > 108 cells from at least 4 independent experiments per condition. At concentrations > 1000 receptors/µm², a systematic increase in FRET in all samples is found. The monomeric CD86 control is used to determine the onset of proximity FRET. All data above the 1000 receptors/µm² threshold was not considered in the oligomerization analysis.

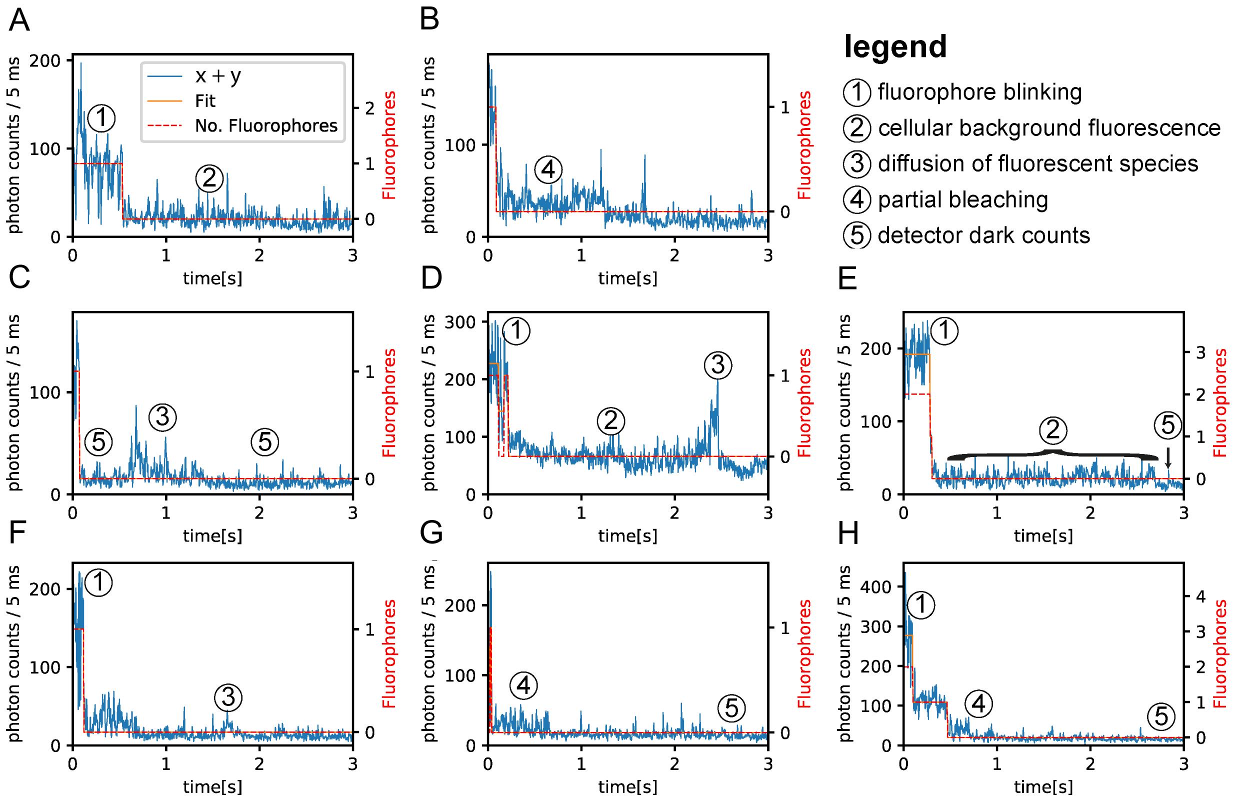

Fig. S6. Exemplary traces for cPBSA.

(A) – (H) Fluorescent signal during bleaching and fitted step traces. Sources of noise are labeled in each graph for illustration purposes. **(H)** Two-step bleaching event show variation in step size. Exemplary traces illustrate the overall data quality and noise sources.

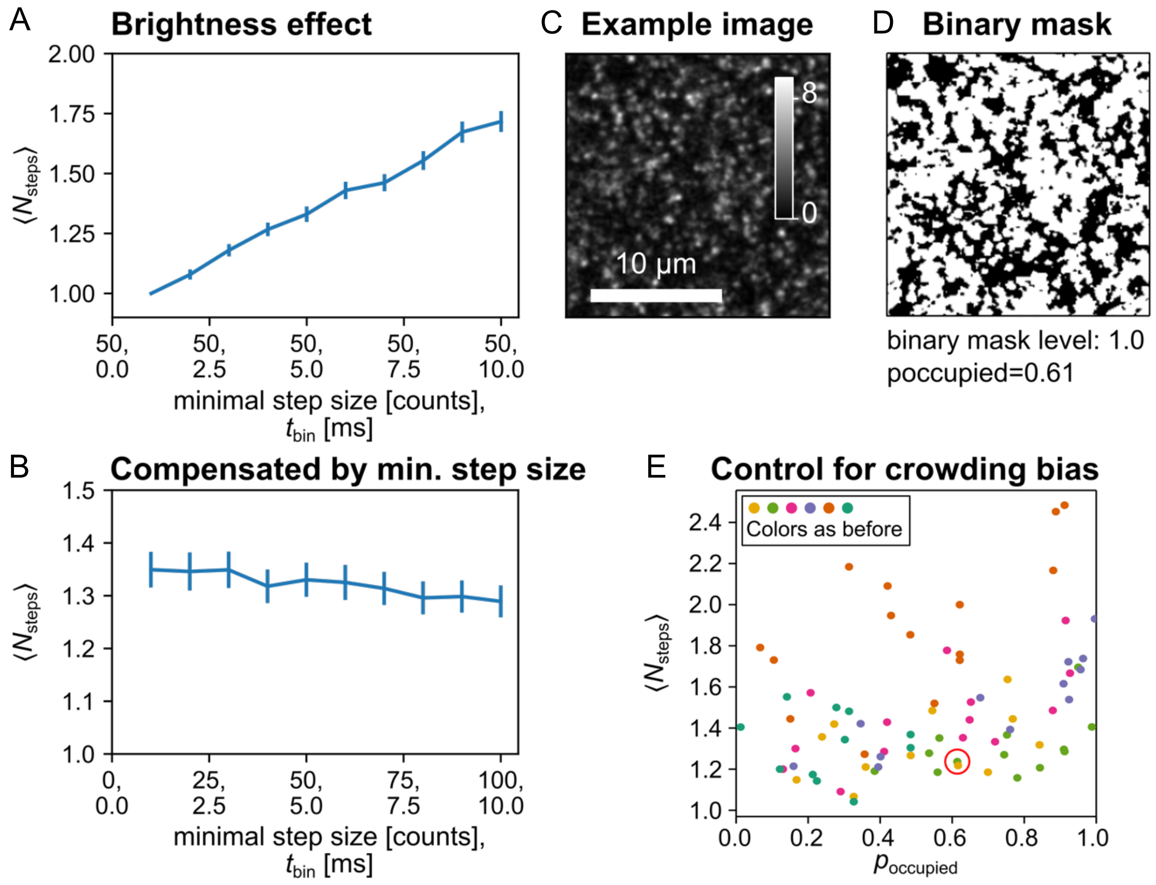

Fig. S7. Controls for confocal Photobleaching Step Analysis.

(A) Effect of the brightness (corresponding to the minimal step size and effectively changed via t_{bin}) on cPBSA analysis of the average number of fluorophores $\langle N_{\mathrm{steps}} \rangle$ for a CD86 experiment. **(B)** Effect disappears when the threshold is increased in the same proportion as t_{bin}. Thus a brightness independent determination of $\langle N_{\mathrm{steps}} \rangle$ is obtained. Data from same dataset as in (A). **(C)** Exemplary overview image from CD95 sample smoothed with a 1 pixel sigma Gaussian filter. **(D)** Corresponding binary image illustrating the spot occupancy probability p_{occupied} as an indicator of multi-molecular events due to crowding. p_{occupied} is determined as area fraction exceeding a signal intensity threshold of 1 photon. **(E)** To test how $\langle N_{\mathrm{steps}} \rangle$ depends on molecular crowding within the confocal volume, $\langle N_{\mathrm{steps}} \rangle$ of one area is plotted against the occupany probability p_{occupied} for the same area. A weak positive correlation between $\langle N_{\mathrm{steps}} \rangle$ and p_{occupied} is detected, supporting that surface concentration fluctuations are present in the sample. As the spread over p_{occupied} was similar for all samples, no additional correction for different p_{occupied} levels between samples had to be introduced. Color code as in main text. Red circle indicates data to the area shown in (C) - (D).

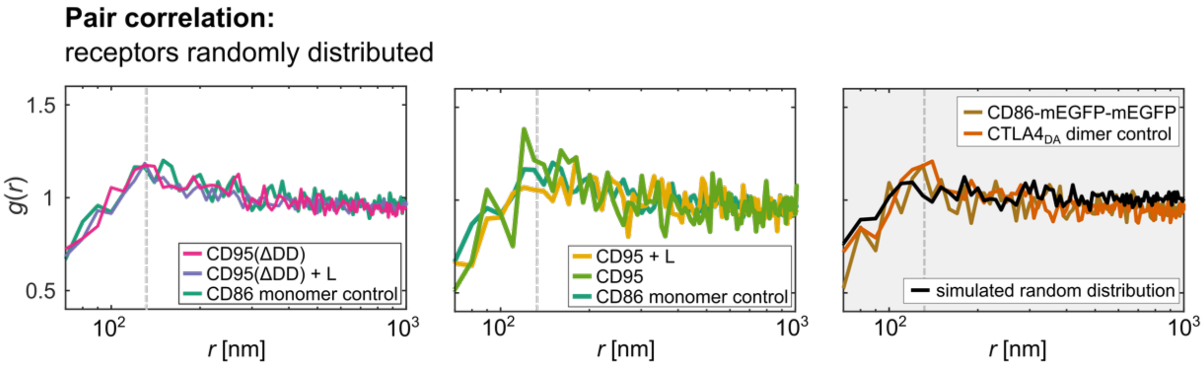

Fig. S8. STED pair correlation analysis of CD95 variants.

Pair correlation function $g(r)$ of fluorescent spots (Equation (8) in Methods) for CD95 variants and control receptors reveal a random distribution: distances $r > 130$ nm (right of dashed line) with $g(r) \approx 1$ indicate a random distribution. A decrease in correlation for $r < 130$ nm arises from PSF size effects and are not due to a particular distribution. This was verified by simulations of randomly distributed spots (black curve in right graph, see Methods).

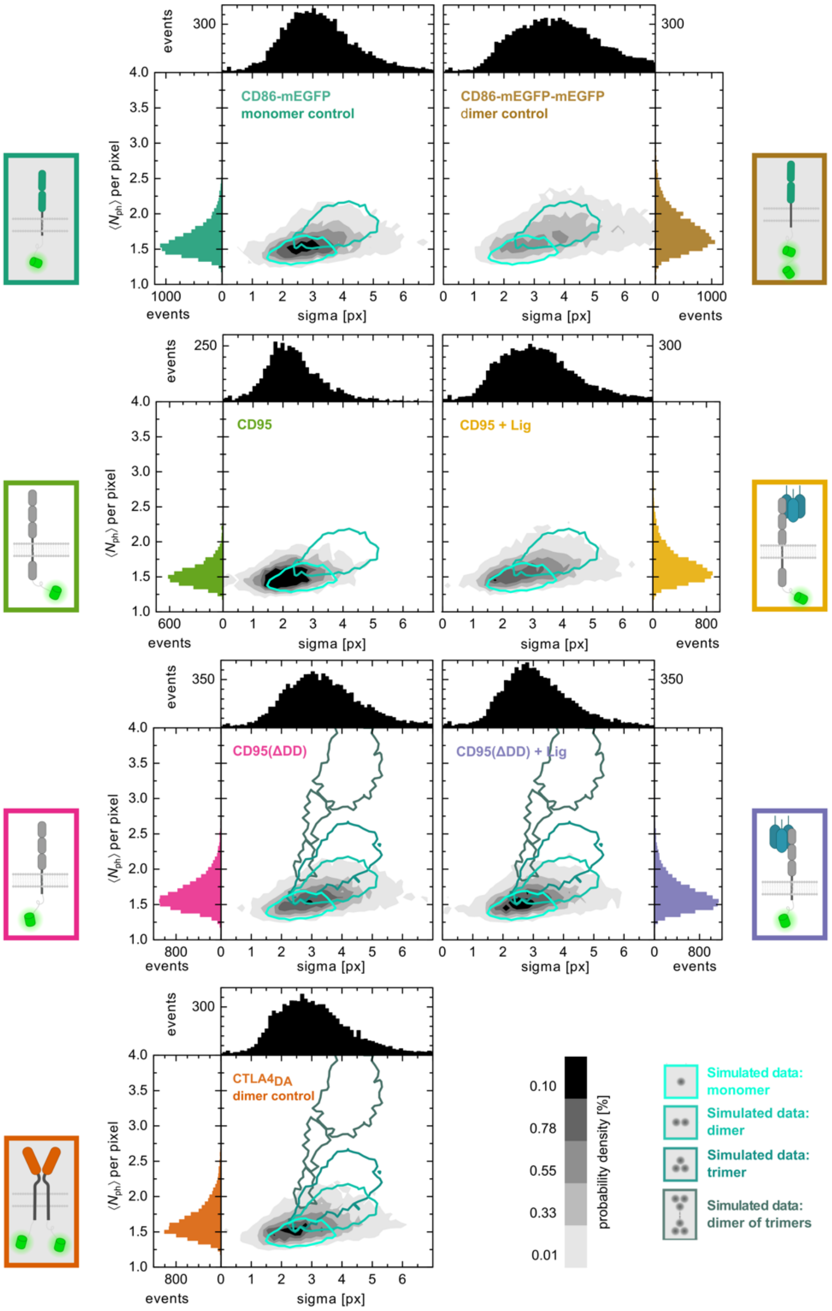

Fig. S9. STED spot analysis of CD95 variant.

Two-dimensional probability density representation of $\langle N_{\mathrm{Ph}} \rangle$ per pixel and σ values derived from individual spot analysis. Frequency histograms of $\langle N_{\mathrm{Ph}} \rangle$ per pixel and σ are depicted on the side and top of each graph. Measured $\langle N_{\mathrm{Ph}} \rangle$ per pixel and σ shown for monomer, pseudodimer control, CD95 and CD95(ΔDD) before and after CD95L incubation, as well as CTLA4. From simulations of pure monomer, dimer, trimer and dimer-trimer samples isolines enclosing 50% of data points

are calculated and depicted in the different panels for data comparison (teal lines). N > 5000 objects per sample.

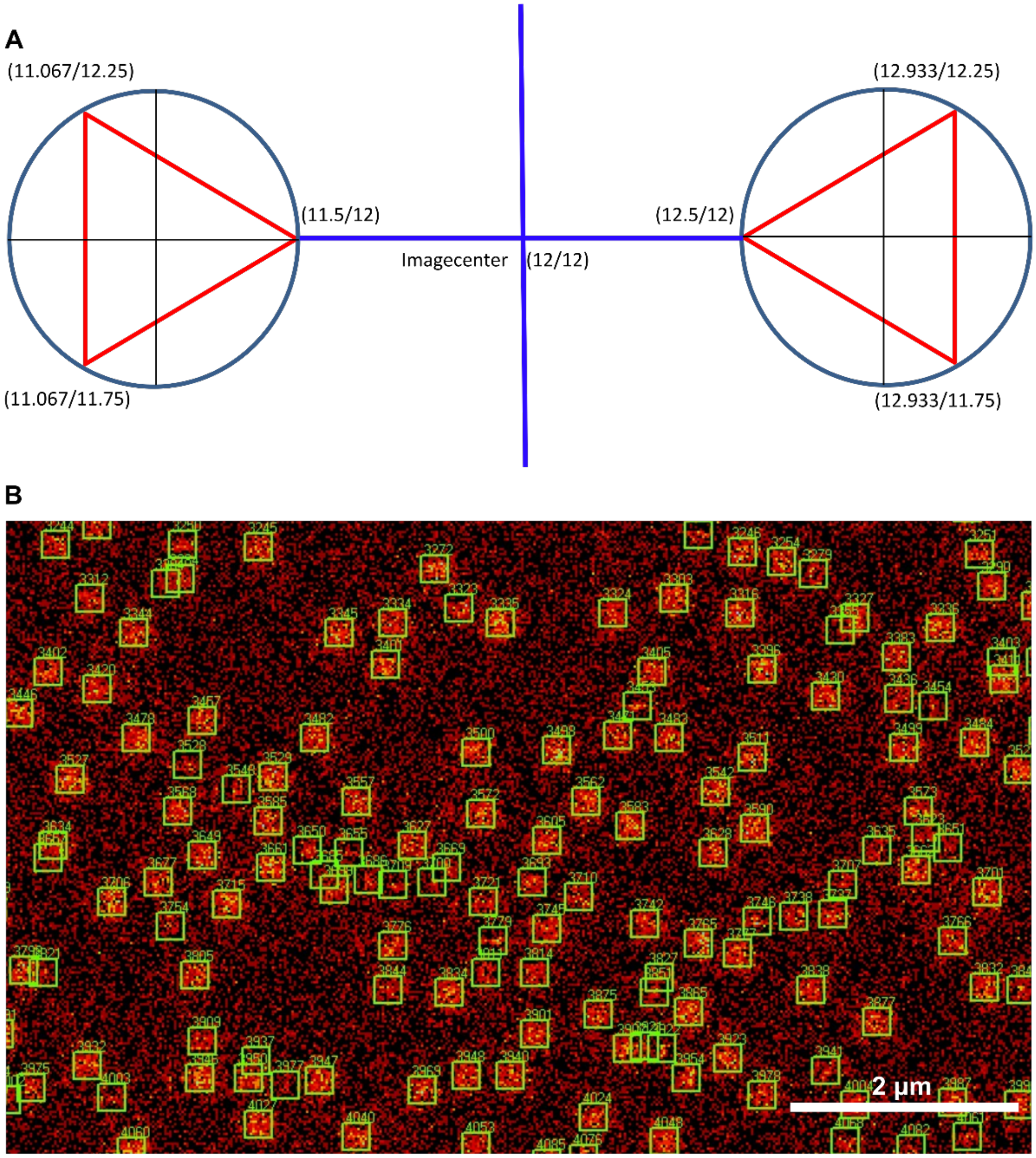

Fig. S10. STED spot simulations.

(A) Simulated dye positions ((x,y) in pixels) of a dimer-trimer in an 25x25 pixel image. For monomer, dimer and trimer simulations, one, two or three replicates of the left triangle were used, respectively. **(B)** Zoom of simulated trimer composite image. Simulated random distributions of fluorescent spots took into consideration brightness fluctuations and molecular crowding effects as derived from the monomer and dimer control measurments. For details on the simulation see Methods.

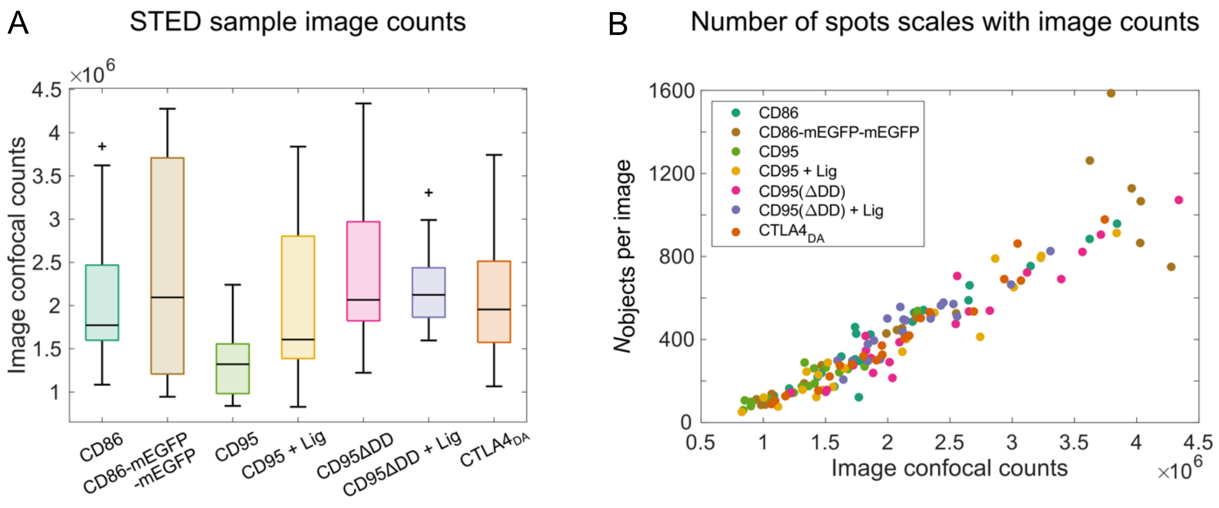

Fig. S11. STED sample expression level.

(A) The total counts per STED image are evaluated and displayed for all measured samples. N = 20 images per sample. **(B)** The number of detected spots per image are plotted versus image confocal counts. A near linear relation is found as expected. Receptor expression levels between samples are comparable, with slight deviations to a lower expression level in case of CD95 and a slightly higher expression level in case of CD86-mEGFP-mEGFP.

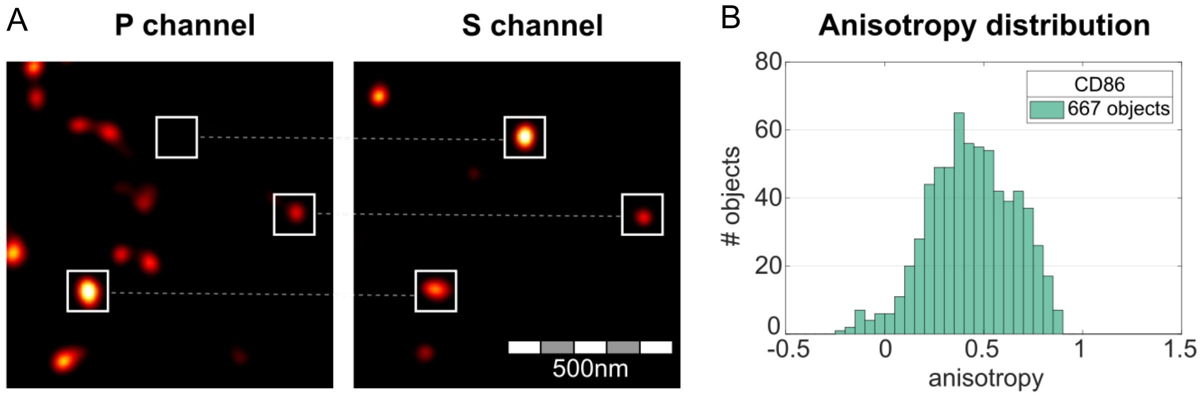

Fig. S12. Fluorescence anisotropy analysis of STED samples.

(A) Deconvolved STED images of Hela CD95^{KO} CD86-mEGFP stained with Atto647N α-GFP nanobody in parallel (P) and perpendicular (S) channel. The comparison of both images shows, that the emitted light is not equally distributed to both channels. The scale bar applies to both images. **(B)** The histogram shows the measured anisotropy of n = 667 objects, i.e. STED resolution limited spots (see Methods and Equation (7) for details.). The spread in anisotropy confirms a strong polarization effect, signifying a broad distribution of fluorescent protein orientations in the fixed membrane.

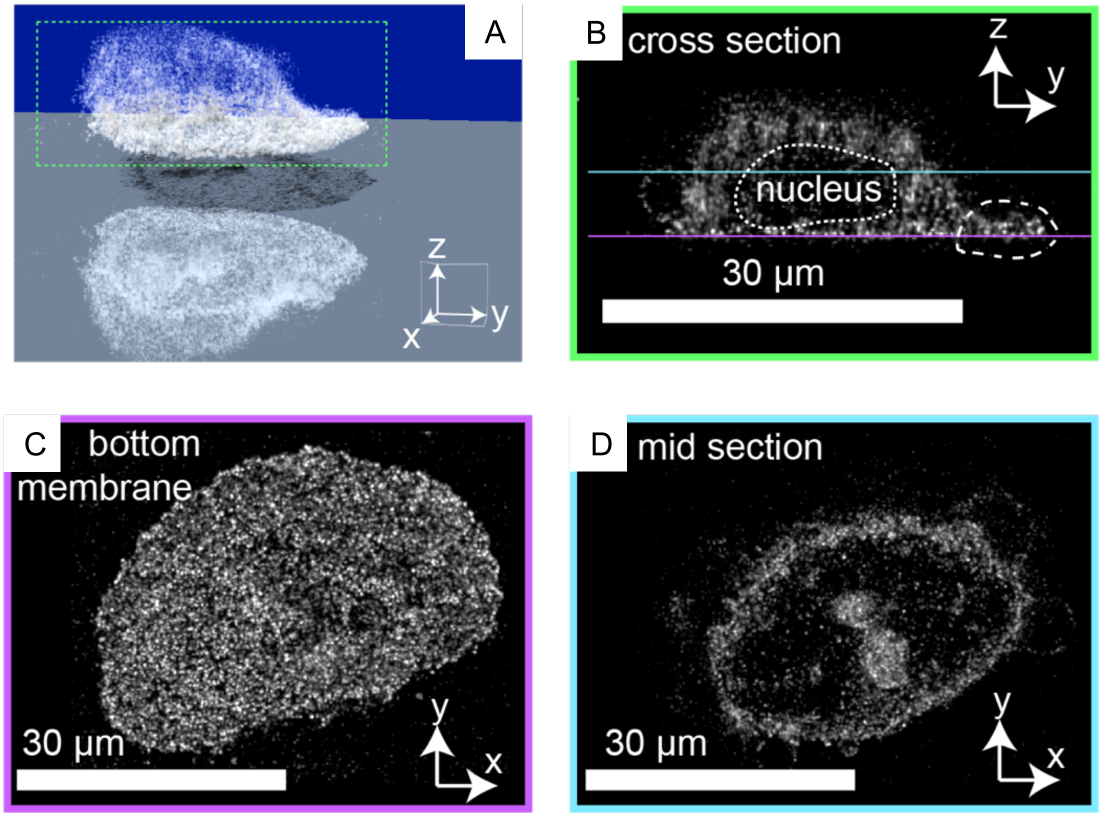

Fig. S13. 3D confocal image of CD95 transfected fixed cell.

(A) 3D fluorescence data of cell and projections. Cell was weakly expressing mEGFP. **(B)** yz cross section as indicated by dashed box in (A). Dashed line indicates the area where two membranes are in close proximity. Contour of nucleus is also shown by dotted line. **(C)-(D)** bottom and mid sections corresponding to colored planes in (B). The projections highlight the need to measure cPBSA data below the nucleus to prevent having two membranes within the confocal volume. Exemplary cell recorded during a single experiment.

Supplementary Tables

plasmid id	88	88	1649	1516	1531	1706	1693	1695	1714
sequence	*CD86- link-mEGFP-link-mEGFP*[3]	*CD86-link-mEGFP-link-mEGFP*[3]	*CD86-mEGFP*	*CD95-ΔDD(1-210)-mEGFP*	*CD95(1-335)-mEGFP*	*CD86-mCherry T2A CD86-mEGFP*	*CTLA4(1-200)-mCherry T2A CTLA4(1-200)-mEGFP*	*CD95-ΔDD(1-210)-mCherry T2A CD95-ΔDD(1-210)-mEGFP*	*CD95(1-335)-mCherry T2A CD95(1-335)-mEGFP*
linker sequence	GSSGSSNAAIINAAGSSGSS	GGGPVPQWEGFAALLATPVGGAV	GGGPVPQWEGFAALLATPVGGAV	GGGPVPQWEGFAALLATPVGGAV	GGGGGPVPQWEGFAALLATPVGGAV	GGGPVPQWEGFAALLATPVAT / GGGPVPQWEGFAALLATPVGGAV	GGGPVPQWEGFAALLATPVAT / GGGPVPQWEGFAALLATPVGGAV	GGGPVPQWEGFAALLATPVAT / GGGPVPQWEGFAALLATPVGGAV	GGGGGPVPQWEGFAALLATPVAT/ GGGGGPVPQWEGFAALLATPVGGAV
flexible part protein #aa	11	-[2]	-[2]	16	9[1]	-[2]	16[4]	16[4]	9[1]
linker length #aa	20	23	23	23	25	21 / 23	21 / 23	21 / 23	23 / 25
flexible part FP #aa	12	12	12	12	12	16 / 12	16 / 12	16 / 12	16 / 12
Total flexible #aa	43	35	35	51	46	37 / 35	53 / 51	53 / 51	48 / 46

Table S1. Specifications of linker lengths for used constructs.

#aa: number of amino acids. *$link$ indicates the position of the linker detailed. †flexible part could not be estimated because the structure of the CD86 transmembrane TM domain is not known, taken as 0. ‡9 amino acids are used to model the flexible death domain, see methods. §linker length based on the residual 16 intracellular amino acids after transmembrane domain. CTLA4-mEGFP was not used as it did not localize to the membrane.

Dataset	Date	Laser power [µW]	Laser power density [kW/cm^2]	Minimum step size [counts]
**CD95**	22.07.2021	1.36	0.82	50
**CD95 +L**	23.07.2021	1.60	0.97	58
**CD95(ΔDD)**	22.07.2021	1.36	0.82	50
**CD95(ΔDD) +L**	23.07.2021	1.60	0.97	58
**CD86-mEGFP$_1$**	22.07.2021	1.36	0.82	50
**CD86-mEGFP$_2$**	24.11.2021	1.37	0.83	50
**CTLA4$_{DA}$**	02.02.2022	1.00	0.61	36
**CD86-mEGFP-mEGFP**	02.02.2022	1.00	0.61	36

Table S2. Laser power changes between measurement days in case of PBSA.

Since fluorescent brightness varied between measurement days (due to different mEGFP orientations or due to slight laser power changes), we tested how brightness changes affect the minimal step size, a key factor for the assignment of bleaching steps. By changing the bin time, which is analogous to changing the laser power, we found a scaling behaviour of the number of steps N_{steps}. The step threshold was adjusted such that the ratio of the power and the threshold remains constant (see Fig. S7. A,B).

Parameter name	Monomer	Dimer	Trimer	Dimer of Trimers
**Intensity of 2D Gaussians (Dye Brightness)**	Left triangle<br>1.1 +- 30% [ph]	Left triangle<br>0.8 +- 30% [ph]<br>0.8 +- 30% [ph]	Left triangle<br>0.8+- 30% [ph]<br>0.8+- 30% [ph]<br>0.8+- 30% [ph]	Left triangle<br>0.8 +- 30% [ph]<br>0.8 +- 30% [ph]<br>0.8 +- 30% [ph]<br><br>Right triangle<br>0.8 +- 30% [ph]<br>0.8 +- 30% [ph]<br>0.8 +- 30% [ph]
**Poisson noise**	yes	yes	yes	yes
**Dye position (X / Y) coordinates in 25x25 image**	Left triangle<br>(11.500 / 12.000)	Left triangle<br>(11.500 / 12.000)<br>(11.067 / 11.750)	Left triangle<br>(11.500 / 12.000)<br>(11.067 / 11.750)<br>(11.067 / 12.250)	Left triangle<br>(11.500 / 12.000)<br>(11.067 / 11.750)<br>(11.067 / 12.250)<br><br>Right triangle<br>(12.500 / 12.000)<br>(12.933 / 11.750)<br>(12.933 / 12.250)
**Sigma_X = Sigma_Y of 2D Gaussian**	Left triangle<br>2.8 [pixel]	Left triangle<br>4.4 [pixel]<br>4.4 [pixel]	Left triangle<br>4.4 [pixel]<br>4.4 [pixel]<br>4.4 [pixel]	Left triangle<br>4.4 [pixel]<br>4.4 [pixel]<br>4.4 [pixel]<br><br>Right triangle<br>4.4 [pixel]<br>4.4 [pixel]<br>4.4 [pixel]
**Mean of Poisson background noise**	0.4 [ph]	0.4 [ph]	0.4 [ph]	0.4 [ph]
**Composite image size**	2500 x 2500	2500 x 2500	2500 x 2500	2500 x 2500
**Total number of generated spots**	10 000	10 000	10 000	10 000

Table S3. Parameters used for STED image spot simulations.

Sample	Statistics [cells]	Membrane fraction [%]	Cytoplasmic fraction [%]
$CD86_{DO}$	9	0.589 ± 0.085	0.411 ± 0.085
$CD86_{DO}$	6	0.628 ± 0.052	0.372 ± 0.052
$CTLA4_{DA}$	10	0.703 ± 0.036	0.297 ± 0.036
$CD95_{D0}$	11	0.594 ± 0.045	0.406 ± 0.045
$CD95(\Delta DD)_{D0}$	12	0.598 ± 0.052	0.402 ± 0.052
$CD95_{D0}$ + Lig	11	0.597 ± 0.043	0.403 ± 0.043
$CD95(\Delta DD)_{D0}$ + Lig	14	0.637 ± 0.030	0.363 ± 0.030
Total	73	0.621 ± 0.062	0.379 ± 0.062

Table S4. Membrane and cytoplasmic molecular fraction determined with FCS.

Fractions of fast cytoplasmic and slow membrane diffusion for different membrane proteins measured with FCS. See Supplementary Note 1 and Fig. S3**.**

Supplementary Codes

```
#all units in s, 1/s
#this code is fast in c, but slow in python
def simulateTrace(Nfl, alpha, n, tbin = 5e-3, tstep = 1e-4, timestop = 1):
    """
        Do a Monte Carlo simulation of a trace, assuming a single dark state.
        tstep is the time resolution of the simulation. It should be chosen such
    that the probability of multiple blinking events in 1 step is low, i.e.,
    pon and poff < 0.1
    Nfl:      average number of fluorophores in time tbin if the molecule is on,
              i.e. the molecule brightness (dimensionless)
    alpha:    the average fraction of time the molecule spends in the on state (dimensionless)
    n:        the average number of blinks in time tbin (dimensionless)
    tbin:     time period (s)
    tstep:    time resolution of simulation, see above (s)
    timestop: amount of time to simulate (s)"""
    #calculate derived variables
    Nevents = int(np.ceil(timestop / tstep))
    pfl = Nfl / tbin * tstep
    pon = n * alpha / tbin * tstep
    poff = n * (1-alpha) / tbin * tstep
    #fluorophore starts in the on state, compliant with physical conditions.
    state = 'on'
    #initialize arrays
    events = np.zeros(Nevents)
    #event loop
    for i in range(Nevents):
        if state == 'on':
            #add a poissonian number of photons
            events[i] = np.random.poisson(pfl)
            #switch off with probability poff
            if poff > np.random.random():
                state = 'off'
        elif state == 'off':
            #switch on with probability pon
            if pon > np.random.random():
                state = 'on'
    #downsample trace to tbin
    binfact = int(np.ceil(tbin / tstep))
    nbins = int(np.ceil(timestop / tbin))
    trace = np.sum(events.reshape((nbins, binfact)), axis = 1)
    #calculate variance
    variance = np.var(trace)
    return variance, trace
```

Supplementary Code 1: Code for Monte Carlo simulations on variance predictions.
Dark state Monte Carlo simulations for PBSA trace segment variance prediction. Code was tested in python 3.7, but should also run in Python 2.x and 3.x versions. The only dependency is the numpy library.

Supplementary Text

Supplementary Note 1: Optimal instrument settings for live cell FCS

For method details see method section *FCS measurements*.

Since fluorescence correlation spectroscopy (FCS) measurements are more sophisticated in live-cells, due to the natural variability and signal contributions of the cytoplasm and plasma membranes, we worked out optimized settings for laser power, pinhole and recording time to optimally balance signal-to-noise gains with recordings of less stable fluorophores, such as monomeric enhanced green fluorescent protein (mEGFP) (see Methods and Supplementary Notes 1&2).

Live cell FCS measurements on mEGFP remain challenging due to the limited mEGFP photostability and limited mEGFP abundance in a cell. To nevertheless obtain a robust readout, we discuss optimal experimental settings along with a brief description of the photophysical effects governing the observations.

Pinhole setting

The optimal pinhole setting was determined experimentally to be 200 µm in diameter, or 492 nm backprojected pinhole radius (*77*), which corresponds to 2.1 airy units (AU). This setting optimally balances 1) a high photon collection efficiency 2) a sharp point spread function (PSF) 3) the PSF shape to resemble a Gaussian. The tradeoff consists thereof that an open pinhole with high collection efficiency is needed to compensate for the poor photo-stability of mEGFP and resulting low signal-to-noise ratio (SNR). However, opening the pinhole transforms the shape of the PSF from a $sinc^2$, which is Gaussian-like to a sinc function, which in not Gaussian-like. As FCS theory (see Equation (4)) models a molecule diffusing through a 3D Gaussian volume, an open pinhole results in a mismatch between model and measurement visible in the fit residuals. As reported by others (*71*), a 2.1 AU pinhole leads to small but acceptable deviations between the model function and data.

Fluorescent molecule concentration changes during measurement

A change in fluorescent protein concentration is registered by the correlation function at long time scales, which complicates fitting slow membrane diffusion. For solution measurements, the dominant process for concentration decrease is adsorption of to the glass surface, which is easily prevented by coating the glass surfaces with bovine serum albumin (BSA) (incubate 1 mg/ml BSA for 10 minutes, BSA, Sigma-Aldrich Merck group, Taufkirchen, Germany). Bleaching does not significantly affect concentrations in solution measurements as the bleaching rate is small compared to the large fluorophore reservoir. In cells, a change in fluorescent protein concentration cannot be circumvented as photo-bleaching can readily deplete the reservoir of fluorescent proteins at an organelle or cellular scale. To mitigate the effects of a decreasing mEGFP concentration on the FCS curve, we divide the photon trace in chunks of approximately constant concentration and average the pieces (*71*).

We are able to gain additional insight in the photo-bleaching process from synergistically combining our read-out from FCS and cPBSA. From our FCS measurements we obtain diffusion times and fluorophore brightness, which we use to calculate the average number of photons per time the molecule diffuses through the focus to be ~1.5 for mEGFP. From cPBSA we are able to obtain the total photon budget of mEGFP to be ~1000 photons. Taken together we conclude that the probability of mEGFP bleaching during a single pass through the detection volume is very low

and that the mEGFP concentration decreases because a single molecule passes through the detection volume many times.

Power setting & photon budget

A higher laser power increases the signal-to-noise ratio for the FCS curve at the cost of a higher bleaching rate, which cause unwanted changes in local concentrations (see section above). In this section, we explain the underlying processes and obtain a trade-off between the SNR level of the FCS curve and the bleaching rate.

Primarily, the SNR of an FCS curve must be sufficient to enable interpretation, which scales with the number of photons detected while a single molecule diffuses through the focus. Interestingly, our results indicate that the average number of photons is ~1.5. On the condition that molecules diffuse independent from each other, at least two photons are needed to obtain a correlation. This apparent contradiction is resolved by realizing that the number of photons per event follows a distribution with a long tail at higher photon numbers. I.e., while some of the molecules emit zero or one photon, the fraction which emits two or more photons is responsible for the correlation in FCS.

To help understand bleaching processes, we introduce the concept of photon budget to mean the total amount of photons emitted by the fluorophore before bleaching. It is inversely proportional to the bleaching probability per excitation cycle. Work done by others (*78*) on decay pathway modelling reveals that the photon budget of mEGFP is constant at low irradiance but decreases after a transition regime. The decrease in photon budget is due to an additional photon being absorbed while the molecule is in the excited state, opening up additional photo-bleaching pathways and increasing the photo-bleaching probability per cycle. While a laser power lower than the transition irradiance maximizes the photon budget, a definite number was not found in literature by the authors, although an upper limit was reported by Cranfill et al. (*78*) to be 80 μW using 488 nm excitation in a diffraction limited focus. Based on our own experimental experience we estimate the transition point from mEGFP to be lower than ~10 μW.

To satisfy all the criteria above, the laser power was experimentally determined to be 5 μW corresponding to 3 kW/cm^2 for a calibrated 0.165 μm^2 focal area.

Recording time

Longer recording times improve the SNR of the FCS curve. However, to sample sufficient cell-to-cell variation during a measurement day it was limited to 5 minutes.

Supplementary Note 2: Live-cell Membrane FCS

To verify that cluster of differentiation 95 (CD95) is sufficiently mobile and hence able to form (higher) oligomers, we determined CD95 diffusion constants D during the whole signaling process using fluorescence correlation spectroscopy (FCS). FCS was performed on live cells for CD95 with donor in the absence of acceptor $CD95_{D0}$ (Fig. S3 A) and CD95 without death domain $CD95(\Delta DD)_{D0}$ (Fig. S3 B) before and 100 - 200 minutes after ligand addition as well as for cluster of differentiation 86 $CD86_{D0}$ and cytotoxic T-lymphocyte-associated protein 4 with donor in the presence of acceptor ($CTLA4_{DA}$) as single and double transmembrane helix references, respectively (Fig. S3 C). FCS curves were generated for each cell and fitted with two diffusion terms and no bunching term (see methods). The fast diffusion term was attributed to the presence of cytoplasmic mEGFP, which was confirmed by 3D confocal images of live cells. As the confocal detection volume extends halfway into the cytoplasm, FCS is sensitive to mEGFP present in the cytoplasm (see Fig. S3). To confirm that the fast diffusion component was of cytoplasmic origin, we fitted the fast diffusion term globally for all curves from the CD95 sample yielding a value of $t_{\text{diff,cp}} = 0.60$ ms (corresponding to $D = 20$ µm^2/s), reminiscent of soluble protein diffusion in eukaryotic cells, with typical values of 24 µm^2/s (*79*). In addition, we measured diffusion of free mEGFP in the cytoplasm and obtained two diffusion times, 0.27 ms and 2.2 ms, with a weighted average time of 0.50 ms (see Fig. S4) close to the cytoplasmic component of CD95. In the following, the fast diffusion term was kept fixed for all samples to improve the sensitivity of the fit for the slow diffusion time (see Fig. S3 A-C). The time of the slow diffusion process turned out to lie in the range of 30-100ms and matches literature values for membrane proteins. No difference in the diffusion constant was found between CD95 species within the measurement accuracy (see Fig. S3 D), with absolute values of $D = 0.21$ - 0.24 µm^2/s. Interestingly, $CTLA4_{DA}$ showed similar diffusion times of $D = 0.19$ µm^2/s, but CD86 showed significantly slower diffusion times of $D = 0.15$ µm^2/s indicating that the number of transmembrane helices is not the dominant factor of receptor diffusion. This result indicates that CD95 is sufficiently mobile to exhibit dynamic changes in its oligomeric state over time. In addition, comparison of the absolute values suggests that CD95 does not form supramolecular structures as this would result in a highly decreased diffusion constant. Finally, we tested whether the mobility of CD95 would change after ligand addition. To this end, CD95 and CD95(ΔDD) diffusion was monitored over 100-200 minutes after ligand addition (see Fig. S3 E). This measurement shows no systematic increase of the diffusion times after ligand addition exceeding the variability in diffusion times, which is seen by the scatter of data points and is with ~40-90ms relatively broad as expected for cell samples. Overall, our data confirm sustained CD95 mobility during the whole signaling process. Despite this possibility to accumulate into higher ordered structures CD95 did not show any systematic change in CD95 diffusion, thus indicating no excessive change in the receptor oligomerization state.

Supplementary Note 3: Calculation of FRET fraction correction factor

To calculate the percentage of oligomers present in the sample, one first has to note that our data relies on the analysis of the donor signal alone. From this signal, the oligomerization type and fraction is derived. It is further assumed that donor and acceptor labeled receptors behave identically, wherefore the results from the donor signal are extended towards the acceptor signal.

To calculate the FRET fraction correction factor, one needs to be aware, that homo- and heterodimers as well as dimers with one or two inactive mEGFP can form. The measured x_{FRET} hence underestimates the number of dimers which are present in the sample. To account for this effect, the probability to form hetero-dimers, p_{AD}, is calculated based on the donor abundance, their maturation, and assuming a binomial distribution. The abundance of donor to acceptor molecules was 1 : 3.5 for CTLA4, yielding a p_{AD} = 78% of hetero-dimers and for CD95 and CD86 the ratio was 1 : 2.5 yielding a p_{AD} = 71% of hetero-dimers (see calculation for CD95 in the following table).

Table S5. Exemplary calculation of the probability for hetero-dimer formation, p_{AD}, in CD95 experiments.

species	degeneracy	Donor fluorescence weighting	final weighting
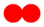	1x	0x	$0 \times p^2_{A,on}$
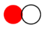	2x	0x	$0 \times p_{A,on}\, p_0$
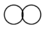	1x	0x	$0 \times p^2_0$
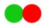	2x	1x	$2 \times p_{D,on}\, p_{A,on}$
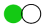	2x	1x	$2 \times p_{D,on}\, p_0$
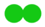	1x	2x	$2 \times p^2_{D,on}$

$p_{D,on} = 0.29{*}0.8$

$p_0 = 0.29{*}0.2$

$p_{A,on} = 0.71$

$p(F_D)$ = 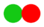 + 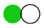 + 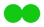

p_{AD} = (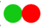) / $p(F_D)$

legend

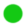 donor on

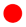 acceptor on

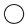 donor off

In our experimentes, the abundance of donor to acceptor molecules is 1 : 2.5, i.e. 29% donors and 71% acceptors. Furthermore, x_{FRET} is corrected for by a cloud correction factor ξ , which represents the probability that the two receptor coupled fluorophores and corresponding linkers are within a distance to perform FRET. Such value is obtained from accessible volume (AV) simulations. AV simulation were were performed in the program Olga (*44*) assuming a 51 amino acid linker and effective FRET range up to 82 Å using a solution NMR model of trimeric CD95 TM-domains (pdb id: 2NA7(*48*)) to set the anchor points for all structures. in this case amounted to $\xi = 0.465$.

The maximum measurable FRET fraction, $x_{\mathrm{FRET,max}}$, corresponding to a 100% dimeric sample, is hence calculated according to

$$\xi * p_{AD} * x_{\mathrm{FRET,max}}\,[\%] = 100\ \%\ \text{oligomers}$$

This corresponds more generally to

$$\xi * p_{AD} * x_{\mathrm{FRET}}\,[\%] = \text{oligomers}\,[\%]$$

which was 36% for 100% CTLA4 dimers (or 1% x_{FRET} ~ 2.8% oligomer fraction) and 33% for 100% CD95, CD95(ΔDD) and CD86 dimers (or 1% x_{FRET} ~ 3% oligomer fraction).

Supplementary Note 4: Advanced cPBSA analysis from trace counts

Since conventional photobleaching step analysis requires an optimized choice of the minimal step size, we set out to analyze the trace counts as a second readout to determine receptor stoichiometries within diffraction limited spots. We expected the number of bleaching steps and bleaching trace counts to directly scale with each other and verified this is the case. We report linear scaling laws of step numbers vs. counts/frame for monomers and oligomers detected in our samples. Thus, and most importantly, cPBSA determines average receptor stoichiometries within spots, it provides an upper limit of the stoichometric values, and allows to evaluate the impact of crowding.

For trace count based evaluation, we analyzed the image counts recorded at low excitation power prior to bleaching, as well as the counts detected from spots during bleaching. We verified that trace counts were recorded at power levels, where the probability of higher excited states is negligible.

Average photon traces detected during bleaching and normalised to unit amplitude are shown in Fig. S14 (right). Here, several features of the bleaching process are noticed: 1) The bleaching process is highly non-exponential indicating that the bleaching efficiency between spots of the same sample varies considerably. This variation could be caused by spatial changes in excitation power, the distribution of dye orientations, the blinking of dyes, amongst others. 2) Samples split into two populations with different initial rate of bleaching. The population with high efficiency of bleaching include CD86-mEGFP and C95 samples; CD86-mEGFP-mEGFP (pseudodimer) and $CTLA4_{DA}$ samples have a lower bleaching efficiency. The lower $\langle N_{steps} \rangle$ of $CTLA4_{DA}$ compared to CD86-mEGFP-mEGFP can be understood from the fact that $CTLA4_{DA}$ dimers consisted of donor-donor as well as of donor-acceptor pairs. The more suitable CTLA4 donor only ($CTLA4_{D0}$) expression was not possible, since the plasmid did not localize to the membrane correctly. 3) At longer time scales, bleaching decays have similar power-like time-dependency separated in macrotime by a factor of ~ 5. From the observed features we can suppose that there are at least two different processes defining the bleaching: one is the average bleaching efficiency, which is different for different samples. Another is a slower process, which is common for all samples. This slower process can be related to the dye properties, such as the number of cycles the dye is excited before it gets bleached. To separate the fast bleaching effect, bleaching count traces were conditioned by cutting off detected counts after a macrotime-threshold and below an intensity threshold. The macrotime cutoffs were defined as the time point at which the bleaching trace reached the lowest intensity level (corresponding to the local background (BG) intensity). The distribution of determined macrotime cutoffs is shown in Fig. S14 (left) and the sample average time-cutoffs are indicated at Fig. S14 (right) as vertical lines. As can be seen, the average cutoffs are grouped in samples of high and low bleaching efficiency and average time-cutoffs scale by a factor ~ 5 also here. The intensity threshold, which is effectively a background (BG) threshold was set to ~ 20% of the maximum count rate. The resulting sample average BG thresholds are indicated in Fig. S14 (right) as horizontal lines.

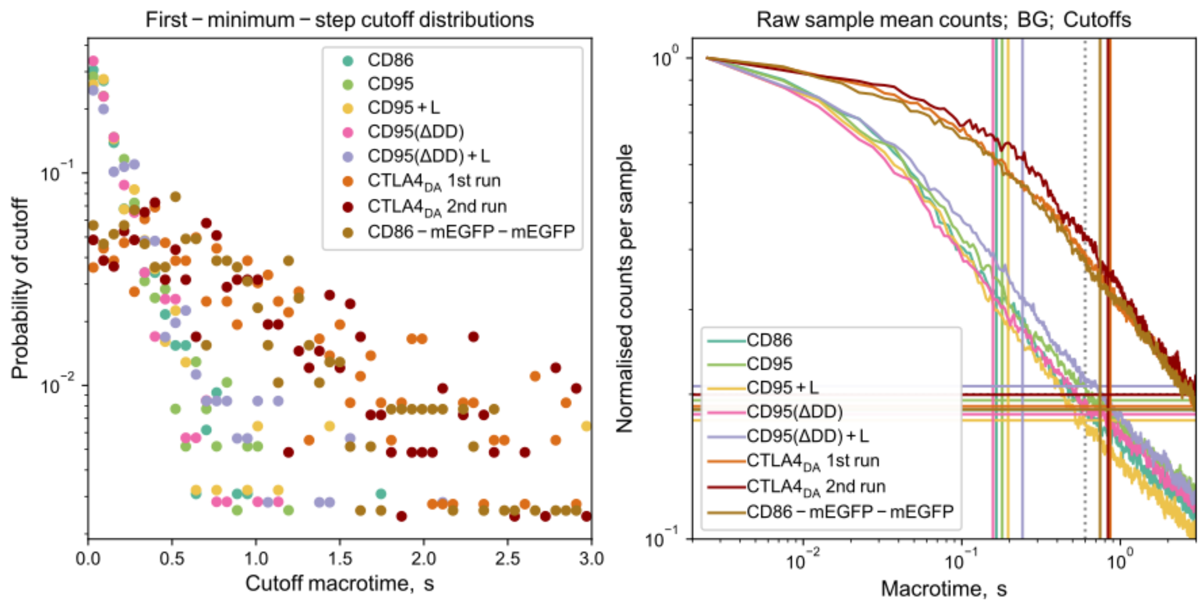

Fig. S14. Determination of macrotime cutoffs and tracecount normalization.

Left: distribution of determined macrotime cutoffs. Right: log-log plot of normalized tracecounts per sample. Average macrotime-cutoffs are indicated as vertical lines. Average background (BG) thresholds are indicated as horizontal lines. See text for details.

The total trace counts after applying the macrotime- and intensity threshold are presented in Fig. S15 on the x-axis vs. the detected number of photobleaching steps on the y-axis. Here, the distribution of total counts for each number of bleaching step is highly skewed, as visible from the grey violin plot representation. Intriguingly, we can verify that mean values of these distributions rise approximately linear with increasing number of bleaching steps (indicated by red crosses). The slopes of these linear dependencies were then calculated by a principal component analysis (fit of red line) trough the origin.

Next to bleaching step and total trace count distributions, also the reduced sample mean values were determined and plotted, see Fig. S16 (middle and right). The sample mean values form two populations: one for CD86-mEGFP and CD95 samples and one for pseudodimer and $CTLA4_{DA}$ samples. Two samples have exceptionally high total count values: pseudodimer and $CTLA4_{DA}$ 1st run (see Fig. S16 (middle). It was noticed that during measurements of the latter samples, the excitation spot was displaced from the local spot maximum. This corresponds to an effectively lower excitation power. To compensate for this effect, the two $CTLA4_{DA}$ samples were compared and the data of the $CTLA4_{DA}$ 2nd run was used as a reference to calculate a correction factor to rescale pseudodimer and $CTLA4_{DA}$ 1st run data (see Fig. S16, right).

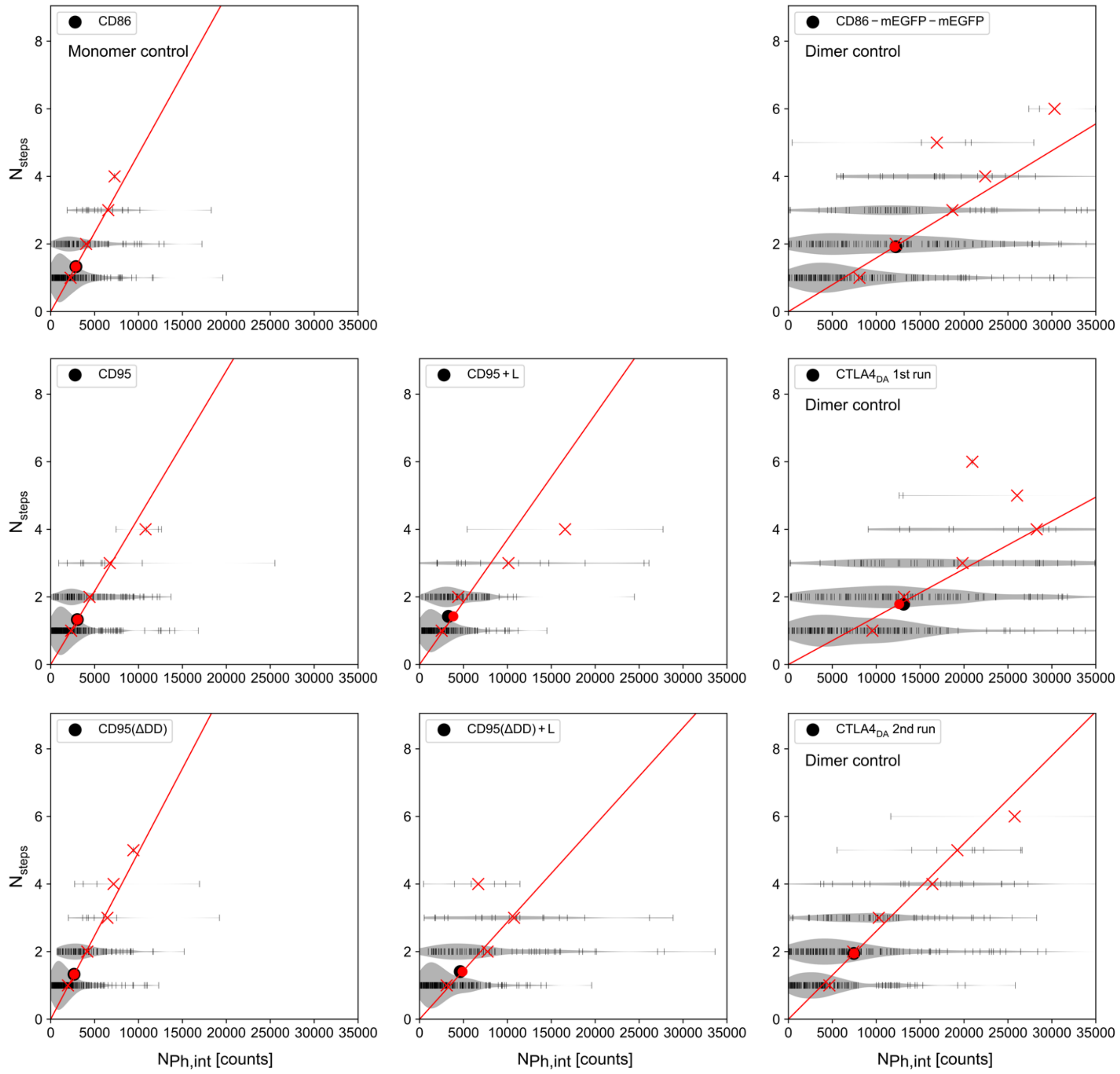

Fig. S15. Distribution of step numbers N_{steps} vs. integrated photon counts $N_{Ph,int}$ for all measured samples.

Step numbers were derived from the KV fit and photon counts from integrating all photons of a bleaching trace. Red lines are orthogonal regressions to mean trace total counts (red crosses) weighted by the number of data points (vertical black bars) for each N_{steps}. Black dots – mean values of raw data. Black error bars – Standard deviation of the population distribution of $N_{Ph,int}$ and N_{steps}, respectively. Red dots – mean values obtained as result of orthogonal regression through the origin.

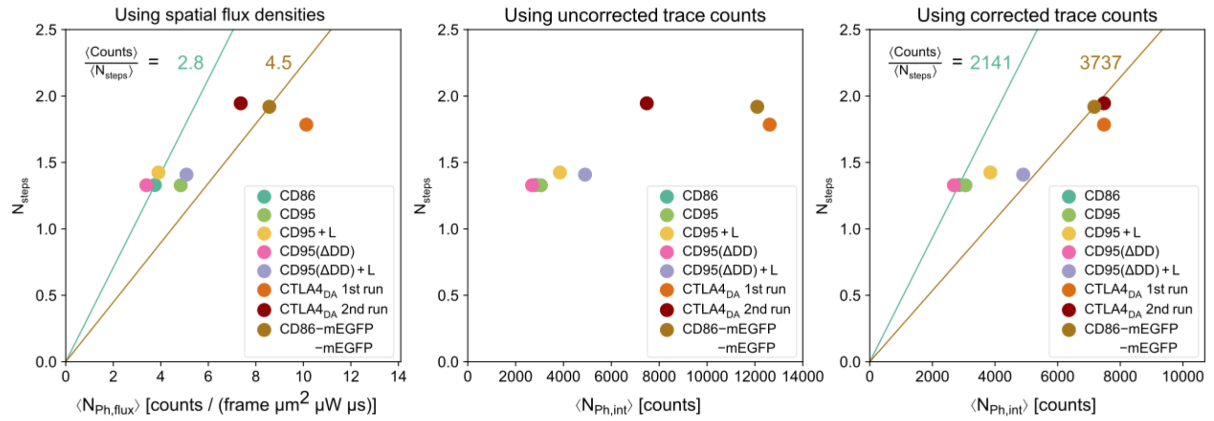

Fig. S16. Average step number as function of flux densities or integrated photon counts

Left: Average values of step number, $\langle N_{steps} \rangle$, vs. spatial flux densities of photons, $\langle N_{Ph,\text{flux}} \rangle$. Spatial flux densities of photons correspond to photon counts before bleaching in spot vicinities, registered per frame, and corrected for spot sizes, laser powers, and integration times. Middle: Average values of step number, $\langle N_{steps} \rangle$, vs. integrated photon counts, $\langle N_{Ph,int} \rangle$, per sample. Data is non-corrected. Right: Average values of step number, $\langle N_{steps} \rangle$, vs. integrated photon counts, $\langle N_{Ph,int} \rangle$, per sample. Data includes correction for macrotime and background intensity threshold as shown in Fig. S14. Lines indicate the average photon counts per step, exemplary for CD86 monomer and CD86-mEGFP-mEGFP dimer control.

Finally, two different analyses were followed, to relate the average step number, $\langle N_{steps} \rangle$, to the sample photon counts: on the one hand, the spatial flux densities of photons per frame were analyzed prior to bleaching (left). Here, the total number of photons in the vicinity of each bleaching spot was counted and normalised by the number of frames, the area, as well as the laser excitation power. The radius of the vicinity region was increased up to a maximum value of 2 µm, which is ~ 10 more the width of the laser excitation profile. On the other hand, $\langle \boldsymbol{N_{steps}} \rangle$ vs. total trace counts (middle and right) was analyzed. Total trace counts were analyzed using a background intensity substraction and a macrotime correction (see Fig. S14). Interestingly, spatial densities as well as total trace counts revealed similar sample behaviour: photon count densities increase with higher oligomerization of the receptor, but at the same time sensitively react to crowding effects. The obtained average number of steps as a function of count densities or total trace counts are presented in Fig. 4f (right) in the main text and in Fig. S16 (left and right). The data reveals clear separation of primarily monomeric receptors which may include a small fraction of higher oligomeric states, and pure dimeric receptors. Higher local crowding effects appear to be present in the CD95, CD95(ΔDD)+L as well as the $CTLA4_{DA}$ 1st run sample (Fig. S16 left). Note, that if all dyes in all systems were recorded at equivalent local conditions and exhibited similar photophysical properties, we would expect values to appear on the same line. However, the mean total counts between samples may vary, for example, due to different distributions of dye orientations, differential absorption or emission, local interactions between dyes, etc.

Supplementary Note 5: Determination of crowding factor

To determine a molecular crowding factor k_{crowd} arising from receptor density fluctuations on the cell membrane, we considered the $\lesssim 80\%$ (*46, 47*) maturation efficiency of mEGFP, which reduces the average number of fluorophores detected per spot and also the total number of receptors detected on the cell membrane. In case of the monomer control sample CD86, an average $\langle N_{\mathrm{steps}} \rangle = 1.33$ instead of $\langle N_{\mathrm{steps,\ ideal}} \rangle = 1$ for a perfect monomer sample was measured. Here, any deviation from the latter value can only arise from density fluctuations and needs to be corrected for the 80% visible and 20% invisible receptors. Together with the error in step size $\Delta N_{\mathrm{steps}}$:

$$\langle N_{\mathrm{steps}} \rangle \pm \Delta N_{\mathrm{steps}} = 1.33 \pm 0.6 = (0 \cdot 0.2 + 1 \cdot 0.8)/(0.8) \cdot k_{\mathrm{crowd}} \Rightarrow k_{\mathrm{crowd}} = 1.33 \pm 0.6$$

In case of CD86-mEGFP-mEGFP, $\langle N_{\mathrm{steps}} \rangle = 1.92$ instead of $\langle N_{\mathrm{steps,\ ideal}} \rangle = 2$ was measured. Also here, one receptor per fluorescent spot with two fluorophores would be expected, but higher number of receptors could be detected due to crowding effects. On the other hand, the $\lesssim 80\%$ (*46, 47*) maturation efficiency of mEGFP reduces the average number of fluorophores detected per spot. In particular, k_{crowd} is calculated as follows: 1. A binomial distribution is used to calculate the probability that 1 receptor with 1 mEGFP or that 1 receptor with 2 mEGFP is detected. Here, the probability for a matured mEGFP is taken as $p = 0.8$ and the probability for the non-fluorescent mEGFP is $(1 - p) = 0.2$ accordingly. The following table provides the probabilities of observe CD86-mEGFP-mEGFP with 1 or 2 mEGFP:

# of matured mEGFP on pseudodimer construct	Probability of receptor [0-1]
No mEGFP	$0.2^2 = 0.04$
1 mEGFP	$2 \cdot 0.8 \cdot 0.2 = 0.32$
2 mEGFP	$0.8^2 = 0.64$

$$\langle N_{\mathrm{steps}} \rangle \pm \Delta N_{\mathrm{steps}} = 1.92 \pm 1.0 = (0 \cdot 0.04 + 1 \cdot 0.32 + 2 \cdot 0.64)/(0.32 + 0.64) \cdot k_{\mathrm{crowd}} = 1.67 \cdot k_{\mathrm{crowd}} \Rightarrow k_{\mathrm{crowd}} = 1.15 \pm 0.6$$

As average value of these two CD86 samples $\langle k_{\mathrm{crowd}} \rangle = 1.24 \pm 0.42$ is obtained. While the average value $\langle k_{\mathrm{crowd}} \rangle = 1.24$ is well-defined (small standard error), the standard deviation shows that the $\langle k_{\mathrm{crowd}} \rangle$ value can vary within the sample population by up to 34%.

We further calculated the fraction of receptors which would not be affected by crowding, $f_{\mathrm{no\ crowd}}$. In case of CD86 this was obtained from calculating the sum of N_{steps} occuring, weighted by their probability (i.e. number of traces with this N_{steps}, divided by the total number of traces 324) and equating this against the theoretically expected fraction of crowding afflicted $\langle N_{\mathrm{steps,\ ideal}} \rangle = \frac{0 \cdot 0.2 + 1 \cdot 0.8}{0.8} = 1$. $\Delta f_{\mathrm{no\ crowd}}(CD86)$ calculated according to Gaussian error propagation:

$$f_{\mathrm{no\ crowd}}(CD86) = \frac{\langle N_{\mathrm{steps,\ ideal}} \rangle}{\langle N_{\mathrm{steps}} \rangle \pm \Delta N_{\mathrm{steps}}} = \frac{1}{(1 \cdot \frac{235}{324} + 2 \cdot \frac{74}{324} + 3 \cdot \frac{14}{324} + 4 \cdot \frac{1}{324}) \pm 0.6} = \frac{1}{1.33 \pm 0.6} = 0.75 \pm 0.45$$

Thus, on average 75% of measured CD86 traces would be free from any crowding effects.

In case of CD86-mEGFP-mEGFP a similar calculation with $\langle N_{\text{steps, ideal}} \rangle = (0 \cdot 0.04 + 1 \cdot 0.32 + 2 \cdot 0.64)/(0.32 + 0.64) = 1.67$ yields

$$f_{\text{no crowd}}(CD86 - \text{mEGFP} - \text{mEGFP}) = \frac{\langle N_{\text{steps, ideal}} \rangle}{\langle N_{\text{steps}} \rangle \pm \Delta N_{\text{steps}}} = \frac{1.67}{1.92 \pm 1.0} = 0.87 \pm 0.67.$$

Thus, on average 87% of measured CD86-mEGFP-mEGFP traces would be free from any crowding effects.

Again, while the average values $f_{\text{no crowd}}(CD86)$ and $f_{\text{no crowd}}(CD86 - \text{mEGFP} - \text{mEGFP})$ are well-defined, the standard deviations show that $f_{\text{no crowd}}$ values can vary within the sample population by up to 60%, and 77%, respectively.

REFERENCES AND NOTES

1. J. D. Scott, T. Pawson, Cell signaling in space and time: Where proteins come together and when they're apart. *Science* **326**, 1220–1224 (2009).

2. D. R. Green, *Cell Death: Apoptosis and Other Means to an End* (Cold Spring Harbor Laboratory Press, ed. 2, 2018).

3. P. H. Krammer, CD95's deadly mission in the immune system. *Nature* **407**, 789–795 (2000).

4. S. Kim, N. Kim, J. Lee, S. Kim, J. Hong, S. Son, W. D. Heo, Dynamic Fas signaling network regulates neural stem cell proliferation and memory enhancement. *Sci. Adv.* **6**, eaaz9691 (2020).

5. E. Bremer, Targeting of the tumor necrosis factor receptor superfamily for cancer immunotherapy. *ISRN Oncol* **2013**, 371854 (2013).

6. P. E. Morton, C. Perrin, J. Levitt, D. R. Matthews, R. J. Marsh, R. Pike, D. McMillan, A. Maloney, S. Poland, S. Ameer-Beg, M. Parsons, TNFR1 membrane reorganization promotes distinct modes of TNFα signaling. *Sci. Signal.* **12**, eaaw2418 (2019).

7. F. K. M. Chan, H. J. Chun, L. X. Zheng, R. M. Siegel, K. L. Bui, M. J. Lenardo, A domain in TNF receptors that mediates ligand-independent receptor assembly and signaling. *Science* **288**, 2351–2354 (2000).

8. V. Boschert, A. Krippner-Heidenreich, M. Branschadel, J. Tepperink, A. Aird, P. Scheurich, Single chain TNF derivatives with individually mutated receptor binding sites reveal differential stoichiometry of ligand receptor complex formation for TNFR1 and TNFR2. *Cell. Signal.* **22**, 1088–1096 (2010).

9. J. H. Naismith, T. Q. Devine, B. J. Brandhuber, S. R. Sprang, Crystallographic evidence for dimerization of unliganded tumor necrosis factor receptor. *J. Biol. Chem.* **270**, 13303–13307 (1995).

10. F. L. Scott, B. Stec, C. Pop, M. K. Dobaczewska, J. J. Lee, E. Monosov, H. Robinson, G. S. Salvesen, R. Schwarzenbacher, S. J. Riedl, The Fas–FADD death domain complex structure unravels signalling by receptor clustering. *Nature* **457**, 1019–1022 (2009).

11. E. S. Vanamee, G. Lippner, D. L. Faustman, Signal amplification in highly ordered networks is driven by geometry. *Cells* **11**, 272 (2022).

12. E. S. Vanamee, D. L. Faustman, Structural principles of tumor necrosis factor superfamily signaling. *Sci. Signal.* **11**, eaao4910 (2018).

13. L. W. Wang, J. K. Yang, V. Kabaleeswaran, A. J. Rice, A. C. Cruz, A. Y. Park, Q. A. Yin, E. Damko, S. B. Jang, S. Raunser, C. V. Robinson, R. M. Siegel, T. Walz, H. Wu, The Fas-FADD

death domain complex structure reveals the basis of DISC assembly and disease mutations. *Nat. Struct. Mol. Biol.* **17**, 1324-1329 (2010).

14. R. M. Siegel, J. R. Muppidi, M. Sarker, A. Lobito, M. Jen, D. Martin, S. E. Straus, M. J. Lenardo, SPOTS. *J. Cell Biol.* **167**, 735–744 (2004).

15. S. Weidtkamp-Peters, S. Felekyan, A. Bleckmann, R. Simon, W. Becker, R. Kuhnemuth, C. A. M. Seidel, Multiparameter fluorescence image spectroscopy to study molecular interactions. *Photochem. Photobiol. Sci.* **8**, 470–480 (2009).

16. E. Lerner, A. Barth, J. Hendrix, B. Ambrose, V. Birkedal, S. C. Blanchard, R. Börner, H. S. Chung, T. Cordes, T. D. Craggs, A. A. Deniz, J. Diao, J. Fei, R. L. Gonzalez, I. V. Gopich, T. Ha, C. A. Hanke, G. Haran, N. S. Hatzakis, S. Hohng, S.-C. Hong, T. Hugel, A. Ingargiola, C. Joo, A. N. Kapanidis, H. D. Kim, T. Laurence, N. K. Lee, T.-H. Lee, E. A. Lemke, E. Margeat, J. Michaelis, X. Michalet, S. Myong, D. Nettels, T.-O. Peulen, E. Ploetz, Y. Razvag, N. C. Robb, B. Schuler, H. Soleimaninejad, C. Tang, R. Vafabakhsh, D. C. Lamb, C. A. Seidel, S. Weiss, FRET-based dynamic structural biology: Challenges, perspectives and an appeal for open-science practices. *eLife* **10**, e60416 (2021).

17. B. N. Kholodenko, Cell-signalling dynamics in time and space. *Nat. Rev. Mol. Cell Biol.* **7**, 165–176 (2006).

18. S. M. Kallenberger, J. Beaudouin, J. Claus, C. Fischer, P. K. Sorger, S. Legewie, R. Eils, Intra- and interdimeric caspase-8 self-cleavage controls strength and timing of CD95-induced apoptosis. *Sci. Signal.* **7**, ra23 (2014).

19. S. K. Bromley, W. R. Burack, K. G. Johnson, K. Somersalo, T. N. Sims, C. Sumen, M. M. Davis, A. S. Shaw, P. M. Allen, M. L. Dustin, The immunological synapse. *Annu. Rev. Immunol.* **19**, 375–396 (2001).

20. C. Haist, E. Schulte, N. Bartels, A. Bister, Z. Poschinski, T. C. Ibach, K. Geipel, C. Wiek, M. Wagenmann, C. Monzel, K. Scheckenbach, H. Hanenberg, CD44v6-targeted CAR T-cells specifically eliminate CD44 isoform 6 expressing head/neck squamous cell carcinoma cells. *Oral Oncol.* **116**, 105259 (2021).

21. M. Soldierer, A. Bister, C. Haist, A. Thivakaran, S. C. Cengiz, S. Sendker, N. Bartels, A. Thomitzek, D. Smorra, M. Hejazi, M. Uhrberg, K. Scheckenbach, C. Monzel, C. Wiek, D. Reinhardt, N. Niktoreh, H. Hanenberg, Genetic engineering and enrichment of human NK cells for CAR-enhanced immunotherapy of hematological malignancies. *Front. Immunol.* **13**, 847008 (2022).

22. U. Uslu, T. Da, C. A. Assenmacher, J. Scholler, R. M. Young, J. Tchou, C. H. June, Chimeric antigen receptor T cells as adjuvant therapy for unresectable adenocarcinoma. *Sci. Adv.* **9**, (2023).

23. C. You, T. T. Marquez-Lago, C. P. Richter, S. Wilmes, I. Moraga, K. C. Garcia, A. Leier, J. Piehler, Receptor dimer stabilization by hierarchical plasma membrane microcompartments regulates cytokine signaling. *Sci. Adv.* **2**, e1600452 (2016).

24. R. Regmi, S. Srinivasan, A. P. Latham, V. Kukshal, W. D. Cui, B. Zhang, R. Bose, G. S. Schlau-Cohen, Phosphorylation-dependent conformations of the disordered carboxyl-terminus domain in the epidermal growth factor receptor. *J. Phys. Chem. Lett.* **11**, 10037–10044 (2020).

25. C. Monzel, A. S. Becker, R. Saffrich, P. Wuchter, V. Eckstein, A. D. Ho, M. Tanaka, Dynamic cellular phenotyping defines specific mobilization mechanisms of human hematopoietic stem and progenitor cells induced by SDF1α versus synthetic agents. *Sci. Rep.* **8**, 1841 (2018).

26. M. Rehm, H. Dussmann, R. U. Janicke, J. M. Tavare, D. Kogel, J. H. Prehn, Single-cell fluorescence resonance energy transfer analysis demonstrates that caspase activation during apoptosis is a rapid process. Role of caspase-3. *J. Biol. Chem.* **277**, 24506–24514 (2002).

27. K. Cosentino, A. J. Garcia-Saez, Mitochondrial alterations in apoptosis. *Chem. Phys. Lipids* **181**, 62–75 (2014).

28. I. N. Lavrik, P. H. Krammer, Regulation of CD95/Fas signaling at the DISC. *Cell Death Differ.* **19**, 36–41 (2012).

29. F. Fricke, J. Beaudouin, R. Eils, M. Heilemann, One, two or three? Probing the stoichiometry of membrane proteins by single-molecule localization microscopy. *Sci. Rep.* **5**, 14072 (2015).

30. K. Kucka, H. Wajant, Receptor oligomerization and its relevance for signaling by receptors of the tumor necrosis factor receptor superfamily. *Front. Cell Dev. Biol.* **8**, 615141 (2020).

31. G. S. Gülcüler Balta, C. Monzel, S. Kleber, J. Beaudouin, E. Balta, T. Kaindl, S. Chen, L. Gao, M. Thiemann, C. R. Wirtz, Y. Samstag, M. Tanaka, A. Martin-Villalba, 3D cellular architecture modulates tyrosine kinase activity, thereby switching CD95-mediated apoptosis to survival. *Cell Rep.* **29**, 2295–2306.e6 (2019).

32. C. Liesche, J. Berndt, F. Fricke, S. Aschenbrenner, M. Heilemann, R. Eils, J. Beaudouin, CD95 receptor activation by ligand-induced trimerization is independent of its partial pre-ligand assembly. bioRxiv 293530 [Preprint] (2018). https://doi.org/10.1101/293530.

33. G. C. Starling, J. Bajorath, J. Emswiler, J. A. Ledbetter, A. Aruffo, P. A. Kiener, Identification of amino acid residues important for ligand binding to Fas. *J. Exp. Med.* **185**, 1487–1492 (1997).

34. C. Kleusch, C. Monzel, K. C. Sridhar, B. Hoffmann, A. Csiszar, R. Merkel, Fluorescence correlation spectroscopy reveals interaction of some microdomain-associated lipids with cellular focal adhesion sites. *Int. J. Mol. Sci.* **21**, 8149 (2020).

35. M. Gerken, A. Krippner-Heidenreich, S. Steinert, S. Willi, F. Neugart, A. Zappe, J. Wrachtrup, C. Tietz, P. Scheurich, Fluorescence correlation spectroscopy reveals topological segregation of the two tumor necrosis factor membrane receptors. *BBA-Biomembranes* **1798**, 1081–1089 (2010).

36. K. Jaqaman, J. A. Galbraith, M. W. Davidson, C. G. Galbraith, Changes in single-molecule integrin dynamics linked to local cellular behavior. *Mol. Biol. Cell* **27**, 1561–1569 (2016).

37. T.-O. Peulen, O. Opanasyuk, C. A. Seidel, Combining graphical and analytical methods with molecular simulations to analyze time-resolved FRET measurements of labeled macromolecules accurately. *J. Phys. Chem. B.* **121**, 8211–8241 (2017).

38. G. Agam, C. Gebhardt, M. Popara, R. MSchtel, J. Folz, B. Ambrose, N. Chamachi, S. Y. Chung, T. D. Craggs, M. de Boer, D. Grohmann, T. Ha, A. Hartmann, J. Hendrix, V. Hirschfeld, C. G. Hübner, T. Hugel, D. Kammerer, H. S. Kang, A. N. Kapanidis, G. Krainer, K. Kramm, E. A. Lemke, E. Lerner, E. Margeat, K. Martens, J. Michaelis, J. Mitra, G. G. M. Muñoz, R. B. Quast, N. C. Robb, M. Sattler, M. Schlierf, J. Schneider, T. Schröder, A. Sefer, P. S. Tan, J. Thurn, P. Tinnefeld, J. van Noort, S. Weiss, N. Wendler, N. Zijlstra, A. Barth, C. A. M. Seidel, D. C. Lamb, T. Cordes, Reliability and accuracy of single-molecule FRET studies for characterization of structural dynamics and distances in proteins. *Nat. Methods* **20**, 523–535 (2023).

39. D. S. Lidke, P. Nagy, B. G. Barisas, R. Heintzmann, J. N. Post, K. A. Lidke, A. H. A. Clayton, D. J. Arndt-Jovin, T. M. Jovin, Imaging molecular interactions in cells by dynamic and static fluorescence anisotropy (rFLIM and emFRET). *Biochem. Soc. Trans.* **31**, 1020–1027 (2003).

40. N. R. Marzano, B. P. Paudel, A. M. van Oijen, H. Ecroyd, Real-time single-molecule observation of chaperone-assisted protein folding. *Sci. Adv.* **8**, eadd0922 (2022).

41. A. N. Bader, S. Hoetzl, E. G. Hofman, J. Voortman, P. M. P. van Bergen en Henegouwen, G. van Meer, H. C. Gerritsen, Homo-FRET imaging as a tool to quantify protein and lipid clustering. *Chemphyschem* **12**, 475–483 (2011).

42. R. K. Vishwakarma, A. M. Cao, Z. Morichaud, A. S. Perumal, E. Margeat, K. Brodolin, Single-molecule analysis reveals the mechanism of transcription activation in *M. tuberculosis*. *Sci. Adv.* **4**, eaao5498 (2018).

43. A. Greife, S. Felekyan, Q. J. Ma, C. G. W. Gertzen, L. Spomer, M. Dimura, T. O. Peulen, C. Woehler, D. Haussinger, H. Gohlke, V. Keitel, C. A. M. Seidel, Structural assemblies of the di- and oligomeric G-protein coupled receptor TGR5 in live cells: An MFIS-FRET and integrative modelling study. *Sci. Rep.* **6**, 36792 (2016).

44. M. Dimura, T. O. Peulen, H. Sanabria, D. Rodnin, K. Hemmen, C. A. Hanke, C. A. M. Seidel, H. Gohlke, Automated and optimally FRET-assisted structural modeling. *Nat. Commun.* **11**, 5394 (2020).

45. A. H. A. Clayton, A. Chattopadhyay, Taking care of bystander FRET in a crowded cell membrane environment. *Biophys. J.* **106**, 1227–1228 (2014).

46. M. H. Ulbrich, E. Y. Isacoff, Subunit counting in membrane-bound proteins. *Nat. Methods* **4**, 319–321 (2007).

47. V. Dunsing, M. Luckner, B. Zühlke, R. A. Petazzi, A. Herrmann, S. Chiantia, Optimal fluorescent protein tags for quantifying protein oligomerization in living cells. *Sci. Rep.* **8**, 1–12 (2018).

48. Q. S. Fu, T. M. Fu, A. C. Cruz, P. Sengupta, S. K. Thomas, S. Q. Wang, R. M. Siegel, H. Wu, J. J. Chou, Structural basis and functional role of intramembrane trimerization of the Fas/CD95 death receptor. *Mol. Cell* **61**, 602–613 (2016).

49. S. Clarke, F. Pinaud, O. Beutel, C. You, J. Piehler, M. Dahan, Covalent monofunctionalization of peptide-coated quantum dots for single-molecule assays. *Nano Lett.* **10**, 2147–2154 (2010).

50. J. Hummert, K. Yserentant, T. Fink, J. Euchner, Y. X. Ho, S. A. Tashev, D. P. Herten, Photobleaching step analysis for robust determination of protein complex stoichiometries. *Mol. Biol. Cell* **32**, ar35 (2021).

51. B. Kalafut, K. Visscher, An objective, model-independent method for detection of non-uniform steps in noisy signals. *Comput. Phys. Commun.* **179**, 716–723 (2008).

52. C. Eggeling, J. Widengren, R. Rigler, C. A. Seidel, Photobleaching of fluorescent dyes under conditions used for single-molecule detection: Evidence of two-step photolysis. *Anal. Chem.* **70**, 2651–2659 (1998).

53. D. J. Foust, A. G. Godin, A. Ustione, P. W. Wiseman, D. W. Piston, Two-color spatial cumulant analysis detects heteromeric interactions between membrane proteins. *Biophys. J.* **117**, 1764–1777 (2019).

54. C. Karathanasis, J. Medler, F. Fricke, S. Smith, S. Malkusch, D. Widera, S. Fulda, H. Wajant, S. J. L. van Wijk, I. Dikic, M. Heilemann, Single-molecule imaging reveals the oligomeric state of functional TNFα-induced plasma membrane TNFR1 clusters in cells. *Sci. Signal.* **13**, eaax5647 (2020).

55. L. E. Rodseth, B. Brandhuber, T. Q. Devine, M. J. Eck, K. Hale, J. H. Naismith, S. R. Sprang, Two crystal forms of the extracellular domain of type I tumor necrosis factor receptor. *J. Mol. Biol.* **239**, 332–335 (1994).

56. G. Papoff, P. Hausler, A. Eramo, M. G. Pagano, G. Di Leve, A. Signore, G. Ruberti, Identification and characterization of a ligand-independent oligomerization domain in the extracellular region of the CD95 death receptor. *J. Biol. Chem.* **274**, 38241–38250 (1999).

57. R. M. Siegel, J. K. Frederiksen, D. A. Zacharias, F. K. M. Chan, M. Johnson, D. Lynch, R. Y. Tsien, M. J. Lenardo, Fas preassociation required for apoptosis signaling and dominant inhibition by pathogenic mutations. *Science* **288**, 2354–2357 (2000).

58. D. W. Banner, A. Darcy, W. Janes, R. Gentz, H. J. Schoenfeld, C. Broger, H. Loetscher, W. Lesslauer, Crystal structure of the soluble human 55 kd TNF receptor-human TNFβ complex: Implications for TNF receptor activation. *Cell* **73**, 431–445 (1993).

59. J. L. Bodmer, P. Schneider, J. Tschopp, The molecular architecture of the TNF superfamily. *Trends Biochem. Sci.* **27**, 19–26 (2002).

60. C. R. Reis, A. H. G. van Assen, W. J. Quax, R. H. Cool, Unraveling the binding mechanism of trivalent tumor necrosis factor ligands and their receptors. *Mol. Cell. Proteomics* **10**, M110.002808 (2011).

61. F. Henkler, E. Behrle, K. M. Dennehy, A. Wicovsky, N. Peters, C. Warnke, K. Pfizenmaier, H. Wajant, The extracellular domains of FasL and Fas are sufficient for the formation of supramolecular FasL-Fas clusters of high stability. *J. Cell Biol.* **168**, 1087–1098 (2005).

62. D. Esposito, A. Sankar, N. Morgner, C. V. Robinson, K. Rittinger, P. C. Driscoll, Solution NMR investigation of the CD95/FADD homotypic death domain complex suggests lack of engagement of the CD95 C terminus. *Structure* **18**, 1378–1390 (2010).

63. N. Levoin, M. Jean, P. Legembre, CD95 structure, aggregation and cell signaling. *Front. Cell Dev. Biol.* **8**, 314 (2020).

64. R. M. L. Berger, J. M. Weck, S. M. Kempe, O. Hill, T. Liedl, J. O. Radler, C. Monzel, A. Heuer-Jungemann, Nanoscale FasL organization on DNA origami to decipher apoptosis signal activation in cells. *Small* **17**, 2101678 (2021).

65. C. Liesche, L. Venkatraman, S. Aschenbrenner, S. Grosse, D. Grimm, R. Eils, J. Beaudouin, Death receptor-based enrichment of Cas9-expressing cells. *BMC Biotechnol.* **16**, 17 (2016).

66. O. S. Qureshi, S. Kaur, T. Z. Hou, L. E. Jeffery, N. S. Poulter, Z. Briggs, R. Kenefeck, A. K. Willox, S. J. Royle, J. Z. Rappoport, D. M. Sansom, Constitutive clathrin-mediated endocytosis of CTLA-4 persists during T cell activation. *J. Biol. Chem.* **287**, 9429–9440 (2012).

67. J.-H. Budde, N. van der Voort, S. Felekyan, J. Folz, R. Kühnemuth, P. Lauterjung, M. Köhler, A. Schönle, J. Sindram, M. Otten, M. Karg, C. Herrmann, A. Barth, C. A. M. Seidel, FRET nanoscopy enables seamless imaging of molecular assemblies with sub-nanometer resolution. arXiv:2108.00024 [physics.optics] (30 July 2021).

68. J. Schindelin, I. Arganda-Carreras, E. Frise, V. Kaynig, M. Longair, T. Pietzsch, S. Preibisch, C. Rueden, S. Saalfeld, B. Schmid, J.-Y. Tinevez, D. J. White, V. Hartenstein, K. Eliceiri, P. Tomancak, A. Cardona, Fiji: An open-source platform for biological-image analysis. *Nat. Methods* **9**, 676–682 (2012).

69. S. Felekyan, R. Kuhnemuth, V. Kudryavtsev, C. Sandhagen, W. Becker, C. A. M. Seidel, Full correlation from picoseconds to seconds by time-resolved and time-correlated single photon detection. *Rev. Sci. Instrum.* **76**, 083104 (2005).

70. P. O. Gendron, F. Avaltroni, K. J. Wilkinson, Diffusion coefficients of several rhodamine derivatives as determined by pulsed field gradient-nuclear magnetic resonance and fluorescence correlation spectroscopy. *J. Fluoresc.* **18**, 1093–1101 (2008).

71. T. Wohland, R. Rigler, H. Vogel, The standard deviation in fluorescence correlation spectroscopy. *Biophys. J.* **80**, 2987–2999 (2001).

72. U. Kubitscheck, *Fluorescence Microscopy: From Principles to Biological Applications* (Wiley-VCH, ed. 2, 2017).

73. S. Baker, R. D. Cousins, Clarification of the use of CHI-square and likelihood functions in fits to histograms. *Nucl. Instrum. Methods. Phys. Res. B* **221**, 437–442 (1984).

74. M. Maus, M. Cotlet, J. Hofkens, T. Gensch, F. C. De Schryver, J. Schaffer, C. A. Seidel, An experimental comparison of the maximum likelihood estimation and nonlinear least-squares fluorescence lifetime analysis of single molecules. *Anal. Chem.* **73**, 2078–2086 (2001).

75. D. Stoyan, H. Stoyan, Estimating pair correlation functions of planar cluster processes. *Biom. J.* **38**, 259–271 (1996).

76. D. B. Peckys, U. Korf, N. de Jonge, Local variations of HER2 dimerization in breast cancer cells discovered by correlative fluorescence and liquid electron microscopy. *Sci. Adv.* **1**, e1500165 (2015).

77. Scientific Volume Imaging, Backprojected pinhole calculator, 2021; https://svi.nl/Olympus_FV1000.

78. P. J. Cranfill, B. R. Sell, M. A. Baird, J. R. Allen, Z. Lavagnino, H. M. de Gruiter, G.-J. Kremers, M. W. Davidson, A. Ustione, D. W. Piston. Quantitative assessment of fluorescent proteins. *Nat. Methods* **13**, 557–562 (2016).

79. E. O. Potma, W. P. de Boeij, L. Bosgraaf, J. Roelofs, P. J. M. van Haastert, D. A. Wiersma, Reduced protein diffusion rate by cytoskeleton in vegetative and polarized dictyostelium cells. *Biophys. J.* **81**, 2010–2019 (2001).
